# Reaping the Chemical Diversity of *Morinagamyces vermicularis* Using Feature-Based Molecular Networking

**DOI:** 10.1021/acs.jnatprod.4c00654

**Published:** 2024-09-16

**Authors:** Karen Harms, Esteban Charria-Girón, Alberto Miguel Stchigel, Yasmina Marin-Felix, Frank Surup

**Affiliations:** † Department Microbial Drugs, Helmholtz Centre for Infection Research, Inhoffenstrasse 7, 38124 Braunschweig, Germany; ‡ Institute of Microbiology, Technische Universität Braunschweig, Spielmannstrasse 7, 38106 Braunschweig, Germany; ∥ Mycology Unit, Medical School, Universitat Rovira i Virgili, C/Sant Llorenç 21, Tarragona, Reus, 43201, Spain

## Abstract

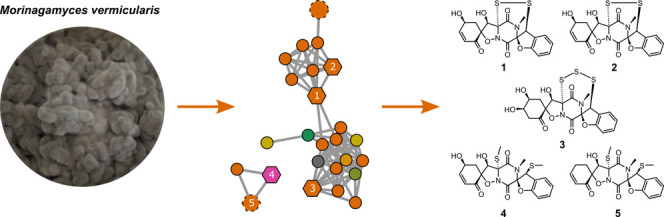

Moringadepsin (**6**) and chaetone B (**7**) were isolated by us in the course of a conventional chemical screening of *Morinagamyces vermicularis* CBS 303.81, a fungus belonging to the relatively underexplored family Schizotheciaceae of the phylum Ascomycota. Since these metabolites did not account for the antifungal activity observed in a crude extract of this fungus, we utilized an MS/MS-based molecular networking approach to get a thorough insight into the secondary metabolites produced by this strain. Manual annotation of high-resolution fragmentation mass spectra by CANOPUS classified a major molecular family as putatively new thiodiketopiperazines. However, these results were opposite to the results of ChemWalker analysis based solely on MS/MS data, assigning these metabolites as various polyketides. Thus, targeted preparative HPLC isolation focusing on the most abundant features within this major molecular family resulted in the isolation of five secondary metabolites. Their structures were elucidated based on HRMS and NMR data as four new thiodiketopiperazine derivatives, botryosulfuranols D–G (**1**–**4**), alongside the known botryosulfuranol A (**5**). Compounds **1**–**3** and **5** exhibited moderate to weak antifungal activity against different test strains, accounting for the initial antifungal activity observed for its crude extract. Our study stressed the importance of full NMR-based structure elucidation for metabolomics research.

The order Sordariales is one of the most diverse in the class Sordariomycetes (phylum Ascomycota, kingdom Fungi) and denotes a source of prolific producers of diverse biologically active secondary metabolites with potential applications.^1,2^ In the past decade, there have been plenty of examples of bioactive secondary metabolites isolated from taxa of this order.^3−7^ However, the number of genera from which nearly no information about the production of secondary metabolites is indeed surprising.^2^ For instance, species belonging to the Schizotheciaceae, one of the largest families in the Sordariales, has been neglected in terms of their secondary metabolite production, and only few reports are available.^5,8,9^

During our ongoing project focusing on the discovery of novel metabolites from taxa within the order Sordariales, a conventional chemical screening approach with the ex-type strain *Morinagamyces vermicularis* CBS 303.81 resulted in the isolation of the depsipeptide morinagadepsin (**6**) alongside chaetone B (**7**).^5^ While testing crude extracts of CBS 303.81 for antifungal activities with the indicator organisms *Mucor plumbeus* and *Candida tenuis*, we observed bioactivity against both fungi (MIC 75 and 300 μg/mL, respectively). These activities could not be explained by the production of **6** or **7**. Recent advancements in MS/MS-based metabolomics have the potential to revolutionize the discovery of natural products,^10^ offering detailed insights into the metabolite composition of complex mixtures or extracts.^11,12^ Consequently, we used a MS/MS-based molecular networking approach to inspect in detail the metabolome of this fungus and identify those metabolites responsible for the observed biological activity. Herein, we report the isolation of new polythiodiketopiperazine derivatives guided by the results of our MS/MS-based approach and the evaluation of their antimicrobial and cytotoxic activities.

*Morinagamyces vermicularis* was cultured in solid rice medium (BRFT) for 15 days, and the resulting crude extracts were analyzed by ultrahigh performance liquid chromatography coupled to diode array detection and ion mobility tandem mass spectrometry (UHPLC-DAD-IM-MS/MS). After raw data files were imported into MetaboScape software for processing, *t*-test analysis was conducted to compare features produced during solid cultivation to those in the medium extract, resulting in the prioritization of 197 MS-level features for further analysis. Among these, 172 were detected at the MS/MS level and clustered into 18 molecular families (MFs) with at least 2 connected nodes and 76 singletons (Figure 1A). Afterwards, we decided to examine the chemical diversity within the obtained molecular network (MN). For this purpose, we used CANOPUS, a computational tool integrated into the SIRIUS5 pipeline,^13^ useful to predict compound classes from fragmentation spectra based on the ClassyFire ontology.^14^ Despite the fact that 57 features remained unmatched to any chemical class, carboxylic acids and derivatives (44), peptidomimetics (14), and phenol ethers (9) were the most abundant chemical classes (Figure 1B). Notably, two major MFs containing 22 and 13 features were attributed to the chaetone B and morinagadepsin compound classes, respectively, after annotation propagation by comparison with reference spectra of these metabolites. An additional MF comprising 22 features, primarily classified as carboxylic acids and derivatives, remained unexplored; however, it comprised some of the most abundant compounds within the crude extract (Figure 1C).

**Figure 1:**
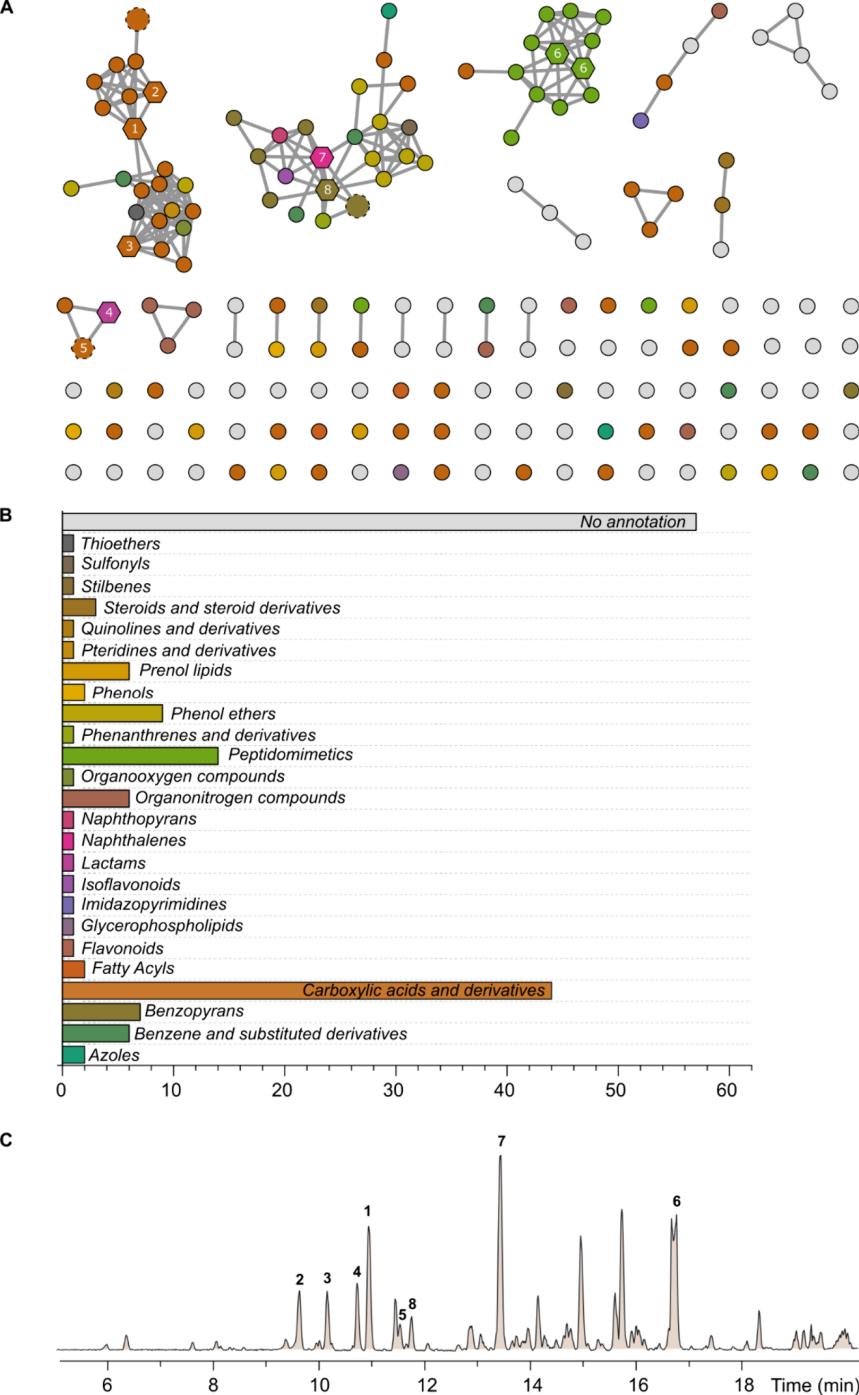
Feature-based molecular networking for the crude extracts obtained after cultivation of *M. vermicularis* CBS 303.81 in solid rice medium (BRFT), generated with the GNPS2 platform. Annotated metabolites are represented by dashed-lined nodes, while isolated metabolites are depicted by hexagon-shaped nodes (A). Nodes are colored according to the most specific classes (ClassyFire) obtained from CANOPUS analysis, with the bar plot indicating the number of features per chemical class (B). Base peak chromatogram of the BRFT crude extract obtained after the cultivation of *M. vermicularis* CBS 303.81 with isolated metabolites indicated by bold numbers over the corresponding peaks (C).

Further analysis of the CANOPUS results suggested that 17 of the features within this MF were classified as thiodiketopiperazines. Among them, one node, represented by an ion at *m*/*z* 433.0522 and a molecular formula of C_19_H_16_N_2_O_6_S_2_, was annotated as botryosulfuranol C. Examination of two other MFs with nodes matched as thiodiketopiperazines allowed us to annotate a node represented by ions at *m*/*z* 485.0810 and 463.0906, with a molecular formula of C_21_H_22_N_2_O_6_S_2_, as botryosulfuranol A. These compounds, both thiodiketopiperazines harboring two spirocyclic centers, were previously isolated from the endophytic fungus *Botryosphaeria mamane* (Botryosphaeriales, Ascomycota) and have not been reported before from members of the Sordariales.^15^

To explore the presence of other nodes belonging to these MFs in natural product structural databases such as COCONUT,^16^ we employed the ChemWalker tool,^17^ which uses combinatorial in silico fragmentation results (MetFrag) to mine biologically relevant databases and propagate annotations within spectral networks. We started by determining whether ChemWalker might validate the annotations of botryosulfuranol A and C. For the node represented by an ion at *m*/*z* 433.0522 (botryosulfuranol C), ChemWalker provided six different candidate structures with scores ranging from 0.296 to 1.0. However, these annotations included different substitutions such as bromide, chloride, fluoride, or none, which did not fit the isotopic pattern of the respective feature or the molecular formulas predicted by SIRIUS (Table S1). Similarly, for the node represented by an ion at *m*/*z* 485.0810 (botryosulfuranol A), ChemWalker analysis produced a similar poor output, failing to meet our dereplication criteria. Since this tool relies on in silico fragmentation of structure libraries, it is susceptible to errors, as MS/MS matching alone does not always lead to confident annotations.

In order to understand why botryosulfuranols A and C appeared clustered into different MFs during the FBMN analysis, we studied the MS/MS fragmentation pattern of these compounds. The main structural difference between both lies in the opening of the sulfur bridge, resulting in distinct MS/MS fragmentation patterns. Botryosulfuranol A as well as the other connected nodes in the MF generate common fragment ions at 361.03 328.05, 314.04, 281.05, 244.04, and 197.04 Da, which represent the most abundant fragments in their spectra. On the other hand, botryosulfuranol C generated fragment ions at 194.05, 180.98, 149.01, 137.01, and 127.05 Da. Based on the previous analysis, we hypothesized that botryosulfuranol congeners presenting a closed sulfur bridge were more common than those with the open bridge. Additionally, within these MFs, the most common modifications were represented by mass differences of 18 and 32 Da, interpreted as H_2_O and S loss/addition, respectively.

Motivated by these findings and the fact that these metabolites were demonstrated to display varying cytotoxic effects against cancerous and noncancerous cell lines, we decided to further investigate the nature of the compounds within these thiodiketopiperazines MFs and their relation to the observed antifungal activity.

## Isolation and Structure Elucidation

Fractionation of the crude extract, obtained from the cultivation in BRFT solid medium by both reversed-phase and normal-phase preparative HPLC, resulted in the isolation of the main metabolites **1**–**8** produced by *M. vermicularis* CBS 303.81. Compounds **1**–**4** are thiodiketopiperazines related to botryosulfuranol A (**5**) and botryosulfuranol C.^15^ In addition, previously described morinagadepsin (**6**) as well as known chaetones D (**7**) were also isolated and identified by comparison of their ^1^H and ^13^C NMR data to the literature.^5,15,18^

The main metabolite **1** was shown to have a molecular formula of C_19_H_16_N_2_O_7_S_2_ according to the molecular ion cluster at *m*/*z* 449.0468, indicating 13 degrees of unsaturation. Compared to botryosulfuranol A (**5**), this accounts for an additional degree of unsaturation and an additional oxygen atom as well as formal loss of a C_2_H_6_ unit. Only one *N*-methyl group but no *S*-methyl groups was detected in ^1^H and HSQC spectra.
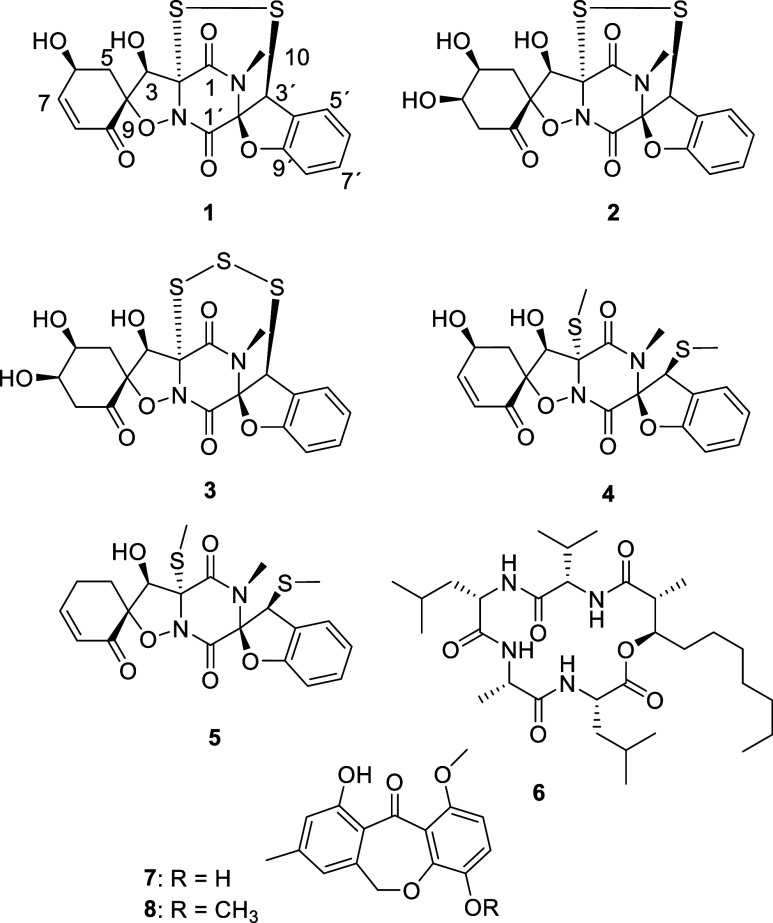


**Figure 2:**
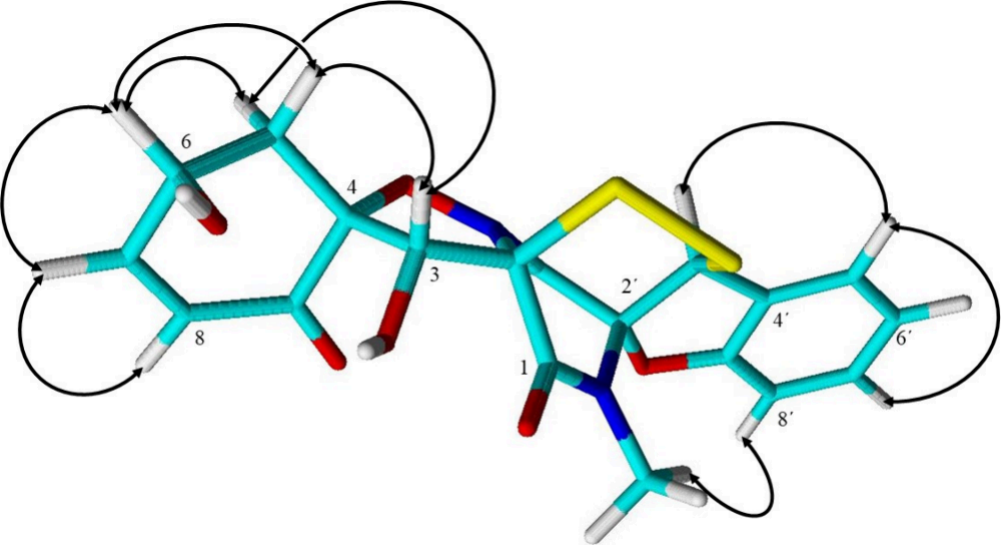
ROESY correlations indicative for the configurational assignment of botryosulfuranol D (**1**).

Consequently, a disulfite bridge was deduced, which is also part of botrysulfuranol C, a compound predicted but not isolated to purity in the present study.^15^ Additionally, an extra oxymethine replaced methylene CH_2_-6. The assignment of the stereoconfiguration of **1** was facilitated by the known configuration of **5**, which had been determined by both X-ray analysis as well as an evaluation of experimental to computed CD data.^15^ Based on similar CD data (Figure S48) and their common biosynthetic origin, we conclude a common 2*R*,3*R*,4*R*,2′*R*,3′*S* absolute configuration of the botryosulfuranols **1**–**5**. Lastly, ROESY correlations between H-3 and both H-5a and H-5b as well as between H-6 and both H-5a and H-5b (Figure 2) suggest a 6*S* configuration.

**Figure 3:**
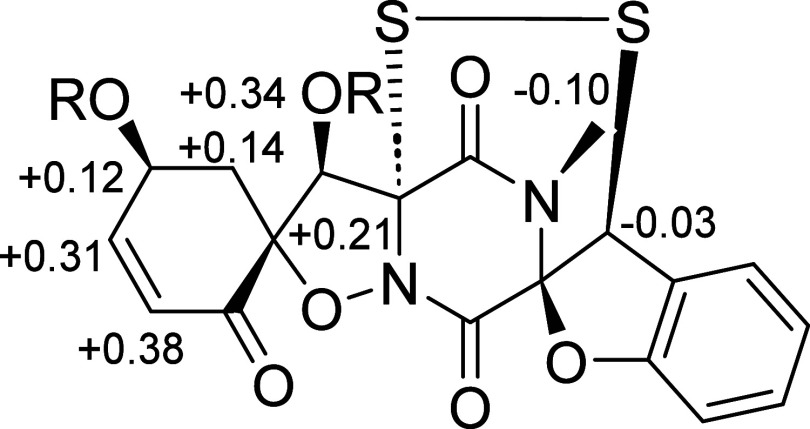
^1^H Δδ^SR^ values (in ppm) for the bis-MTPA derivative of **1**. R = (*S*)-MTPA for **1a** and (*R*)-MTPA for **1b**.

This assignment was confirmed by using Mosher’s method (Figure 3). Treatment with MTPA chlorides esterified both hydroxyl functions at C-3 and C-6. Consequently, the introduction of two units of MTPA as the auxiliary CDA on the substrate made it inoperative to just utilize the Δδ^SR^ configuration correlations developed for monofunctional compounds. However, a characteristic pattern can be evaluated.^19^ Positive values of Δδ^*SR*^ shift differences for H-6, H-7, and H-8 and a negative one for N–Me indicated 3*R* and 6*S* configurations, respectively. Due to its structural similarity to the known botrysufuranols, we suggest the name botryosulfuranol D for compound **1**.

HRESI-MS data of **2**, named botryosulfuranol E, specified a molecular formula of C_19_H_18_N_2_O_8_S_2_, indicating the formal addition of H_2_O and hence the loss of one unit of unsaturation. The NMR data of **2** was highly similar to that of **1** (Table 1). Key differences were the replacement of olefinic methines H-7 and H-8 by an oxymethine and a methylene. The oxymethine was placed at C-7 due to the COSY correlations between H-7 and H-6 as well H-7 and H-8a/H-8b. Similar to **1**, H-3 showed strong ROESY correlations to both H-5a/H-5b. A strong correlation between H-6 and H-8a indicated these protons to be pseudoaxial and on the same side of the six-membered ring system. Further ROESY correlations between H-7 and both H-6 as well as H-8a refer to a 6*S*,7*R* configuration.

**Table 1:** NMR Data (^1^H 700 MHz, ^13^C 175 MHz) for Compounds **1**–**5**

	**1**^a^		**2**^a^		**3**^b^		**4**^a^		**5**^a^	
no.	δ_C_, type	δ_H_, (mult, *J*, Hz)	δ_C_, type	δ_H_, (mult, *J*, Hz)	δ_C_, type	δ_H_, (mult, *J*, Hz)	δ_C_, type	δ_H_, (mult, *J*, Hz)	δ_C_, type	δ_H_, (mult, *J*, Hz)
1	164.9, C		164.1, C		162.0, C		163.7, C		162.1, C	
2	71.0, C		72.8, C		79.6, C		77.0, C		72.4, C	
3	86.3, CH	4.54, s	85.7, CH	4.55, s	82.9, CH	5.63, d (5.8)	88.1, CH	5.19, d (3.5)	86.2, CH	4.90, d (3.9)
4	83.0, C		85.8, C		93.4, C		85.9, C		87.7, C	
5	37.8, CH_2_	2.68, dd (14.1, 4.6)	34.6, CH_2_	2.65, dd (14.7, 7.9)	37.5, CH_2_	2.64, dd (14.6, 3.4)	38.6, CH_2_	2.65, d (5.0)	31.1, CH_2_	2.57, m
	37.8, CH_2_	2.43, dd (14.1, 4.2)	34.6, CH_2_	2.21, dd (14.7, 4.1)	37.5, CH_2_	2.01, dd (14.6, 2.6)			31.1, CH_2_	2.47, m
6	63.4, CH	4.67, pseudo q (4.4)	67.8, CH	4.36, ddd (7.9, 4.1, 3.1)	69.9, CH	4.25, br s	63.7, CH	4.75, m	24.1, CH_2_	2.69, m
7	150.7, CH	7.13, m	70.2, CH	4.16, ddd (7.3, 4.1, 3.1)	71.2, CH	3.98, m	149.4, CH	6.99, m	149.7, CH	7.01, m
8	129.5, CH	6.09, d (10.1)	44.5, CH_2_	2.98, dd (15.6, 4.1)	46.4, CH_2_	3.16, t (12.7)	129.5, CH	6.1, d (10.2)	129.6, CH	6.13, dt (10.1, 1.9)
			44.5, CH_2_	2.76, dd (15.6, 7.3)	46.4, CH_2_	2.51, ddd (12.7, 5.2, 1.5)				
9	194.6, C		201.8, C		198.5, C		193.0, C		192.3, C	
1′	157.9, C		158.2, C		157.1, C		160.5, C		159.8, C	
2′	96.1, C		96.1, C		99.7, C		97.6; C		97.7, C	
3′	56.5, CH	5.36, s	56.3, CH	5.31, s	75.4, CH	5.98, s	56.9, CH	4.94, s	57.4, CH	4.98, s
4′	120.9, C		120.8, C		123.9, C		125.1, C		125.7, C	
5′	124.8, CH	7.26, s	124.7, CH	7.24, br d (7.8)	124.6, CH	7.22, br d (7.7)	124.0, CH	7.29, m	124.7, CH	7.30, d (7.7)
6′	123.9, CH	7.11, br d (7.1)	123.9, CH	7.10, m	124.1, CH	7.14, br t (7.7)	122.6, CH	7.03, m	122.5, CH	7.02, m
7′	131.7, CH	7.39, t (8.0)	131.7, CH	7.38, br t (8.2)	132.1, CH	7.41, t (8.0)	130.2, CH	7.26, m	130.2, CH	7.25, m
8′	111.2, CH	7.16, d (8.2)	111.1, CH	7.12, d (8.2)	111.1, CH	7.07, d (8.2)	110.1, CH	7.01, m	110.0, CH	6.98, d (8.0)
9′	156.9; C		156.9, C		159.1, C		156.8, C		156.8, C	
N-Me	27.3, CH_3_	3.07 s	27.3, CH_3_	3.01, s	29.6, CH_3_	2.69, s	30.6, CH_3_	3.06, s	30.6, CH_3_	2.99, s
2-SMe							13.9, CH_3_	2.45, s	14.0, CH_3_	2.43, s
3′-SMe							15.9, CH_3_	2.10, s	16.7, CH_3_	2.21, s
3-OH						6.08 d (5.8)				4.74, br d (3.9)

a Measured in CHCl_3_-*d*.

b Measured in acetone-*d*_6_.

The molecular formula of **3**, for which we suggest the name botryosulfuranol F, was assigned as C_19_H_18_N_2_O_8_S_3_ by HRESI-MS data, which results in the presence of an additional sulfur atom in **3** compared to **2**. The NMR data of **3** were nearly identical with those of **2**. However, carbon resonances of C-2 and C-3′ were significantly shifted downfield in the ^13^C NMR spectrum. There are examples of other natural products such as outovirin C, emethallicin D, and penicisulfuranol C where the carbon resonance is shifted downfield due to a trisulfide bridge.^20−22^ Therefore, carbons C-2 and C-3′ were assigned with a trisulfide bridge.

Botryosulfuranol G (**4**) was shown to have a molecular formula of C_21_H_22_N_2_O_7_S_2_ based on HRESI-MS data, indicating an additional oxygen atom compared to botryosulfuranol A (**5**). NMR data were highly identical with those of **5**, but similar to **1**, an additional oxymethine was placed at C-6. Since chemical shifts were highly similar to those of **1** and **2**, a common configuration was deduced.

The botryosulfuranols D–F and A were evaluated for their antimicrobial and cytotoxic properties. Botrysulfuranol G could not be evaluated because not enought substance was obtained for testing. The compounds showed weak to moderate activity against Gram-positive bacteria and fungi (Table 2). In particular, botryosulfuranol D (**1**) exhibited the strongest effect against *Mucor hiemalis*. These activities likely explain the observed activity of the crude extract in our initial screening against *M. plumbeus*. In contrast to many other epithiodiketopiperazines, botryosulfuranols **1**–**3** and **5** exhibited only weak cytotoxic effects against the tested cell lines HeLa cells KB 3.1 and mouse fibroblast L929 (Table 3). The influence of the importance of the disulfide bridge for the cytotoxic activity remains inconclusive. Similar to previous results for botrysulfuranols A–C, the presence of a di- or trisulfide bridge did not correspond with increased cytotoxicity in our experiments.^15^ This is in contrast to the penicisulfuranols A–F, with disulfide bridge-containing compounds being much more cytotoxic compared to their opened up derivatives.^22^ On the other hand, **1** with its disulfide bridge exhibited the strongest activity against fungi and bacteria in our test panel.^22^

**Table 2:** Minimum Inhibitory Concentration (MIC, μg/mL) of **1**–**3** and **5** against Several Bacterial and Fungal Strains^a^

test organism	**1**	**2**	**3**	**5**	positive control
*Candida albicans*	66.6	–	66.6	–	16.6^N^
*Mucor hiemalis*	8	33.3	66.6	–	8.3^N^
*Rhodotorula glutinis*	33.3	66.6	66.6	–	2.1^N^
*Schizosaccharomyces pombe*	66.6	66.6	66.6	–	4.2^N^
*Wickerhamomyces anomalus*	33.3	66.6	33.3	–	8.3^N^
*Acinetobacter baumannii*	–	–	–	–	0.53^C^
*Bacillus subtilis*	16.6	33.3	66.6	66.6	16.6°
*Chromobacterium violaceum*	16.6	–	–	–	0.83°
*Escherichia coli*	–	–	–	–	3.3°
*Pseudomonas aeruginosa*	–	–	–	–	0.42^G^
*Staphylococcus aureus*	8.3	33.3	–	66.6	0.83°

a G, gentamicin; O, oxytetracycline; N, nystatin; C, ciprobay. −: no inhibition observed under test conditions.

**Table 3:** Cytotoxicity of **1**–**3** and **5** against Mammalian Cell Lines

	IC_50_ [μM]				
cell line	**1**	**2**	**3**	**5**	epothilone B
HeLa cells KB 3.1	49	54	-	15	4.0 × 10^–5^
mouse fibroblast L929	42	58	50	5	5.2 × 10^–4^

Epithiodiketopiperazines represent a widely distributed class of secondary metabolites produced via nonribosomal peptide synthetase (NRPS) biosynthetic pathways, exhibiting diverse biological activities.^23^ In the case of the botryosulfuranols, the presence of a sulfur bridge appears to influence their biological properties. Botryosulfuranols D and F, featuring a closed sulfur bridge, demonstrated more potent antimicrobial effects compared to botryosulfuranol A, which possesses an open sulfur bridge. Conversely, the latter compound exhibited the most pronounced cytotoxic effects among all of the compounds tested. It has been shown that diketopiperazine can undergo alternative pathways leading to the formation of the disulfide bridge or to the bismethylation of the dithiol precursor as in the case of acetylaranotin in *Aspergillus terreus*.^24^ Future efforts will be required to elucidate the biosynthetic origin of botryosulfuranols and its diversity among different fungal lineages.

Overall, we isolated four new epithiodiketopiperazines in the course of our study with primarily antifungal activities, highlighting the importance of integrating both old-school and state-of-the-art approaches in current natural product discovery approaches. By combining the strengths of traditional methods with modern bioinformatics tools, we can unravel the chemical diversity of fungal secondary metabolomes and systematically explore their biological activities.

## Experimental Section

### General Experimental Procedures

Optical rotations were measured using an Anton Paar MCP-150 polarimeter with a 100 mm path length and a sodium D line at 589 nm. UV spectra were measured on a Shimadzu UV/vis 2450 spectrophotometer using methanol (Uvasol, Merck) as a solvent. ECD spectra were measured with a J-815 spectropolarimeter (Jasco) using methanol as a solvent. The 1D and 2D nuclear magnetic resonance (NMR) spectra of isolated compounds were recorded with an Avance III 700 spectrometer equipped with a 5 mm TXI cryoprobe (Bruker, ^1^H NMR 700 MHz, ^13^C 175 MHz, Billerica, MA, USA) and an Avance III 500 (Bruker, ^1^H 500 MHz, ^13^C 125 MHz, Billerica, MA, USA) spectrometer, respectively. The chemical shifts δ were referenced to the solvents CHCl_3_-*d* (^1^H, δ = 7.27; ^13^C, δ = 77.00) and acetone-*d*_6_ (^1^H, δ = 2.05; ^13^C, δ = 29.32). Crude extracts and pure compounds were dissolved to concentrations of 4.5 and 1 mg/mL, respectively, in an acetone and methanol solution (1:1). Then, HPLC-DAD/MS measurements were performed using an amaZon speed ETD (electron transfer dissociation) ion trap mass spectrometer (Bruker Daltonics) and measured in positive and negative ion modes simultaneously. HPLC data were recorded on a column C18 Acquity UPLC BEH (Waters) using the following: solvent A H_2_O; solvent B acetonitrile (MeCN) supplemented with 0.1% formic acid, gradient conditions 5% B for 0.5 min, increasing to 100% B in 20 min, maintaining isocratic conditions at 100% B for 10 min, flow rate 0.6 mL/min, UV/vis detection 200–600 nm. HR-ESIMS (high-resolution electrospray ionization mass spectrometry) data were recorded on a MaXis ESI-TOF (electrospray ionization-time-of-flight) mass spectrometer (Bruker Daltonics, Bremen, Germany) coupled to an Agilent 1260 series HPLC-UV system and equipped with a C18 Acquity UPLC BEH (ultraperformance liquid chromatography) (ethylene-bridged hybrid) (Waters) column; DAD-UV detection at 200–600 nm; solvent A, water + 0.1% formic acid; solvent B, acetonitrile + 0.1% formic acid; flow rate 0.6 mL/min, 40 °C, gradient elution system with the initial conditions: 5% B for 0.5 min, increasing to 100% B in 19.5 min, and holding at 100% B for 5 min.

### Fermentation and Extraction

*Morinagamyces vermicularis* CBS 303.81 was cultivated and extracted as previously described.^5^ In total, four cultivations with 47 culture flasks and around 1.3 g of rice were cultured to yield 1.2 g of methanol extract.

### Metabolomics Analysis

Each sample was analyzed at a concentration of 450 μg/mL using an ultrahigh-performance liquid chromatography system (Dionex Ultimate3000RS, Thermo Scientific, Dreieich, Germany) equipped with a C18 column (Kinetex 1.7 μm, 2.1 × 150 mm, 100 Å; Phenomenex, Aschaffenburg, Germany) with an injection volume of 2 μL. The mobile phase consisted of solvent A (H_2_O + 0.1% formic acid) and solvent B (MeCN + 0.1% formic acid) at a constant flow rate of 0.3 mL/min. The gradient started with 1% B for 0.5 min, increased to 5% B within 1 min, and reached 100% B over 19 min, holding at 100% B for 5 min. The column temperature was kept at 40 °C, and UV–vis data were collected using a diode array detector (DAD) in the range of 190–600 nm. Mass spectra (MS) were acquired using a trapped ion mobility quadrupole time-of-flight mass spectrometer (timsTOF Pro, Bruker Daltonics, Bremen, Germany) with the following settings: tims ramp time 100 ms, spectra rate 9.52 Hz, PASEF on, cycle time 320 ms, MS/MS scans 2, scan range *m*/*z* 100–1800 Da; 1/*k*_0_, 0.55–2.0 V·s/cm^2^.

MS spectra were acquired in positive ion mode, and raw data were preprocessed with MetaboScape 2022 (Bruker Daltonics, Bremen, Germany) within the retention time range from 1.0 to 20 min.^11^ The obtained features were dereplicated based on their accurate molecular weight and MS/MS spectra and compared against compounds previously reported for ascomycetes in the Natural Product Atlas (NP Atlas) database.^25^ For this purpose, MetaboScape conducts automatic in silico MS/MS matching using the InChI-encoded structures via the MetFrag algorithm in the absence of MS/MS reference data.^26^ Molecular networks were created with the FBMN workflow on the GNPS platform using the preprocessed feature table from MetaboScape as described by Charria-Girón and co-workers.^27−29^ The spectra in the network were then searched against the GNPS spectral libraries. The molecular networks were visualized using Cytoscape software.^30^ Additionally, ChemWalker was used to propagate spectral annotations within different MFs.^17^

### Isolation

For compound purification, 1.1 g of the methanol crude extract was dissolved in methanol and portioned into 7 portions. The separation was done by using a PLC 2250 preparative HPLC system (Gilson, Middleton, WI, USA) with a Nucleodur C18ec column (125 × 40 mm, 7 μm; Macherey-Nagel) with the following conditions: solvent A, H_2_O + 0.1% formic acid; solvent B, acetonitrile (MeCN) + 0.1% formic acid; flow, 45 mL/min; fractionation, 15 mL; gradient, isocratic conditions at 20% B for 2 min, followed by an increase to 32% B in 8 min, then an increase to 65% B in 25 min, followed by an increase to 100% B in 10 min, followed by isocratic conditions of 100% B for 10 min. This yielded the impure compound **1** (41 mg, *t*_R_ 26.0–27.1 min) and compound **2** (9 mg, *t*_R_ 19.8–20.7 min), compound **3** (11 mg, *t*_R_ 21.5–22.1 min), impure compound **4** (18 mg, *t*_R_ 24.5–25.3 min), and compound **5** (3 mg, *t*_R_ 27.4–27.8 min).

For further purification, 3.7 mg of compound **4** was given to a preparative HPLC using an Agilent 1100 series system in normal-phase condition with a Nucleosil column (250 × 10 mm, 5 μm, Macherey-Nagel, Düren, Germany) as the stationary phase and the following conditions for the mobile phase: solvent A, 90% 8 ethyl acetate: 1 petroleum ether, 10% isopropanol + 0.1% FA, solvent B, *n*-heptane + 0.1% FA; flow rate, 8 mL/min; 126 fractions were collected; gradient, isocratic step for 2 min at 100% B, followed by a decrease to 0% B in 40 min and an isocratic step at 0% B for a further 7 min. Fractions at 25.5–26 min were collected and yielded the pure compound **4** (0.62 mg).

A fraction (34 mg) containing **1** was further purified with preparative HPLC using an Agilent 1100 series system in normal-phase conditions with a Nucleosil column (250 × 21 mm, 7 μm; Macherey-Nagel, Düren, Germany) as the stationary phase and the following conditions for the mobile phase: solvent A, heptane + 0.1% formic acid (FA), solvent B, 50% ethyl acetate, 30% petroleum ether, 20% isopropanol + 0.1% FA; flow rate, 20 mL/min; 126 fractions were collected from 4 to 60 min of the following gradient, an isocratic step for 1 min at 0% B, followed by an increase to 50% B in 60 min, and a further increase to 100% in 9 min. The fraction from 38.6 to 39.1 min was collected and provided pure **1** (2 mg).

#### Botryosulfuranol D (**1**)

White amorphous powder; [α]_D_^20^ −328 (1.5 mg/mL, MeOH); UV (MeOH) λ_max_ (log ε) 275.5 (3. 7), 202 (4.5) nm; CD {MeOH, λ [nm] (Δε), *c* = 0.22 × 10^–3^ M}: 191 (+27), 204 (−16.5), 216 (−10.6), 245 (−28.5), 303 (+1); ^1^H NMR (700 MHz, CHCl_3_-*d*) see Table 1; ^13^C NMR (175 MHz, CHCl_3_-*d*) see Table 1; HRESI-MS *m*/*z* 449.0468 [M + H]^+^ (calcd for C_19_H_17_N_2_O_7_S_2_, 449.0472).

#### Botryosulfuranol E (**2**)

White amorphous powder, slightly yellowish; [α]_D_^20^ −149 (1.3 mg/mL, MeOH); UV (MeOH) λ_max_ (log ε) 276.5 (3.6), 202 (4.5) nm; CD {MeOH, λ [nm] (Δε), *c* = 0.43 × 10^–3^ M}: 202 (−15.6), 221 (−8.7), 243 (−11.0); ^1^H NMR (700 MHz, CHCl_3_-*d*) see Table 1; ^13^C NMR (175 MHz, CHCl_3_-*d*) see Table 1; HRESI-MS *m*/*z* 467.0575 [M + H]^+^ (calcd for C_19_H_19_N_2_O_8_S_2_, 467.0577), *m*/*z* 489.0395 [M + Na]^+^ (calcd for C_19_H_18_N_2_NaO_8_S_2_, 489.0397).

#### Botryosulfuranol F (**3**)

White amorphous powder; [α]_D_^20^ −176 (1.0 mg/mL, MeOH);); UV (MeOH) λ_max_ (log ε) 279 (3.6), 202.5 (4.5) nm; CD {MeOH, λ [nm] (Δε), *c* = 0.40 × 10^–3^ M}: 204 (+33), 231 (−29); ^1^H NMR (700 MHz, acetone-*d*_6_) see Table 1; ^13^C NMR (175 MHz, acetone-*d*_6_) see Table 1; HRESI-MS *m*/*z* 499.0296 [M + H]^+^ ]^+^ (calcd for C_19_H_19_N_2_O_8_S_3_, 499.00298), *m*/*z* 521.0115 [M + Na]^+^ (calcd for C_19_H_18_N_2_NaO_8_S_3_, 521.0117), *m*/*z* 997.0521 [2M + H]^+^ (calcd for C_38_H_37_N_4_O_16_S_6_, 997.0523).

#### Botryosulfuranol G (**4**)

White amorphous powder; [α]_D_^20^ −9 (0.5 mg/mL, MeOH); UV (MeOH) λ_max_ (log ε) 279 (3.4), 202.5 (4.5) nm; CD {MeOH, λ [nm] (Δε), *c* = 0.42 × 10^–3^ M}: 195 (+8.2), 219 (−10.9), 279 (+2.8), 298 (0.0), 333 (0.5); ^1^H NMR (700 MHz, CHCl_3_-*d*) see Table 1; ^13^C NMR (175 MHz, CHCl_3_-*d*) see Table 1; HRESI-MS *m*/*z* 479.0941 [M + H]^+^ (calcd for C_21_H_23_N_2_O_7_S_2_, 479.0941).

#### Botryosulfuranol A (**5**)

Yellow oil, CD {MeOH, λ [nm] (Δε), *c* = 0.65 × 10^–3^ M}: 207 (−2.7), 212 (−2.6), 221 (−3.2), 358 (−0.1) nm; ^1^H NMR (700 MHz, CHCl_3_-*d*) see Table 1; ^13^C NMR (175 MHz, CHCl_3_-*d*) see Table 1; HRESI-MS *m*/*z* 463.0990 [M + H]^+^ (calcd for C_21_H_23_N_2_O_6_S_2_, 463.0992).

### Derivatization with MTPA

Compound **1** (7 mg) was dissolved in dry pyridine, and 3.5 mg of (*R*)-(−)-α-methoxy-α-(trifluoromethyl) phenylacetyl chloride (10 μL) was added. The reaction was incubated for 17.5 h at room temperature. The resulting (*S*)-MTPA ester was purified using an Agilent 1200 Infinity Series HPLC UV system (Agilent Technologies) with a XBridge column (250 × 10 mm, 5 μm, Waters GmbH) and the following gradient: solvent A, water + 0.1% FA; solvent B, MeCN + 0.1% FA; flow rate 5 mL/min. This resulted in 0.5 mg of the pure (*S*)-MTPA ester derivative of **1**. ^1^H NMR (700 MHz, CHCl_3_-*d*): similar to **1** but δ_H_ 6.92 (dd, *J* = 10.4, 2.7 Hz, H-7), 6.15 (dd, *J* = 10.4, 1.3 Hz, H-8), 6.07 (m, H-6), 5.79 (s, H-3), 2.85 (s, NCH_3_), 2.80 (dd, *J* = 14.6, 5.4 Hz, H_a_-5); 2.40 (dd, *J* = 14.6, 7.3 Hz, H_b_-5). A 0.37 mg amount of the (*R*)-MTPA ester derivative was yielded analogously by treatment with (*S*)-(+)-methoxy-(trifluoromethyl) phenylacetyl chloride. ^1^H NMR (700 MHz, CHCl_3_-*d*): similar to **1** but δ_H_ 6.61 (dd, *J* = 10.4, 1.4 Hz, H-7), 5.95 (m, H-6), 5.78 (dd, *J* = 10.4, 1.8 Hz, H-8), 5.58 (s, H-3), 2.95 (s, NCH_3_), 2.66 (dd, *J* = 14.4, 5.6 Hz, H_a_-5); 2.06 (dd, *J* = 14.4, 8.0 Hz, H_b_-5).

### Biological Assays

*Morinagamyces vermicularis* CBS 303.81 was cultivated in a 500 mL conical flask containing 200 mL of YM, ZM, or Q6 for 15, 9, or 12 days, respectively. The mycelium was separated from the supernatant by filtration through a funnel with filter paper. Crude extracts were obtained by extraction of the mycelium with acetone for 30 min at 40 °C in an ultrasonic bath. The acetone phase was dried until the water phase. The obtained water phase was extracted with ethyl acetate. The supernatant was extracted with ethyl acetate. Obtained crude extracts were tested against *Bacillus subtilis* DSM10, *Candida tenuis* MUCL29892, and *Mucor plumbeus* MUCL49355 in a serial dilution assay with test concentrations 300, 150, 75, 38, 19, 9, 5, and 2 μg/mL.

The antimicrobial activities of the isolated compounds were evaluated by determining the minimum inhibitory concentration of 50 (MIC50) against five fungi (i.e., *Candida albicans*, *Mucor hiemalis*, *Rhodotorula glutinis*, *Schizosaccharomyces pombe*, and *Wickerhamomyces anomalus*) and against different Gram-positive (*Bacillus subtilis*, *Mycolicibacterium smegmatis*, and *Staphylococcus aureus*) and Gram-negative (*Acinetobacter baumannii*, *Chromobacterium violaceum*, *Escherichia coli*, and *Pseudomonas aeruginosa*) bacteria using nystatin as a positive control against all of the tested fungi and oxytetracycline against all of the bacteria, except for *A. baumannii*, *My. smegmatis*, and *Ps. aeruginosa*, against which ciprobay, kanamycin, and gentamycin were used, respectively. The cytotoxicity of compounds **1**–**3** and **5** was tested against seven mammalian cell lines, i.e., human endocervical adenocarcinoma KB 3.1, breast cancer MCF-7, lung cancer A549, ovary cancer SK-OV-3, prostate cancer PC-3, squamous cancer A431, and mouse fibroblasts L929, and was determined by the MTT method using epothilone B as the positive control. Both biological assays were performed following the protocols described by Harms and colleagues.^5^

## Supporting Information Available

Candidates from ChemWalker analysis; NMR and MS data and ECD spectra of compounds **1**–**5**; and NMR data and NMR spectra of compounds **7** and **8** (PDF)
