# Supplementary material for: Reaping the Chemical Diversity of Morinagamyces vermicularis Using Feature-Based Molecular Networking
Source: J Nat Prod. 2024 Sep 16;87(9):2335–42. doi: 10.1021/acs.jnatprod.4c00654 (PMC11443486; doi:10.1021/acs.jnatprod.4c00654)
Supplement: Supplementary file 1 — np4c00654_si_001.pdf [file np4c00654_si_001.pdf]

Supplementary material:

Reaping the chemical diversity of *Morinagamyces vermicularis* using feature-based molecular networking

Karen Harms[1, 2,‡], Esteban Charria-Girón[1,2, ‡], Alberdto Miguel Stchigel[3], Yasmina Marin-Felix[1,2], Frank Surup[1,2]**

[1]Department Microbial Drugs, Helmholtz Centre for Infection Research, Inhoffenstraße 7, 38124 Braunschweig

[2]Institute of Microbiology, Technische Universität Braunschweig, Spielmannstraße 7, 38106 Braunschweig, Germany

[3]Mycology Unit, Medical School and IISPV, Universitat Rovira i Virgili, C/ Sant Llorenç 21, Tarragona, 43201 Reus, Spain

Table S1. Top five candidate structures for botryosulfuranol A and C resulting from the ChemWalker analysis. The $[M+Na]^+$ (https://gnps2.org/status?task=1837a7f29de34c9fa128aba758daae7d) and $[M+H]^+$ (https://gnps2.org/status?task=6061da91b2f84faca625e6b98dc4073b) adducts were used for botryosulfuranol A and C respectively.

Botryosulfuranol A (Cluster index = 378)		
**Candidate**	**Score**	**Structure**
1	1.0	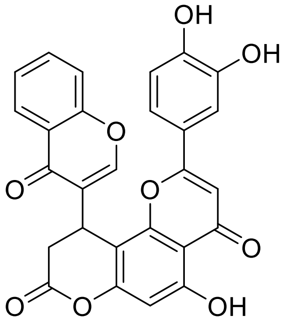
2	0.896063	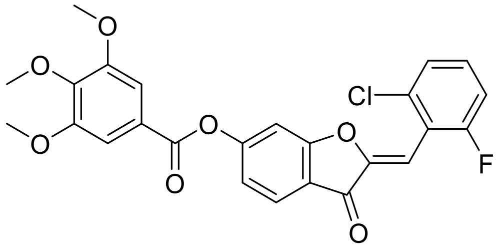
3	0.746944	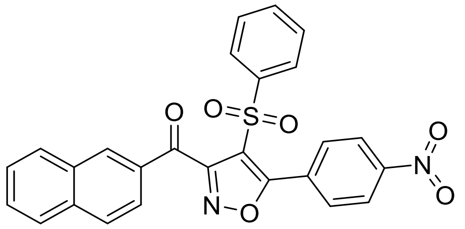
4	0.746944	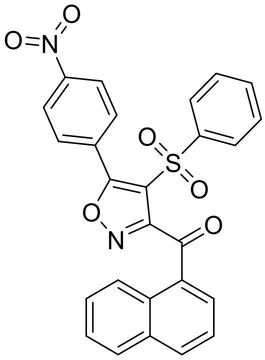
5	0.277381	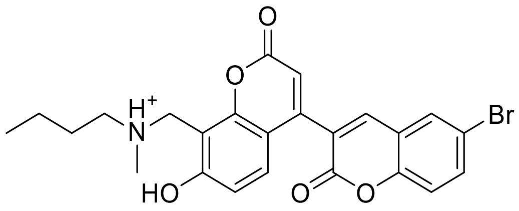

Botryosulfuranol C (Cluster index = 76)		
**Candidate**	**Score**	**Structure**
1	1.0	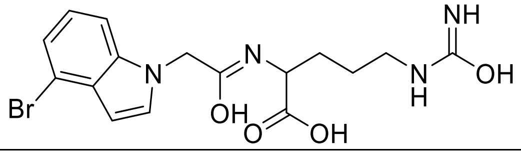
2	0.896821	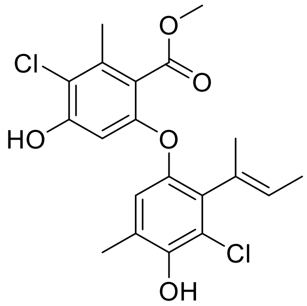
3	0.880618	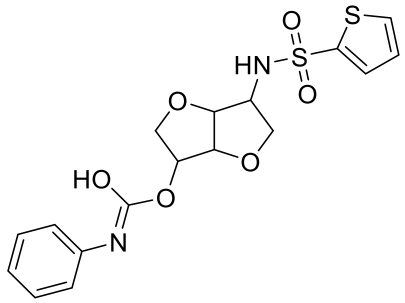
4	0.814627	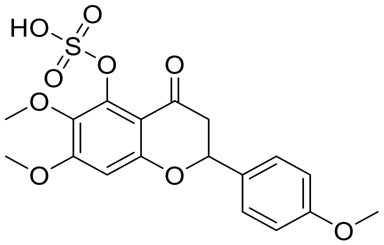
5	0.795508	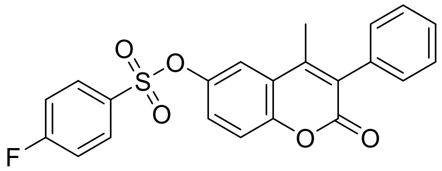

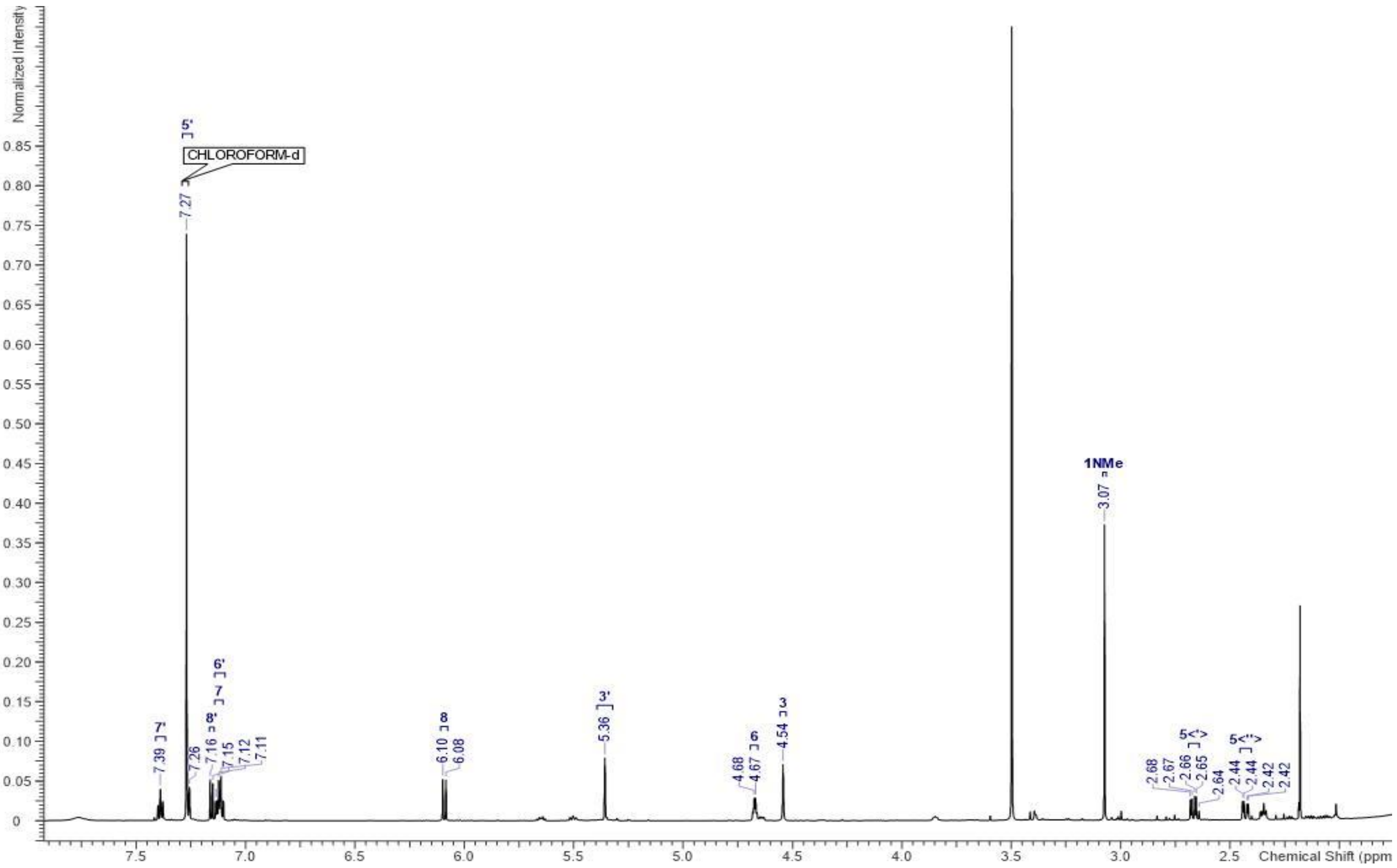

Figure S1. ^{1}H NMR spectrum of **1** in $CDCl_3$ (700 MHz).

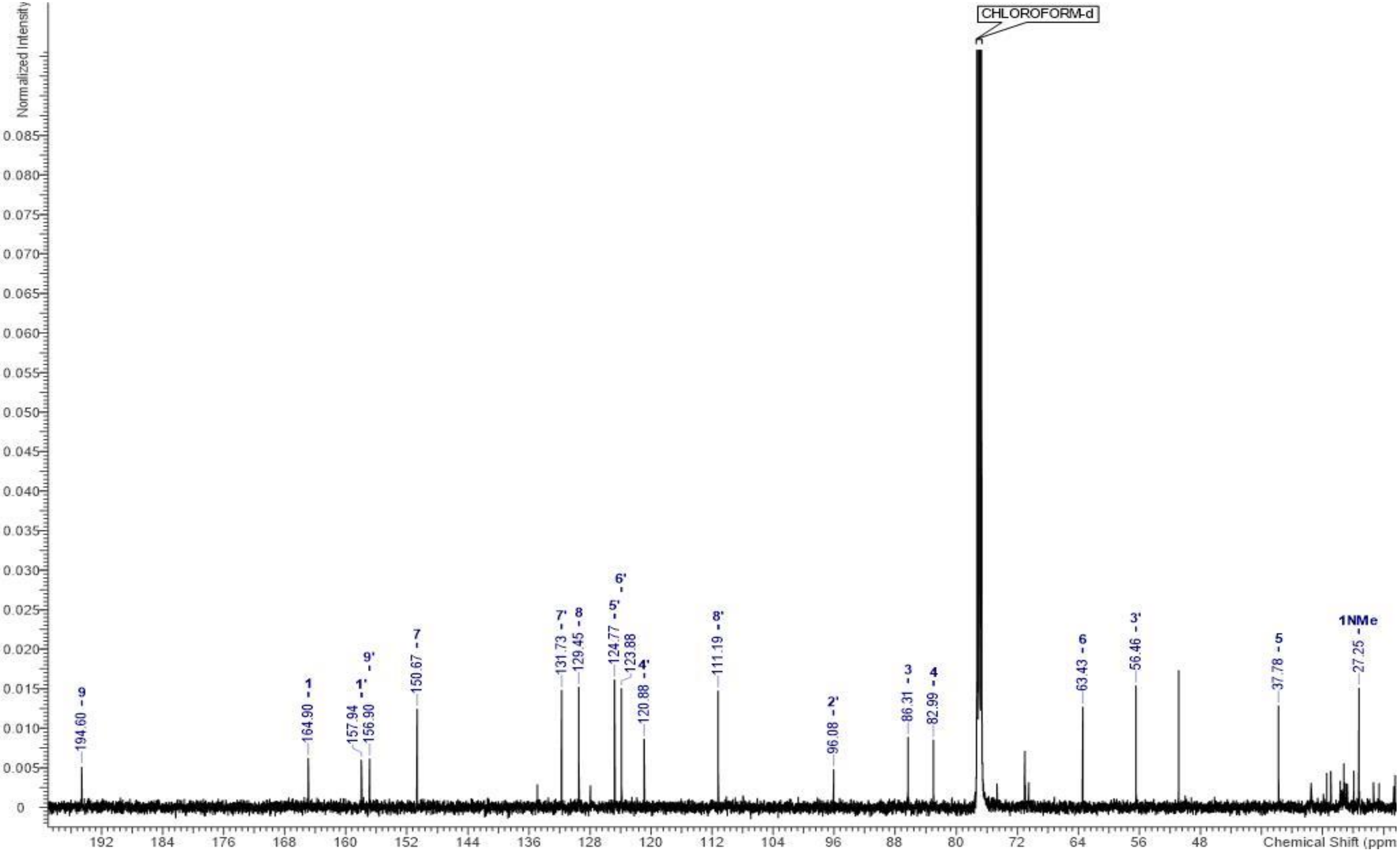

Figure S2. ^{13}C NMR spectrum of **1** in $CDCl_3$ (700 MHz).

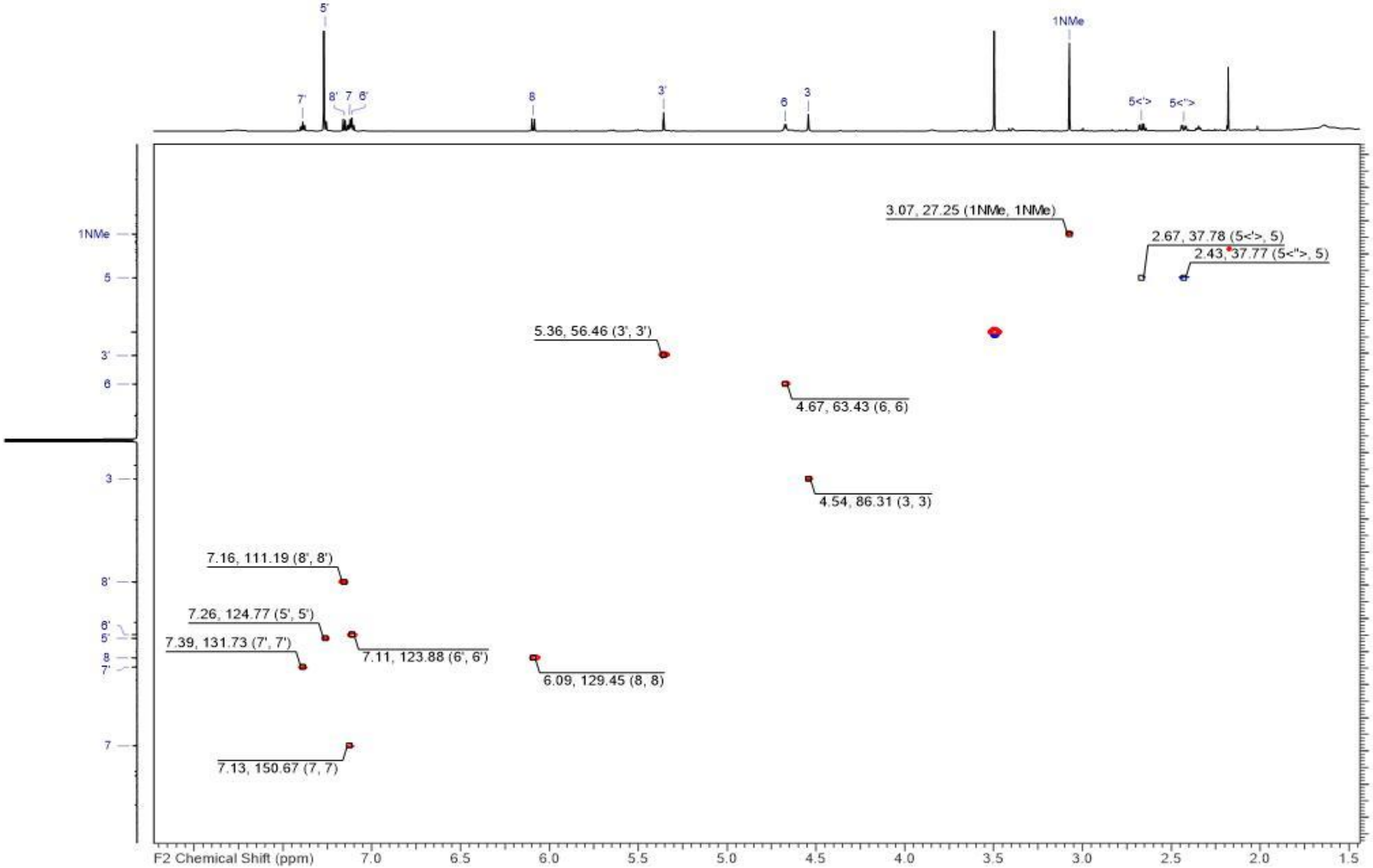

Figure S3. HSQC-DEPT NMR spectrum of **1** in $CDCl_3$ (700 MHz).

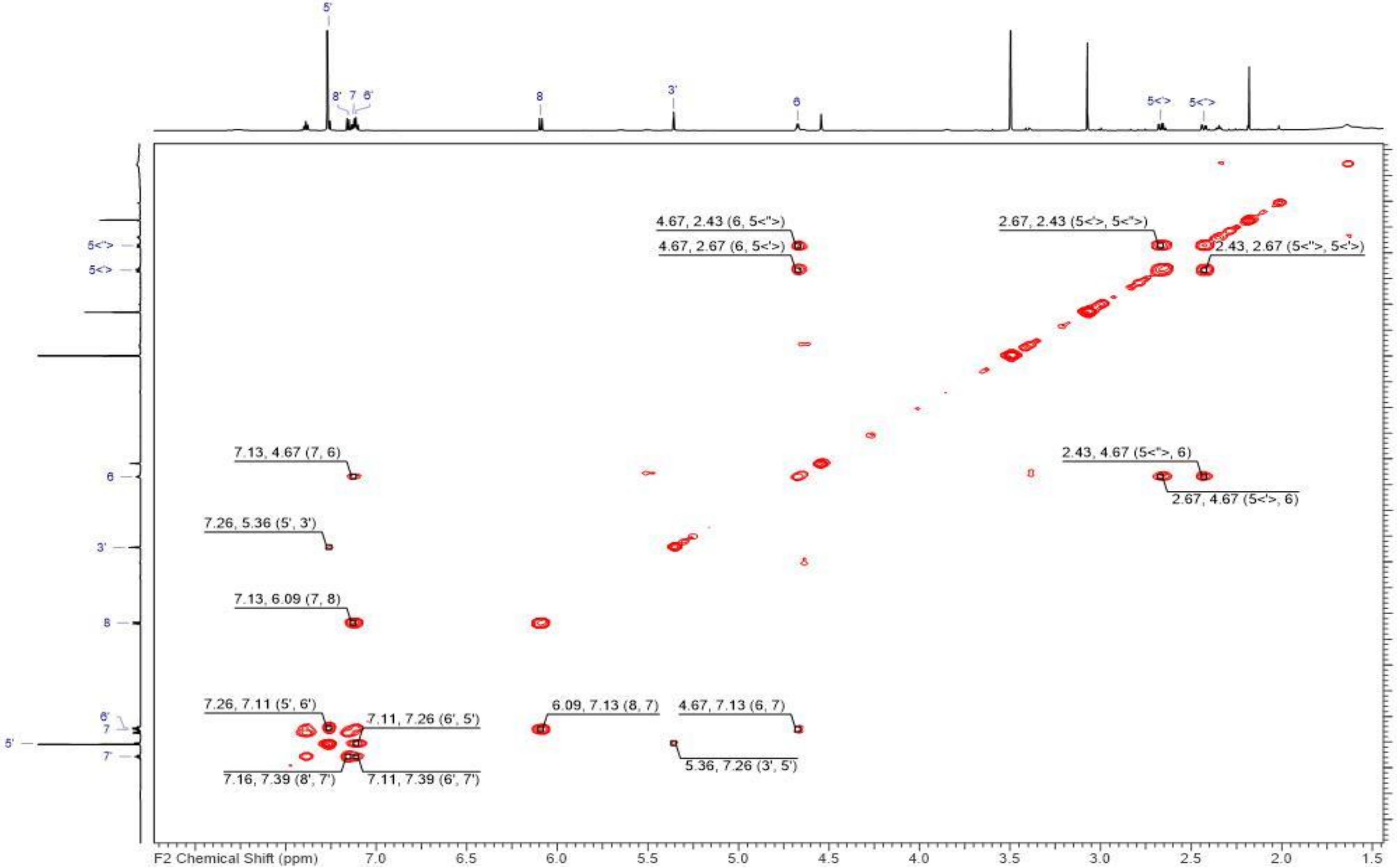

Figure S4. COSY NMR spectrum of **1** in $CDCl_3$ (700 MHz).

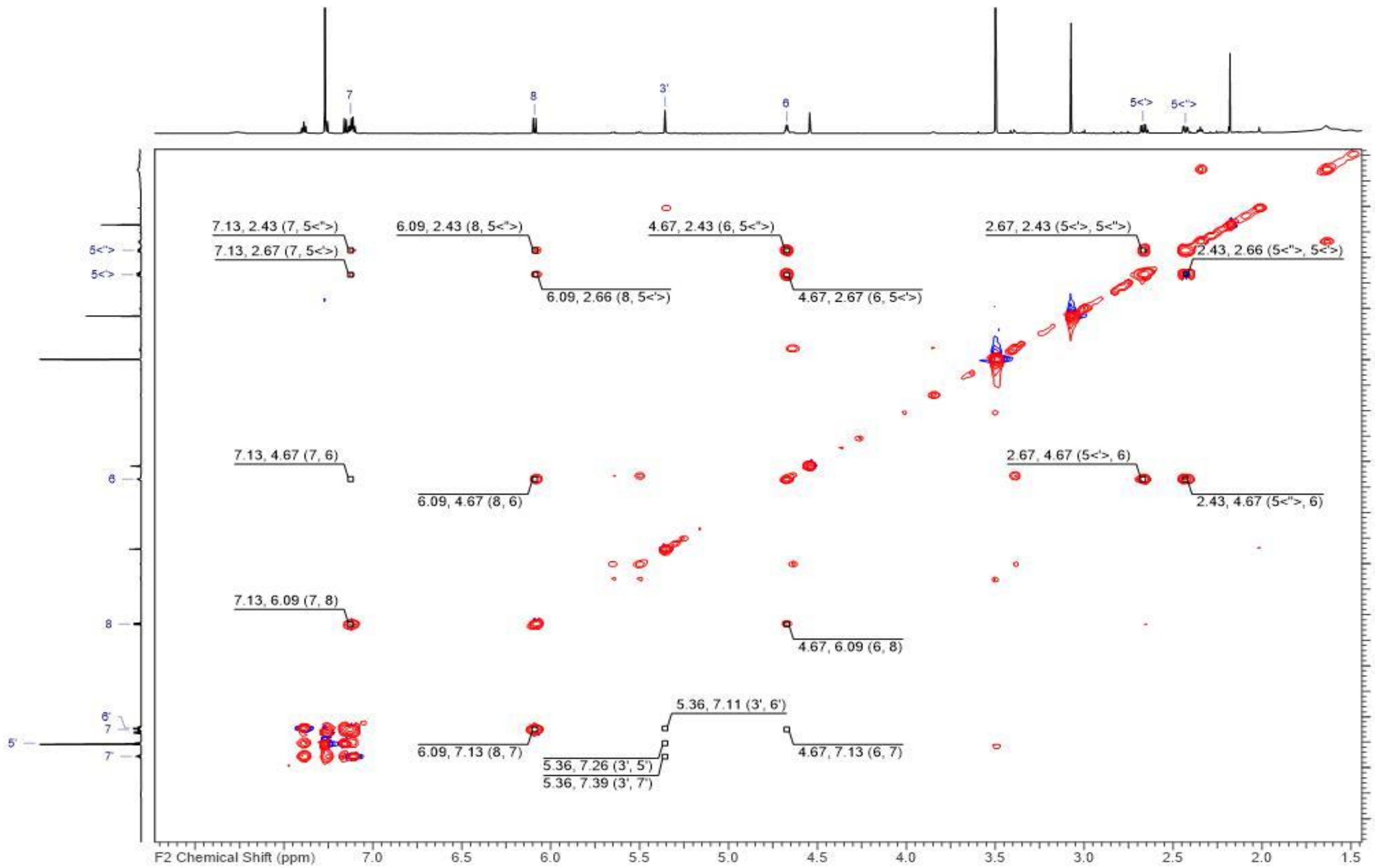

Figure S5. TOCSY NMR spectrum of **1** in $CDCl_3$ (700 MHz).

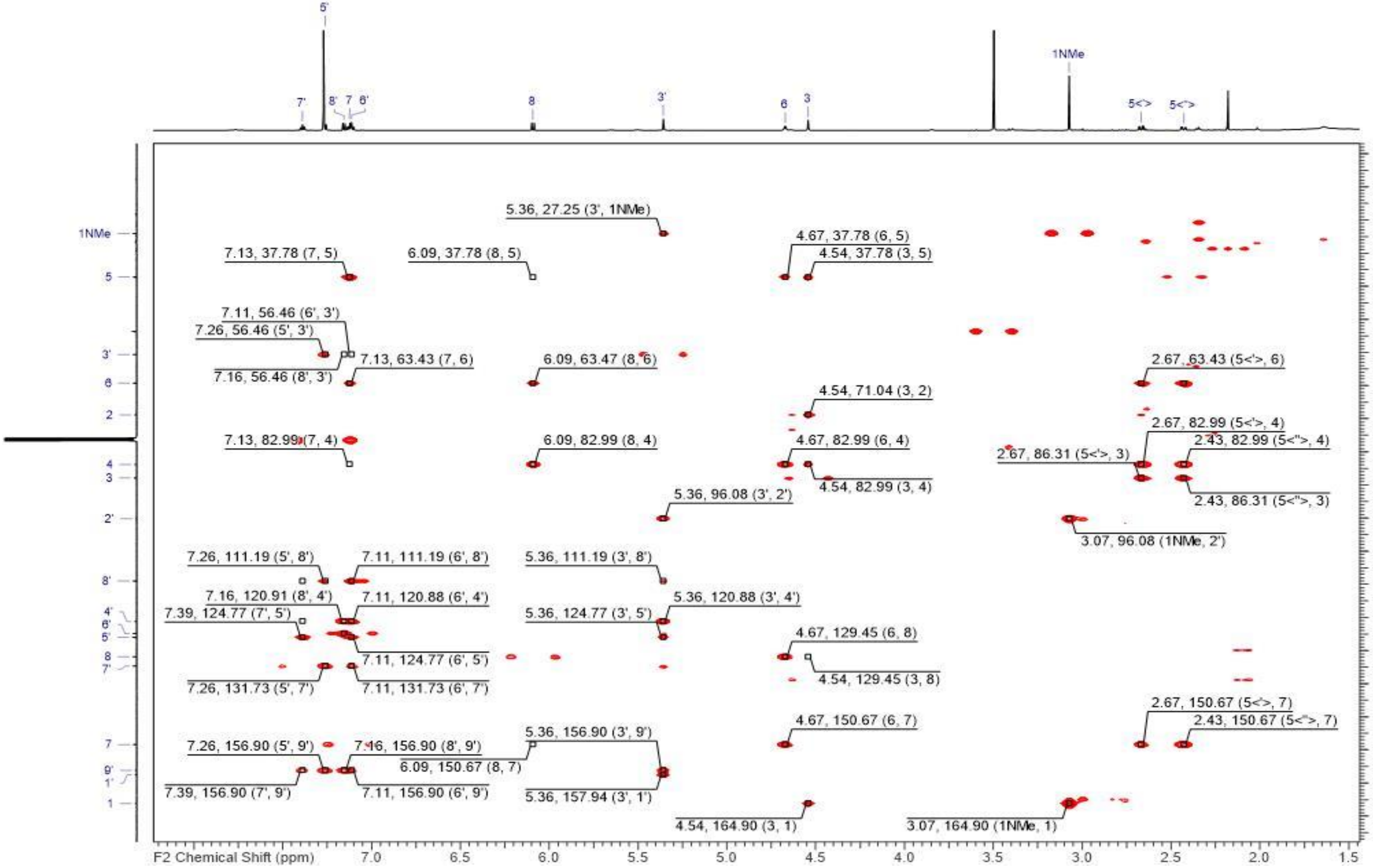

Figure S6. HMBC NMR spectrum of **1** in $CDCl_3$ (700 MHz).

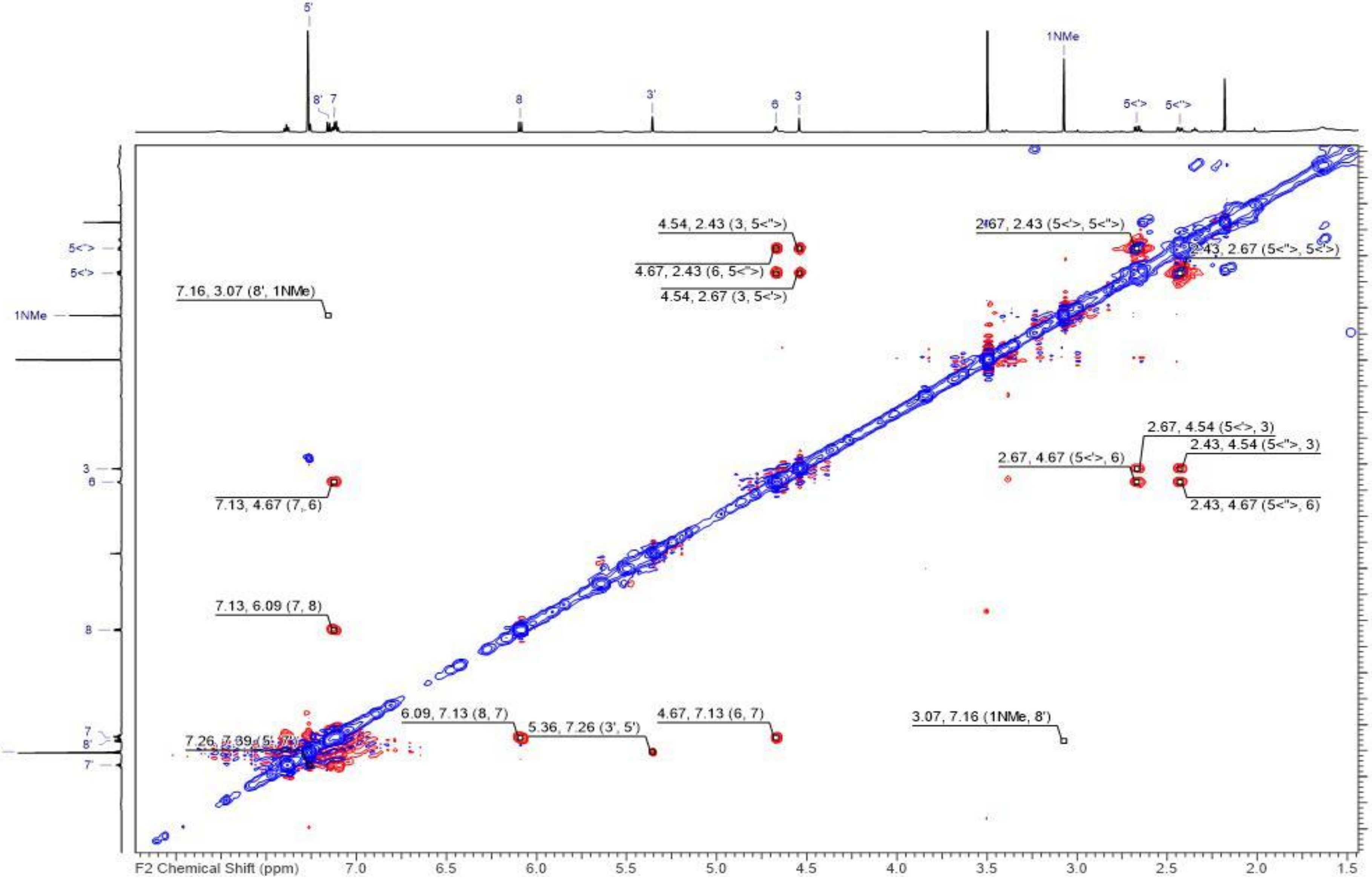

Figure S7. ROESY NMR spectrum of **1** in $CDCl_3$ (700 MHz).

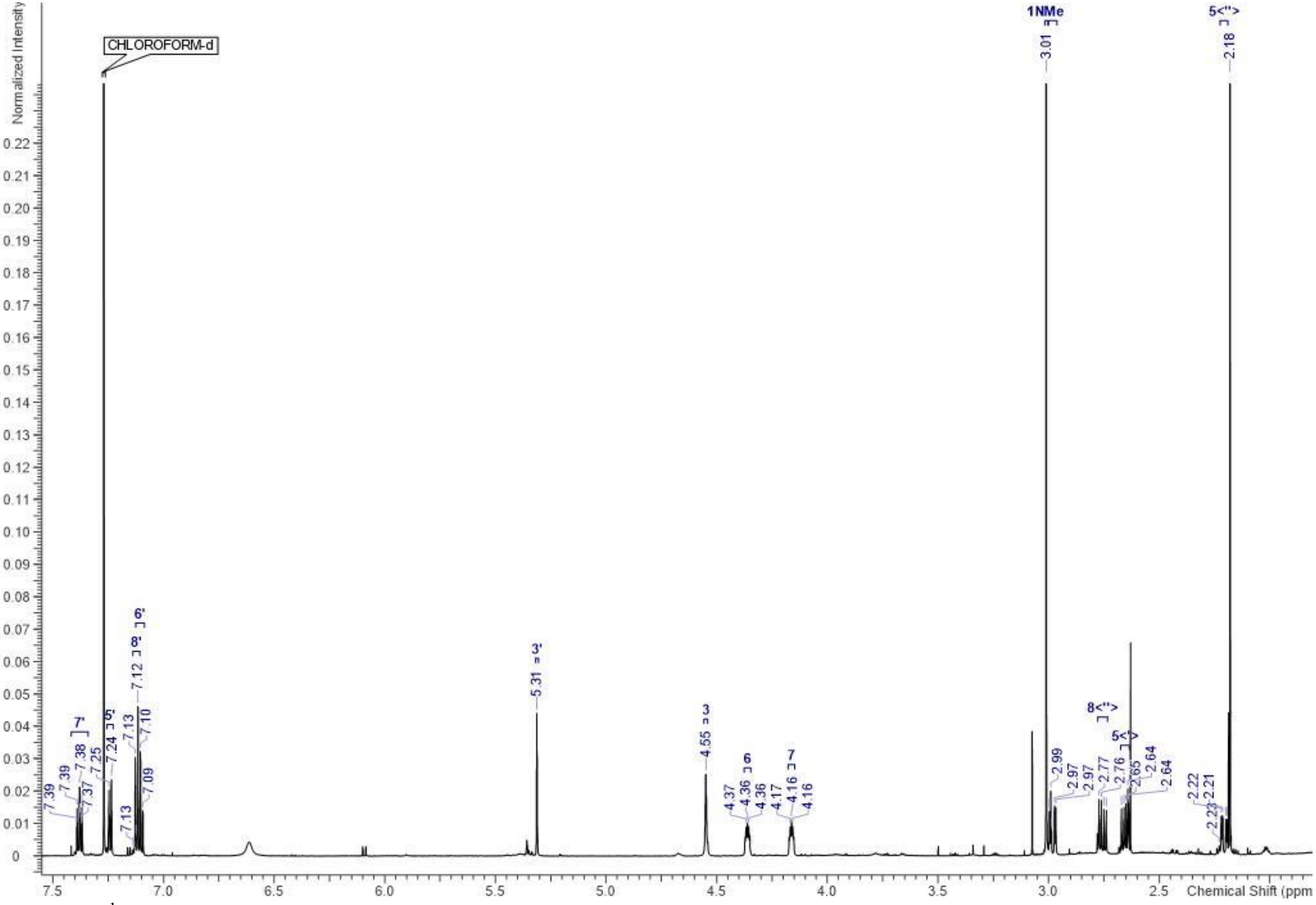

Figure S8. ^{1}H NMR spectrum of **2** in $CDCl_3$ (700 MHz).

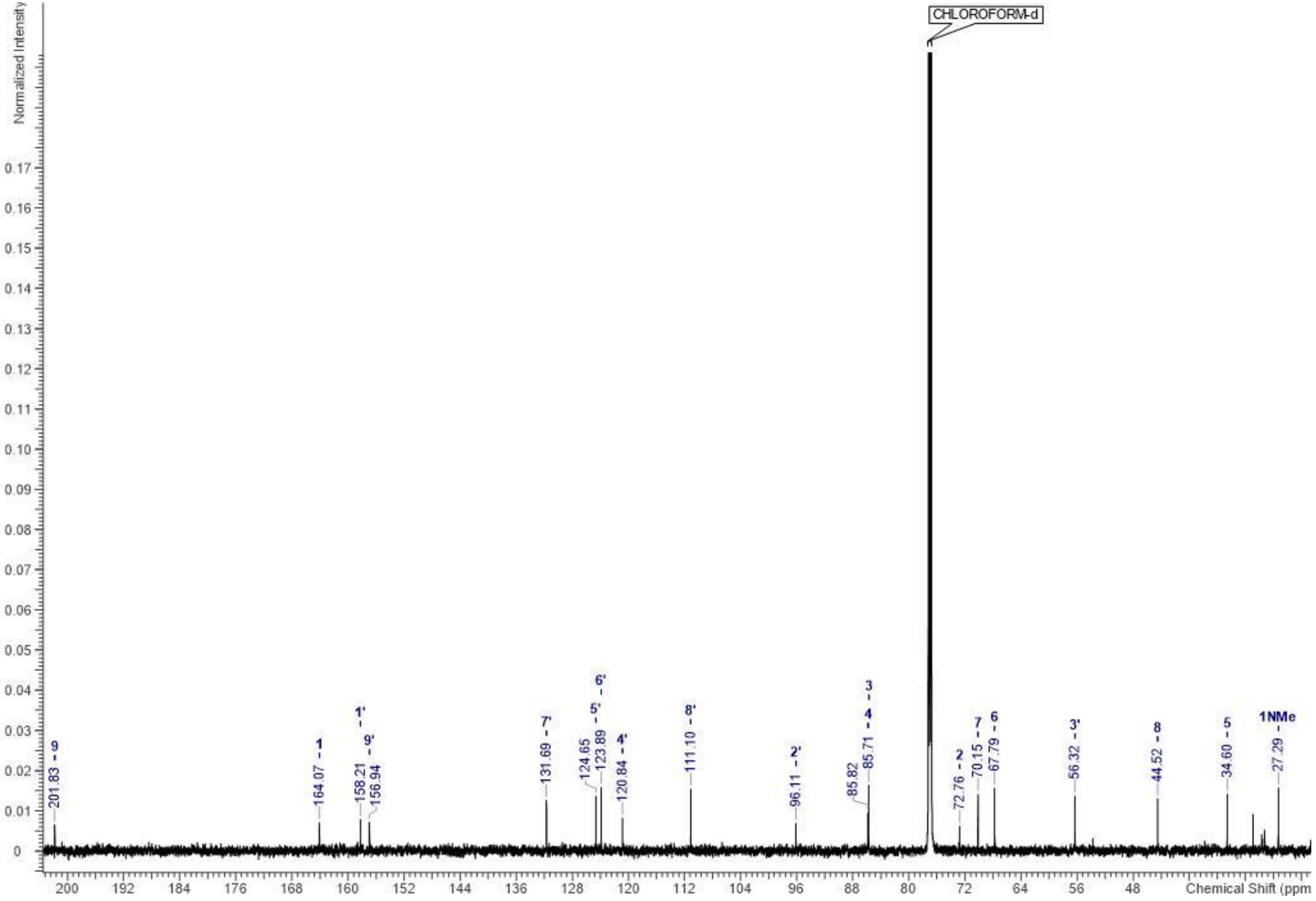

Figure S9. ^{13}C NMR spectrum of **2** in $CDCl_3$ (700 MHz).

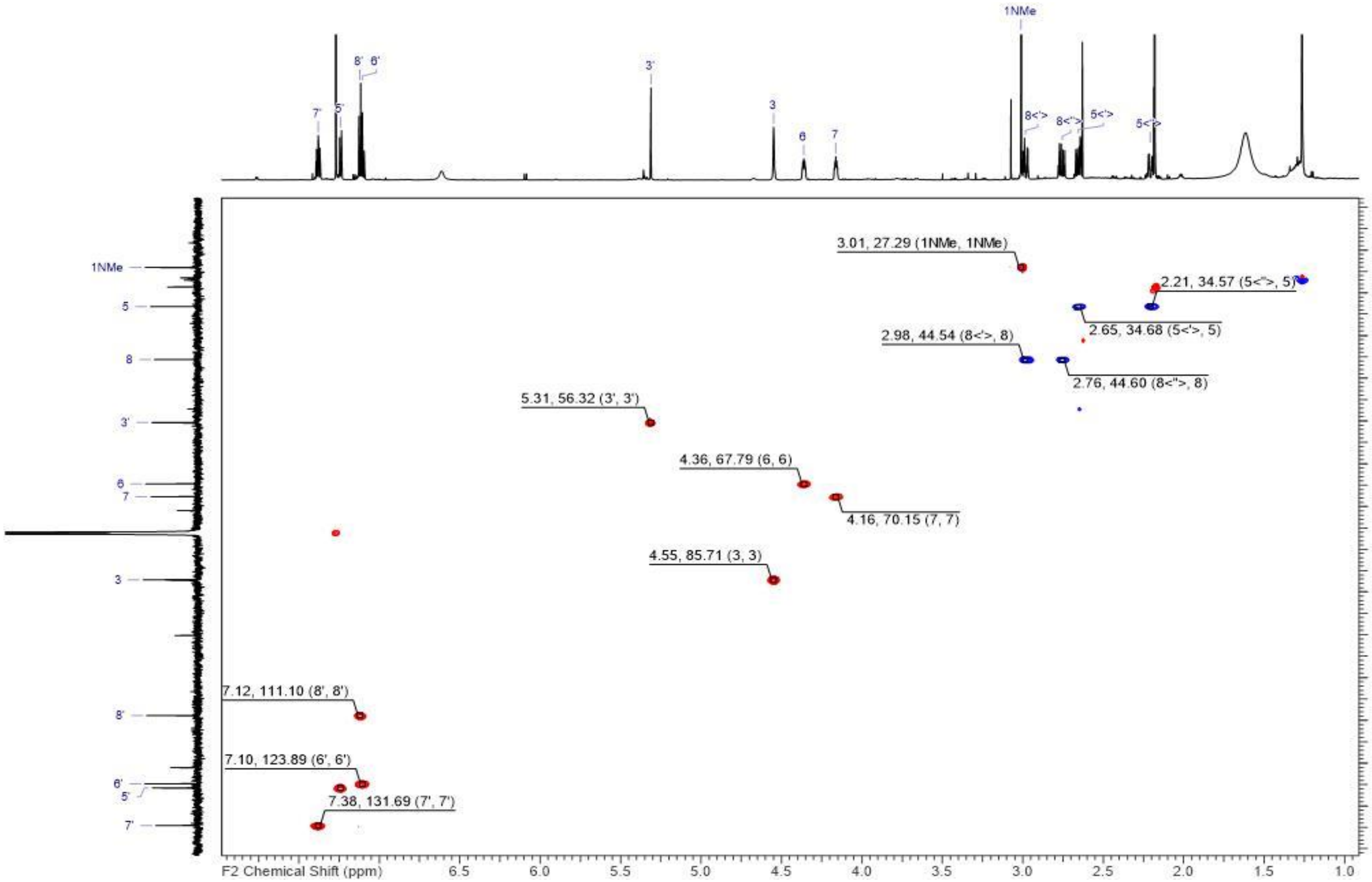

Figure S10. HSQC NMR spectrum of **2** in $CDCl_3$ (700 MHz).

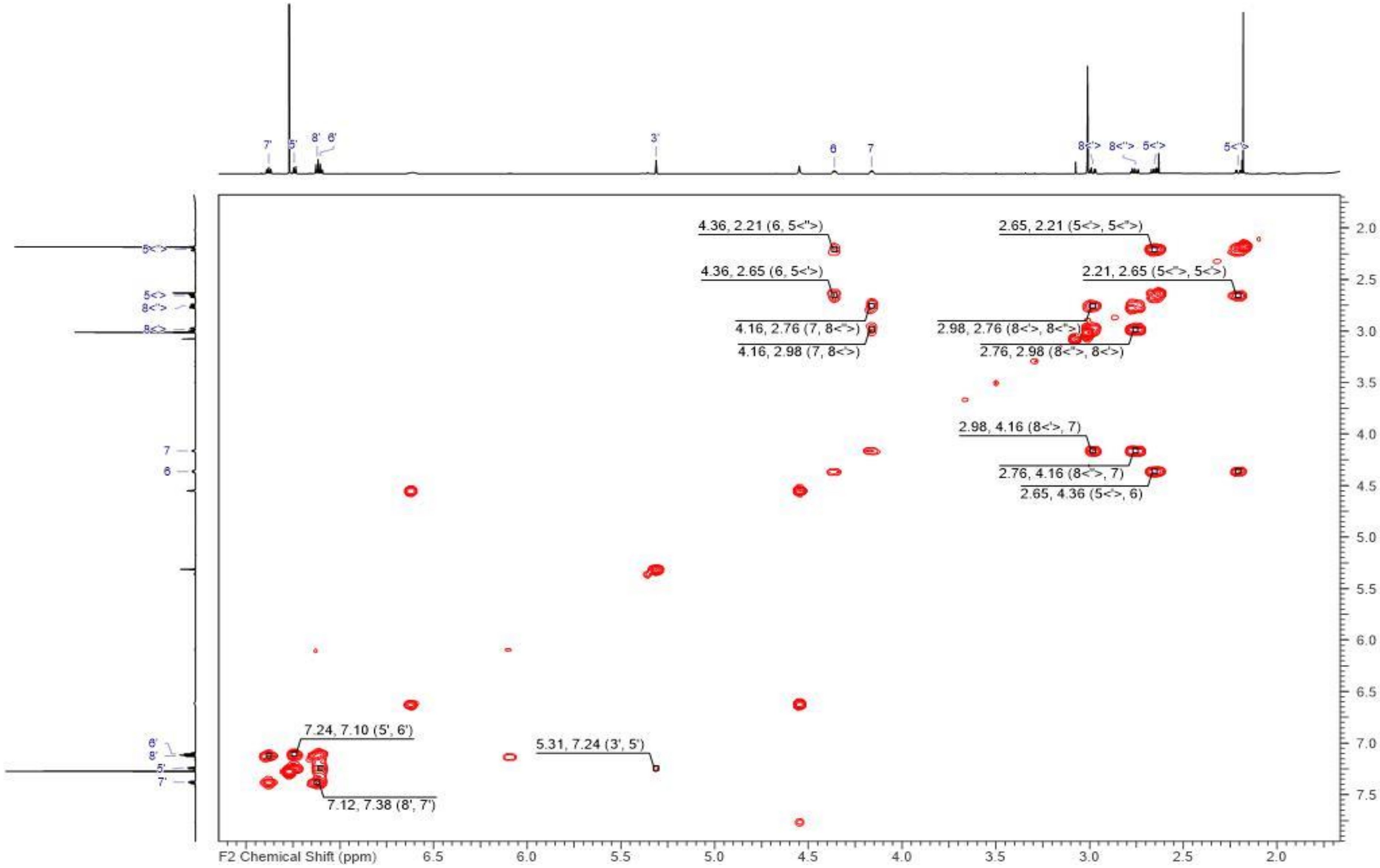

Figure S11. COSY NMR spectrum of **2** in $CDCl_3$ (700 MHz).

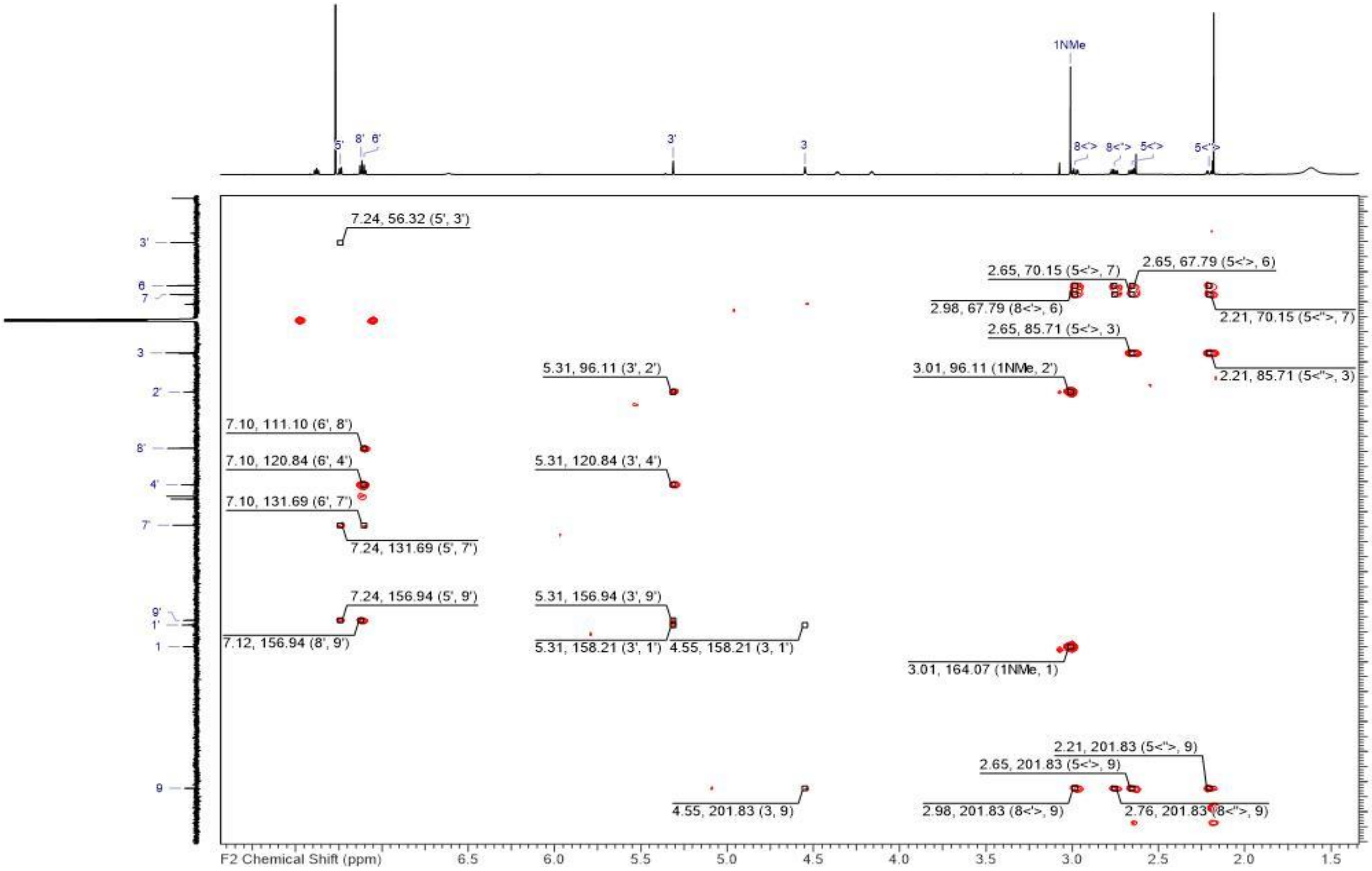

Figure S12. HMBC NMR spectrum of **2** in $CDCl_3$ (700 MHz).

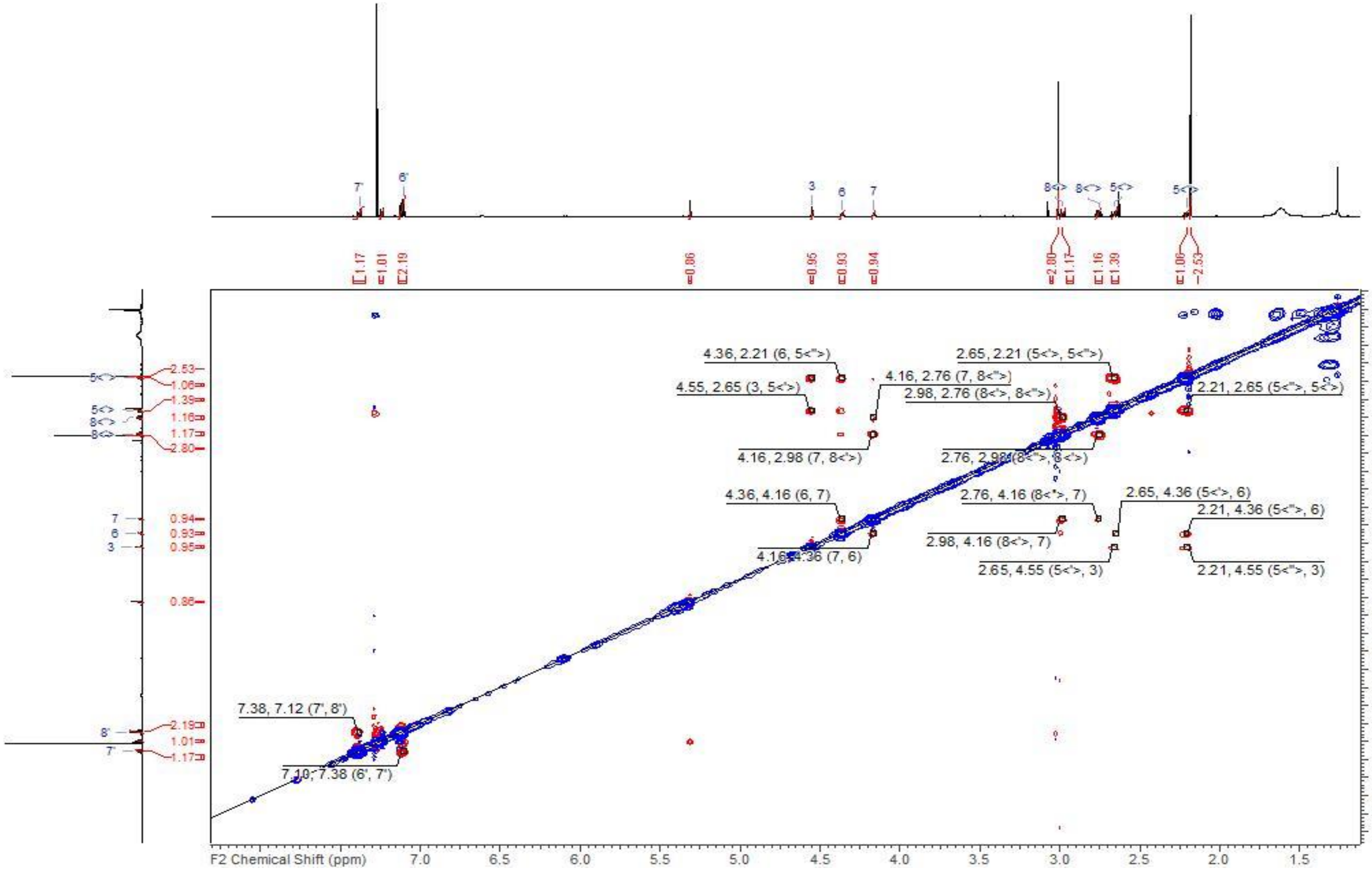

Figure S13. ROESY NMR spectrum of **2** in $CDCl_3$ (700 MHz).

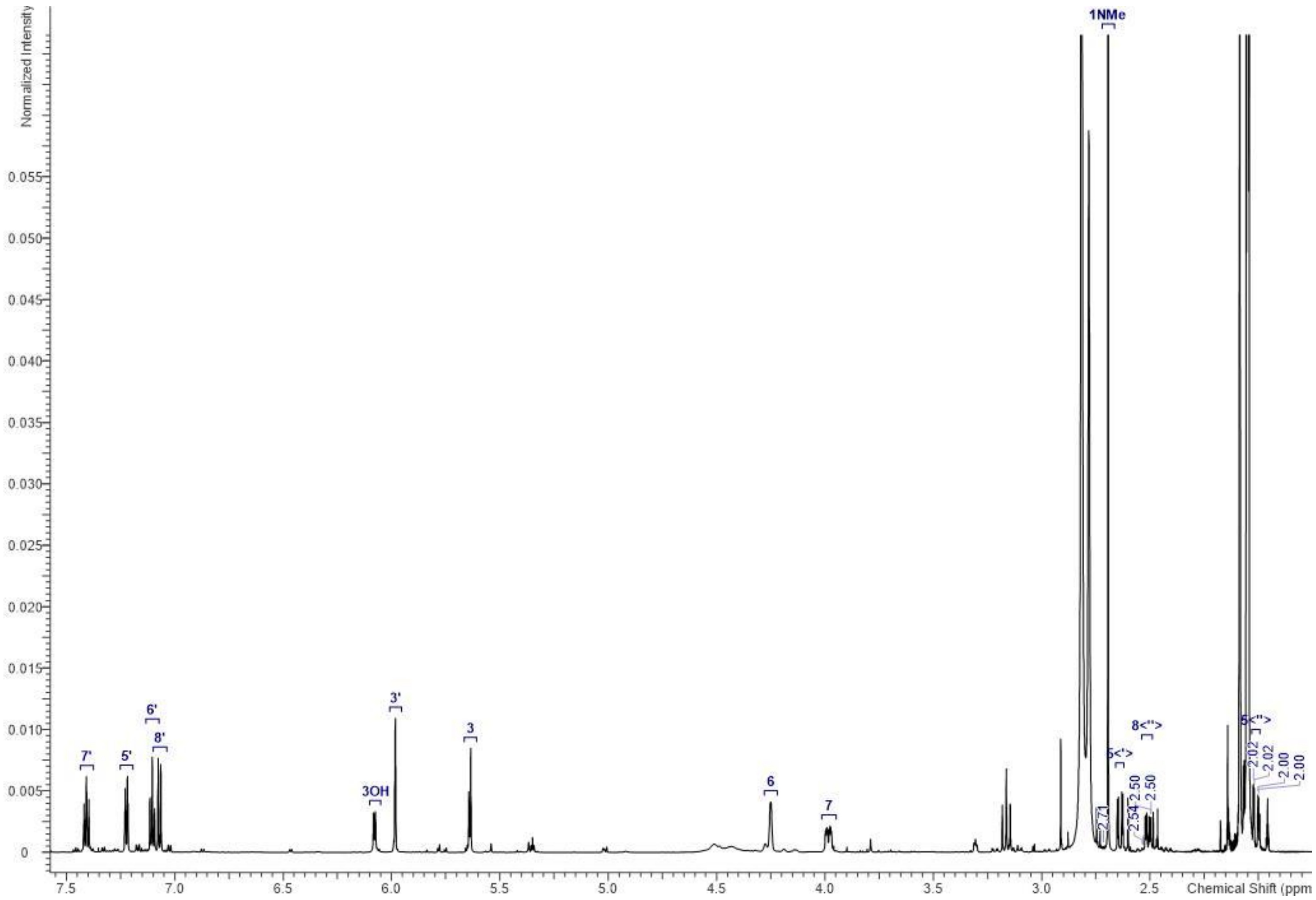

Figure S14. ^{1}H NMR spectrum of **3** in $(CD_3)_2CO$ (700 MHz).

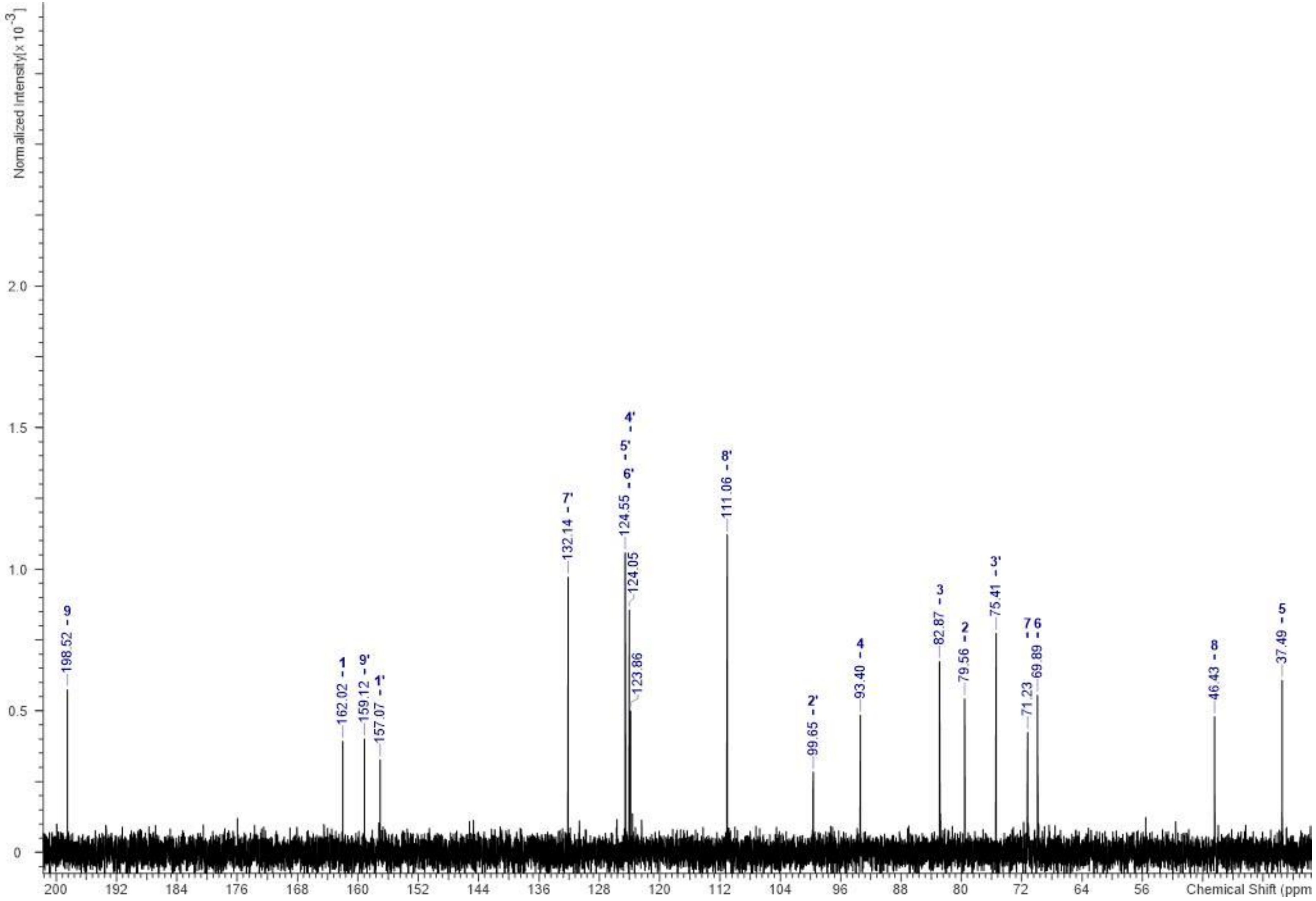

Figure S15. ^{13}C NMR spectrum of **3** in $(CD_3)_2CO$ (700 MHz).

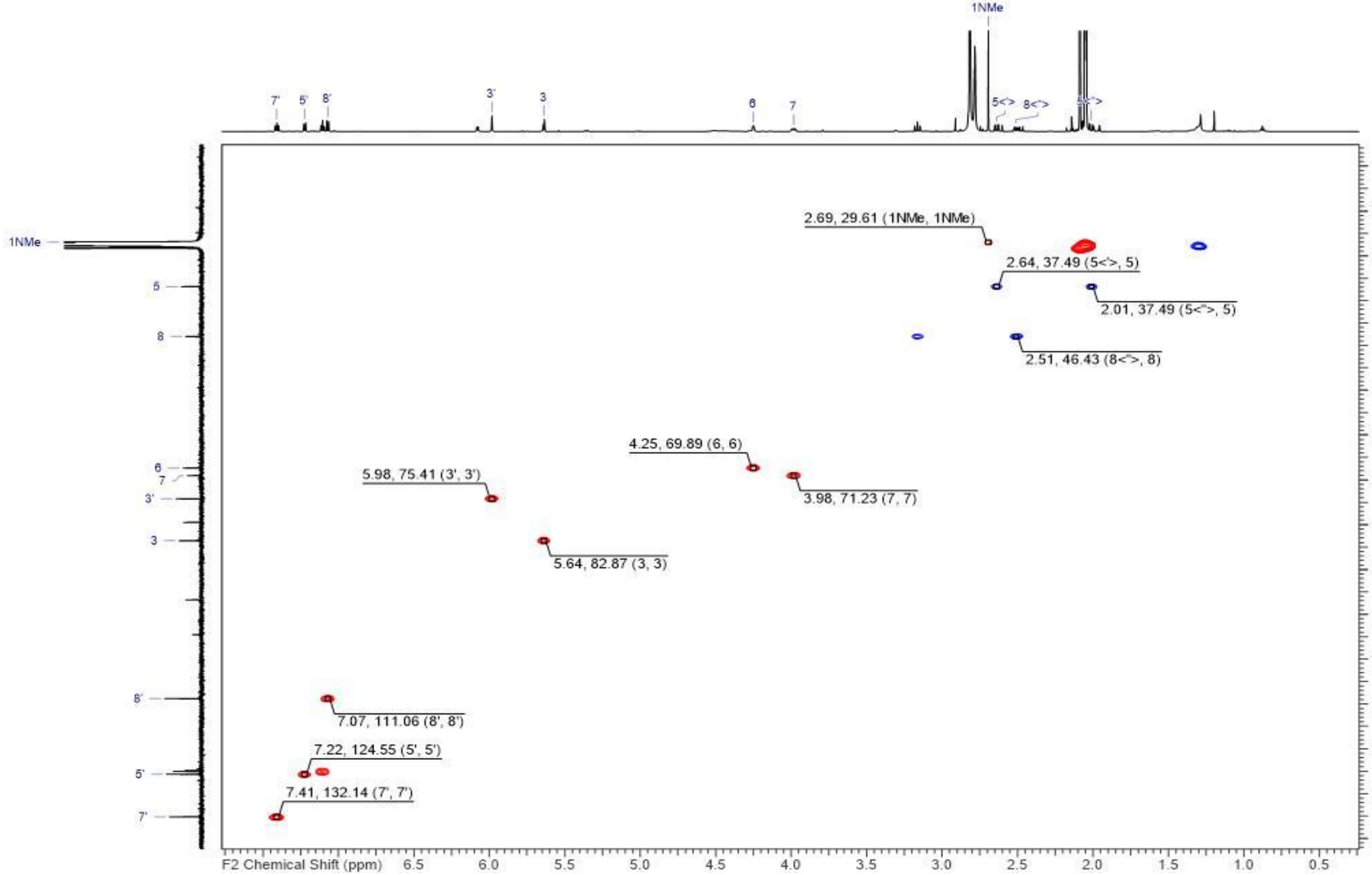

Figure S16. HSQC-DEPT NMR spectrum of **3** in $(CD_3)_2CO$ (700 MHz).

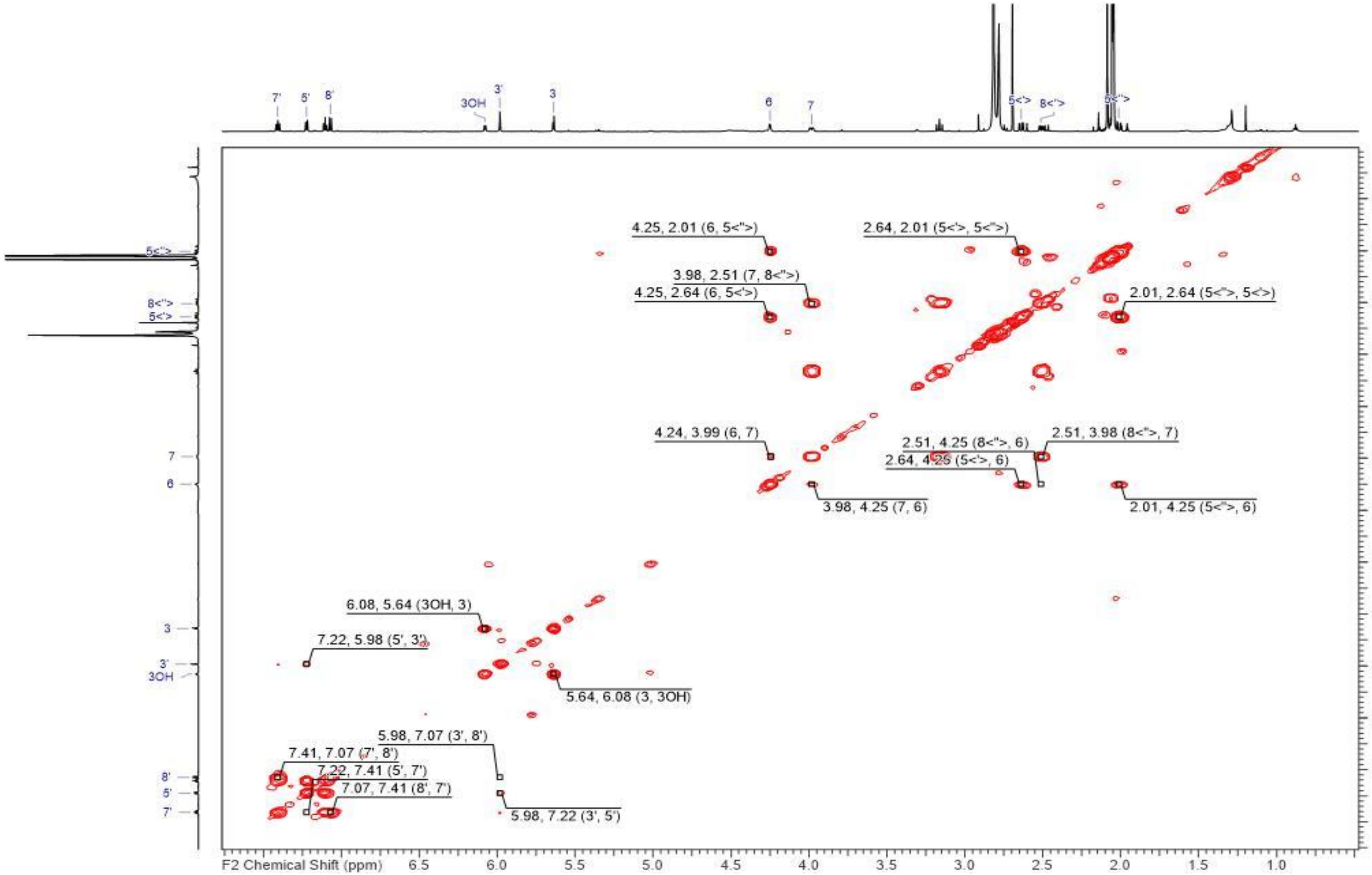

Figure S17. COSY NMR spectrum of **3** in $(CD_3)_2CO$ (700 MHz).

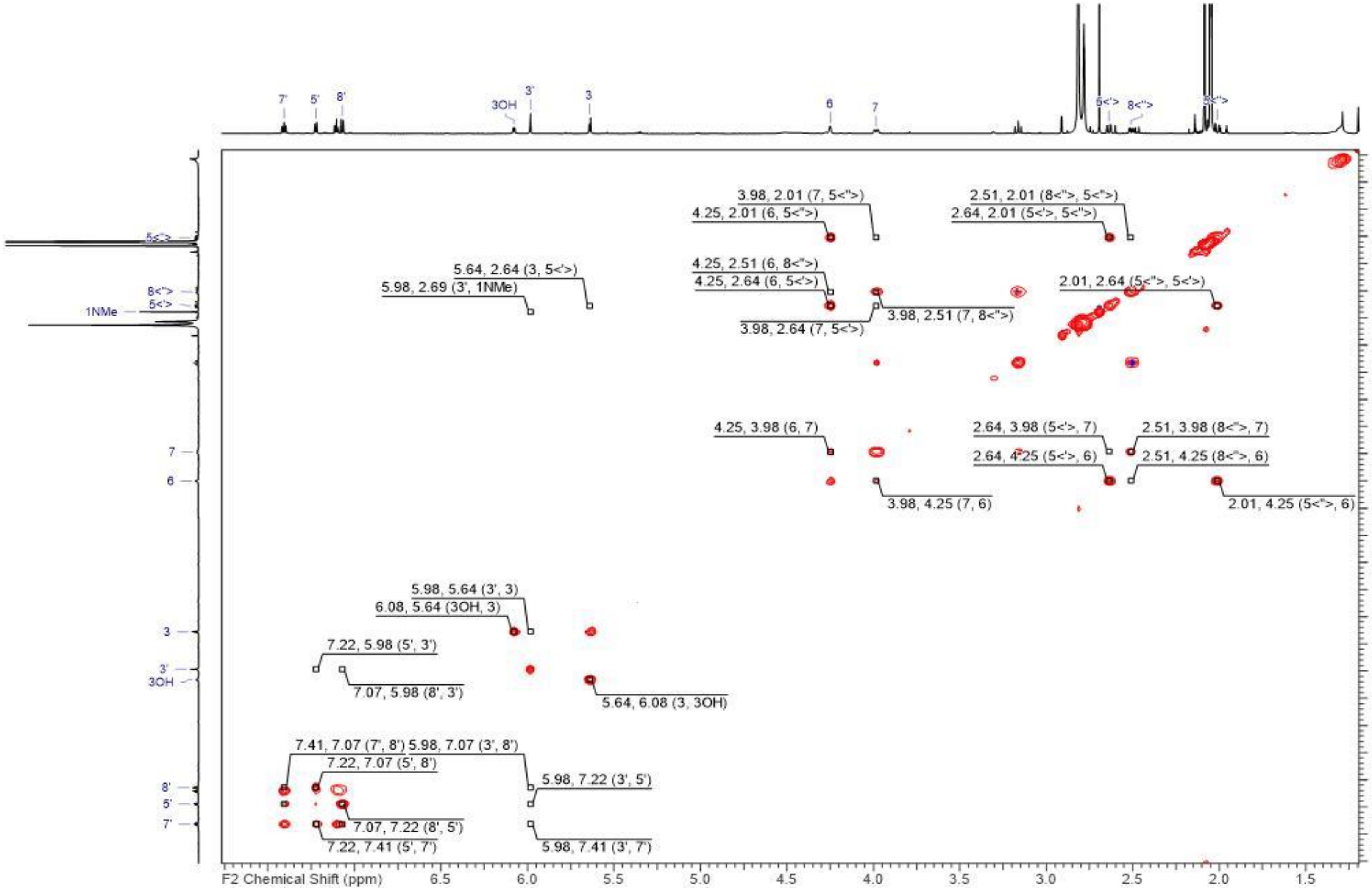

Figure S18. TOCSY NMR spectrum of **3** in $(CD_3)_2CO$ (700 MHz).

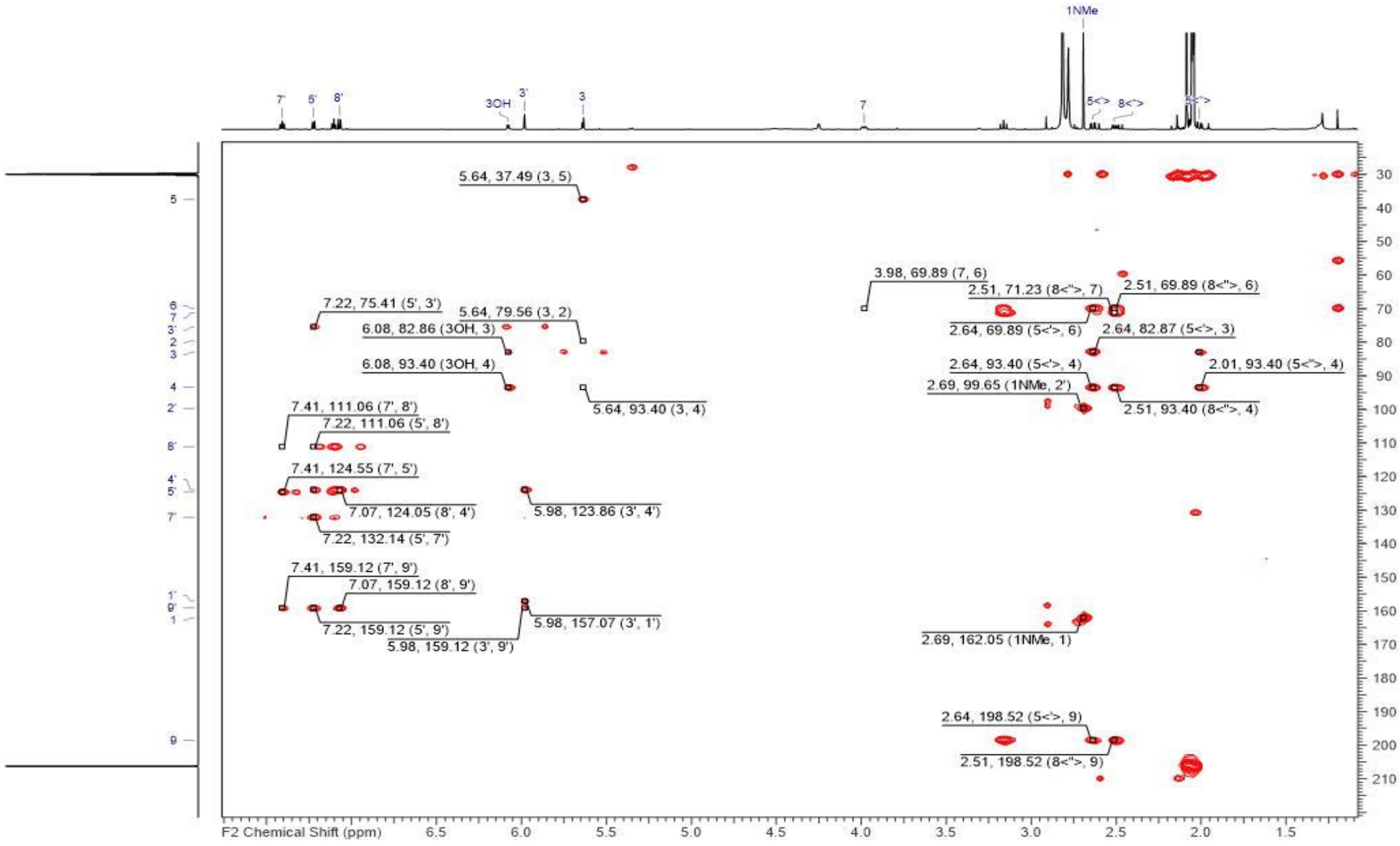

Figure S19. HMBC NMR spectrum of **3** in $(CD_3)_2CO$ (700 MHz).

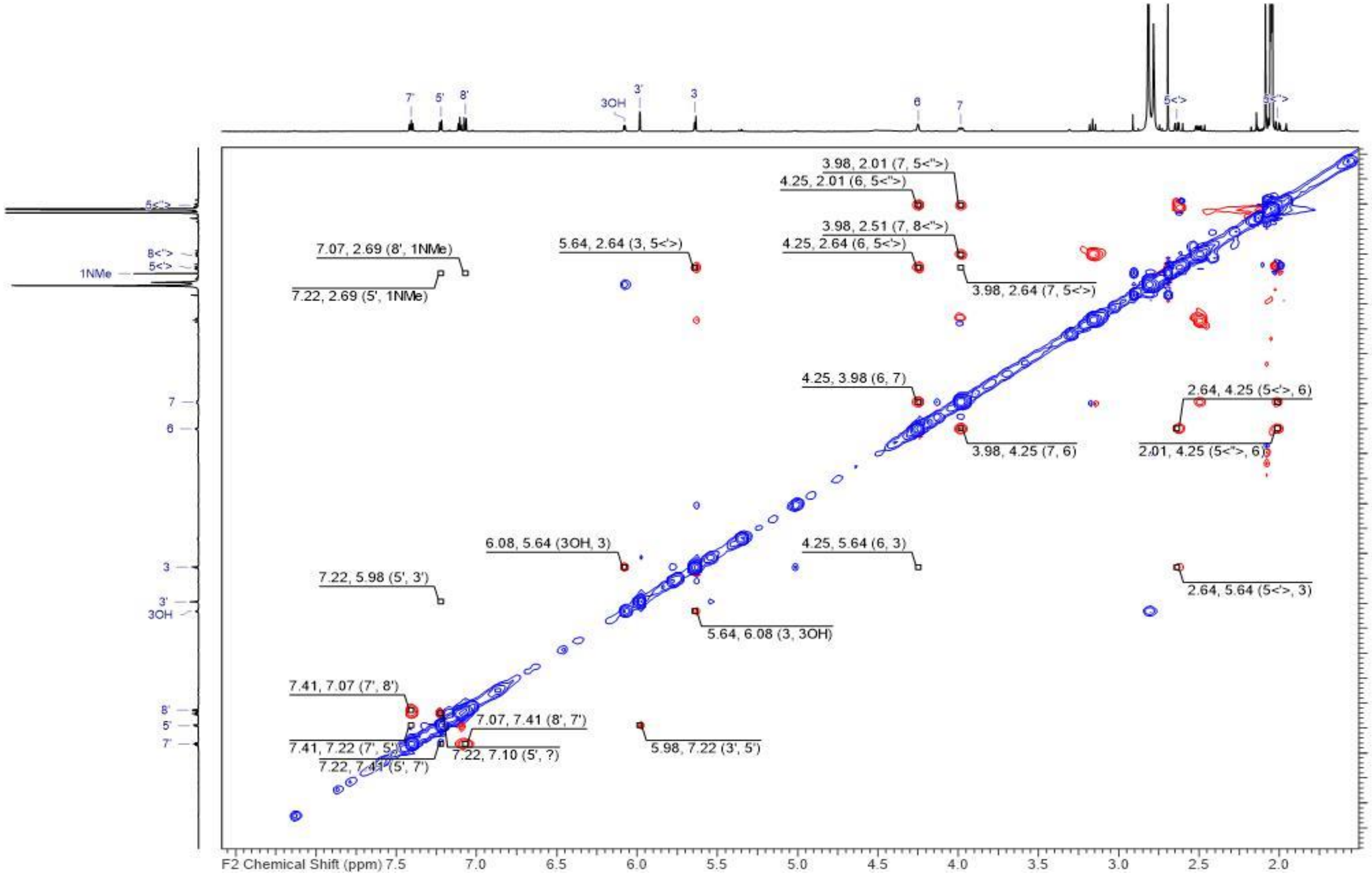

Figure S20. ROESY NMR spectrum of **3** in $(CD_3)_2CO$ (700 MHz).

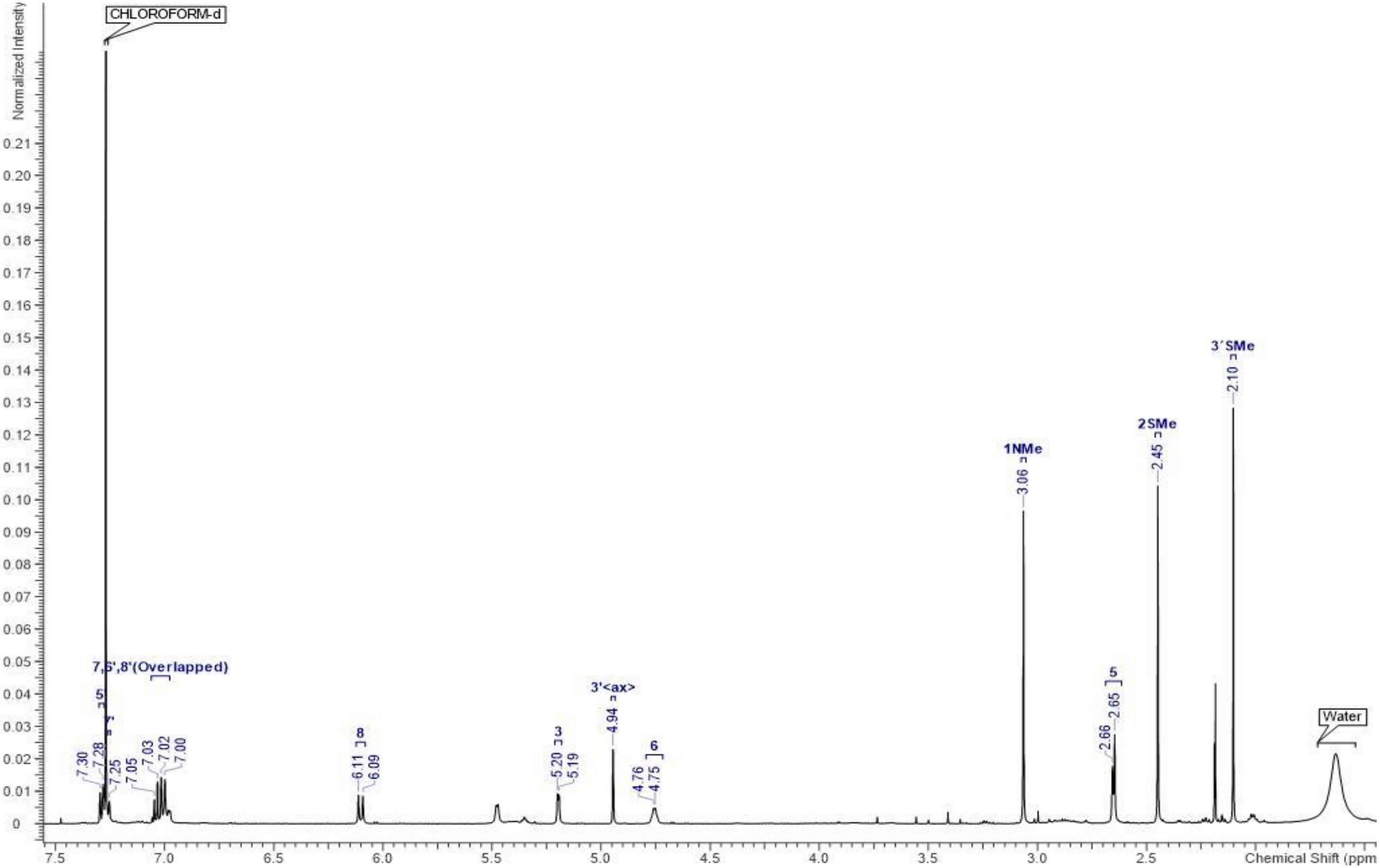

Figure S21. ^{1}H NMR spectrum of **4** in $CDCl_3$ (500 MHz).

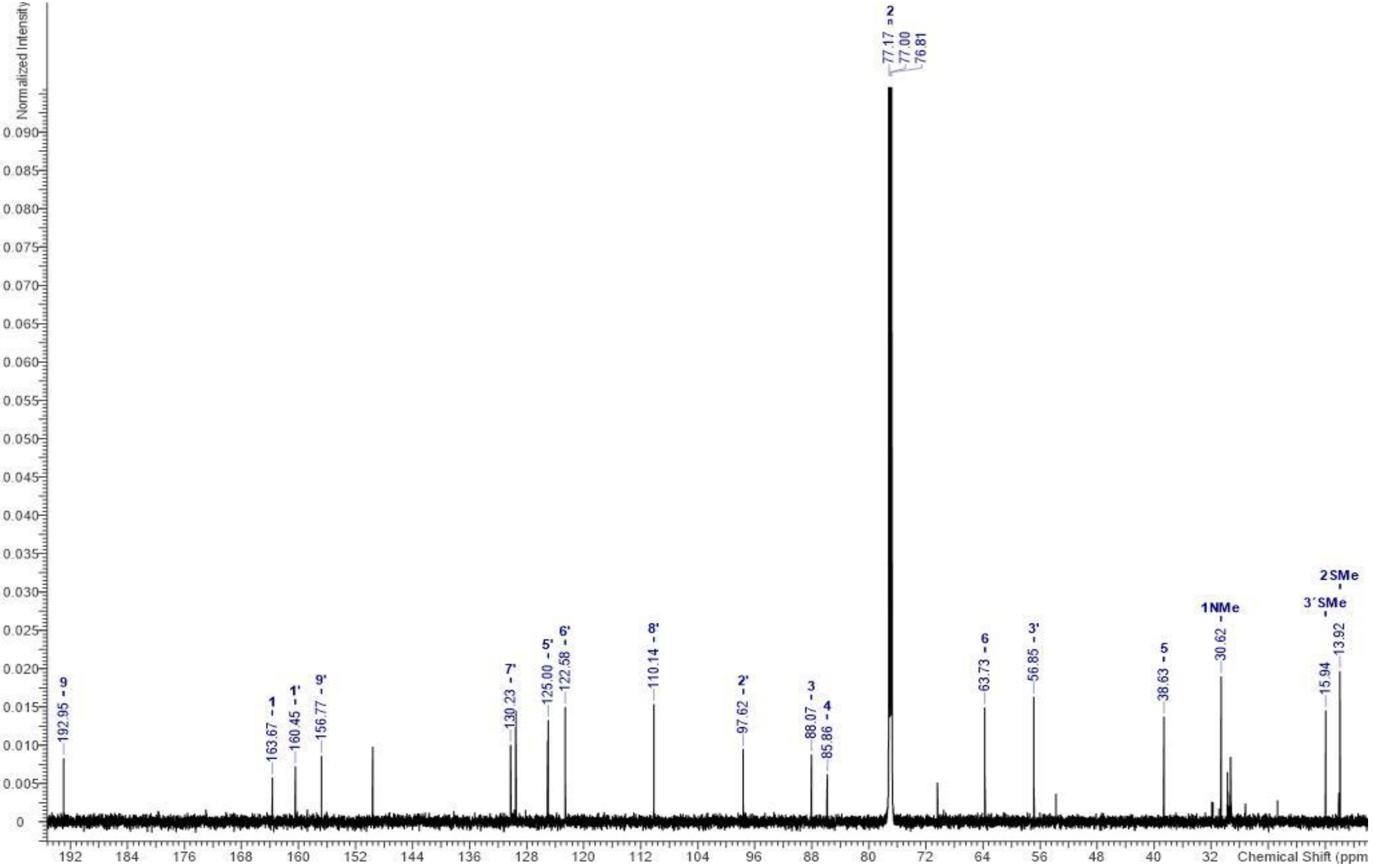

Figure S22. ^{13}C NMR spectrum of **4** in $CDCl_3$ (500 MHz).

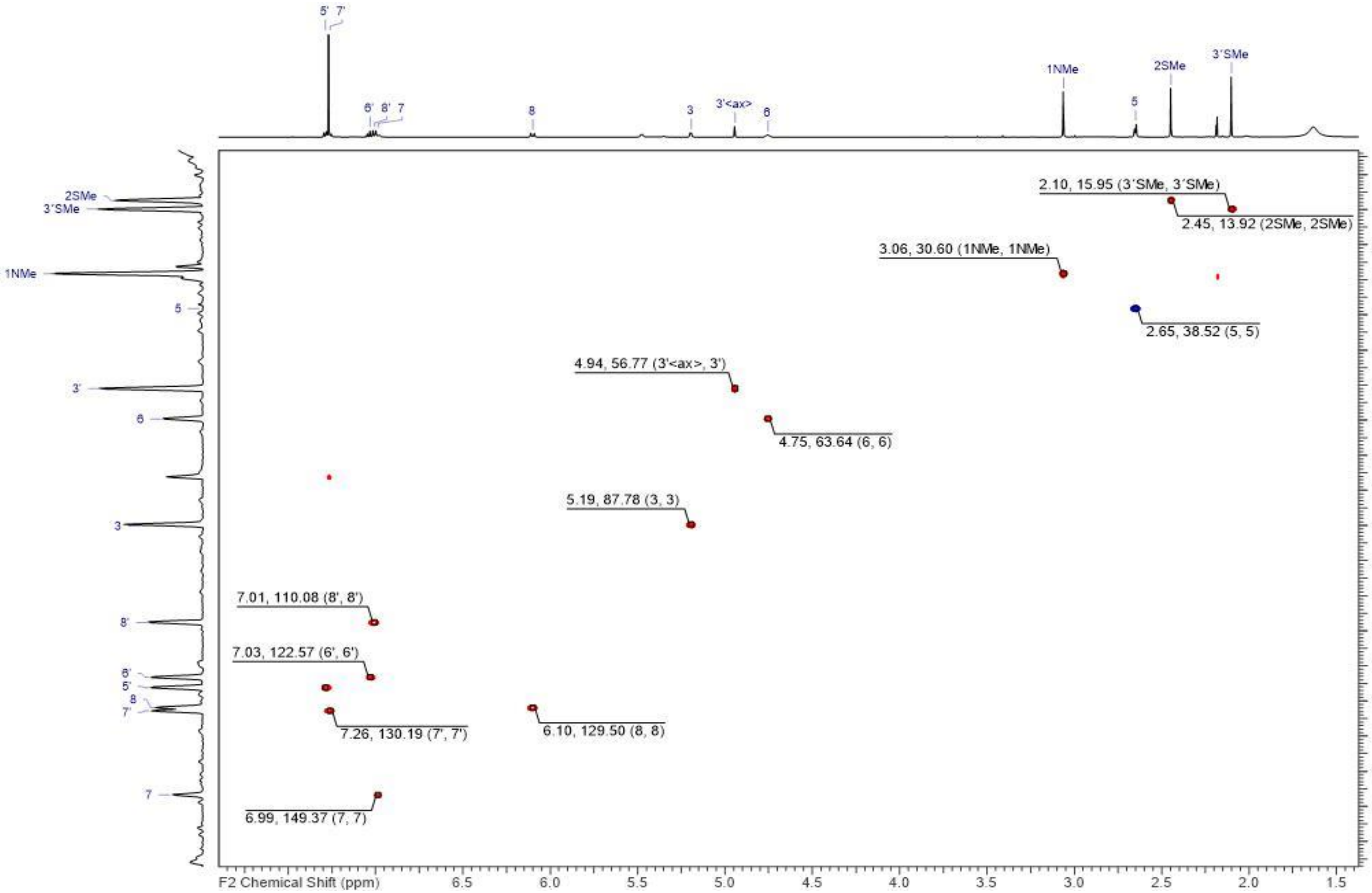

Figure S23. HSQC-DEPT NMR spectrum of **4** in $CDCl_3$ (500 MHz).

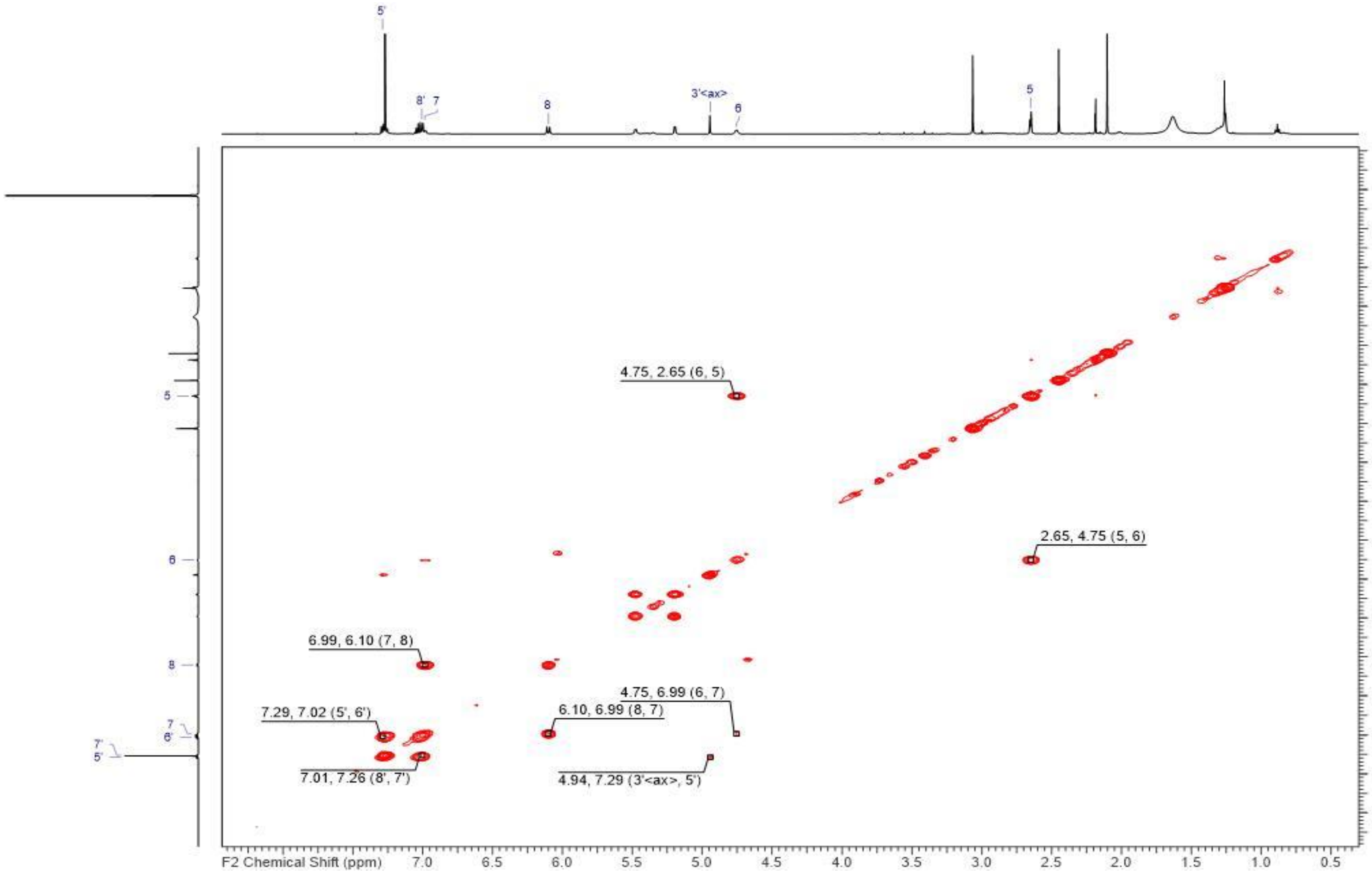

Figure S24. COSY NMR spectrum of **4** in $CDCl_3$ (500 MHz).

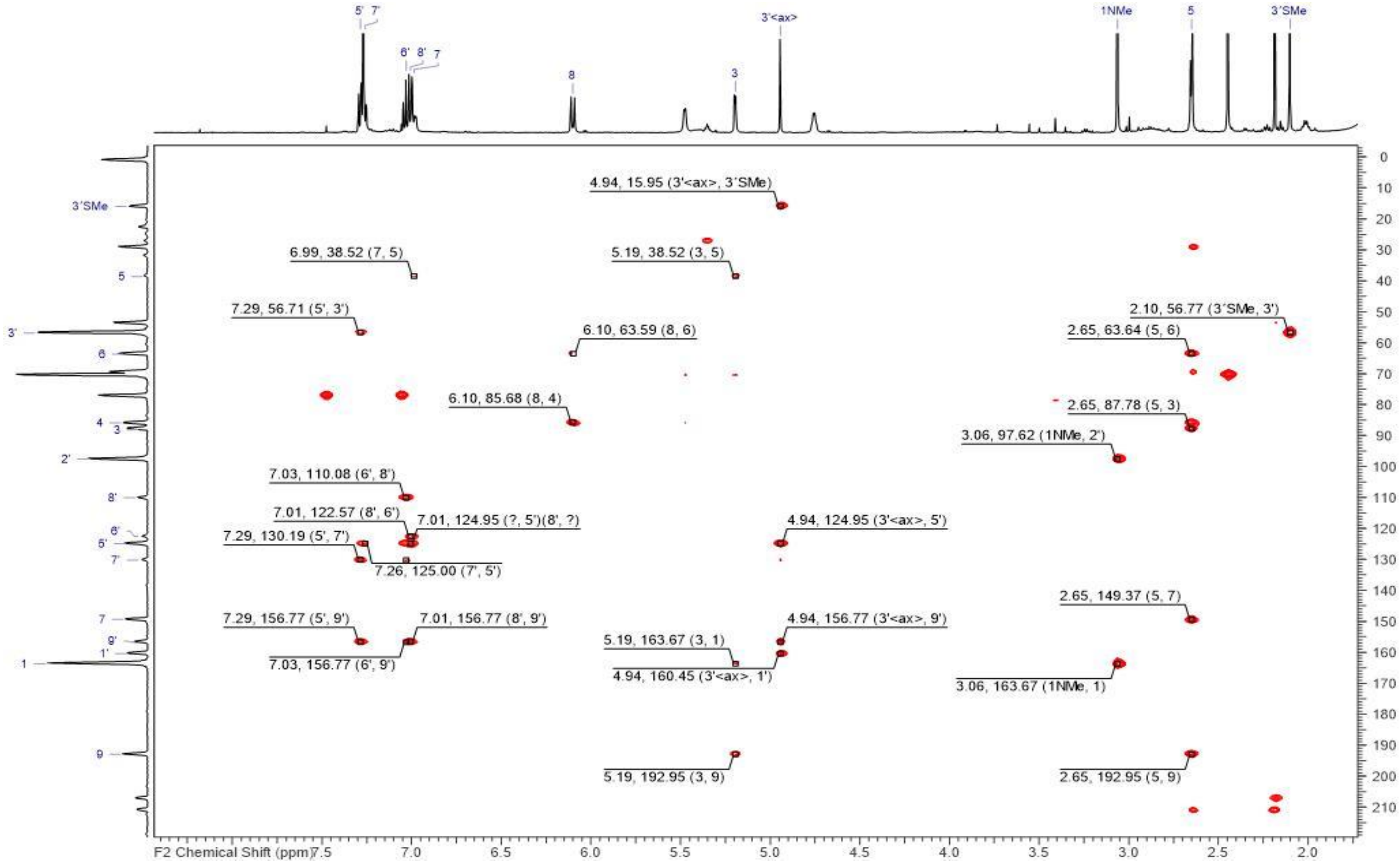

Figure S25. HMBC NMR spectrum of **4** in $CDCl_3$ (500 MHz).

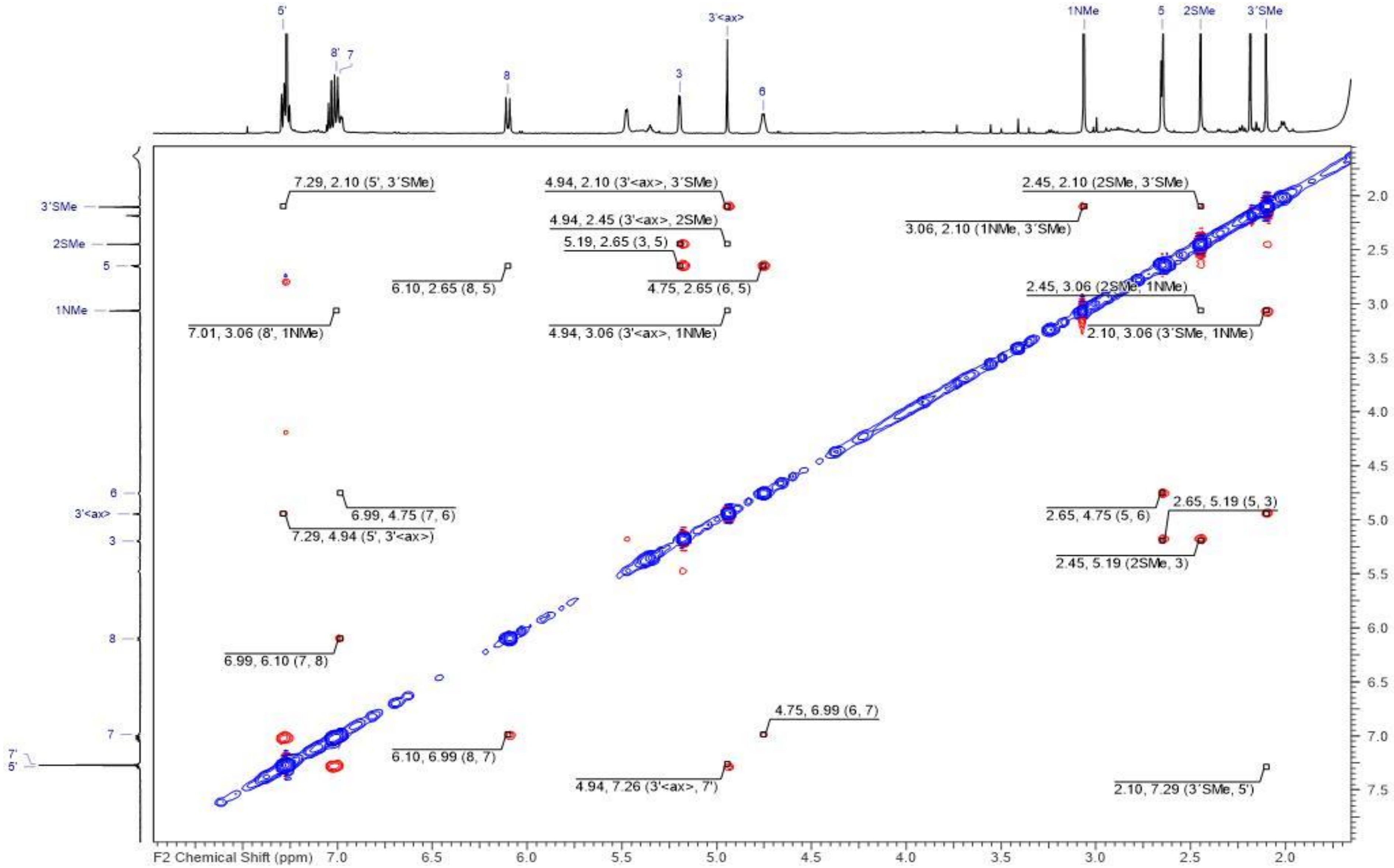

Figure S26. ROESY NMR spectrum of **4** in $CDCl_3$ (500 MHz).

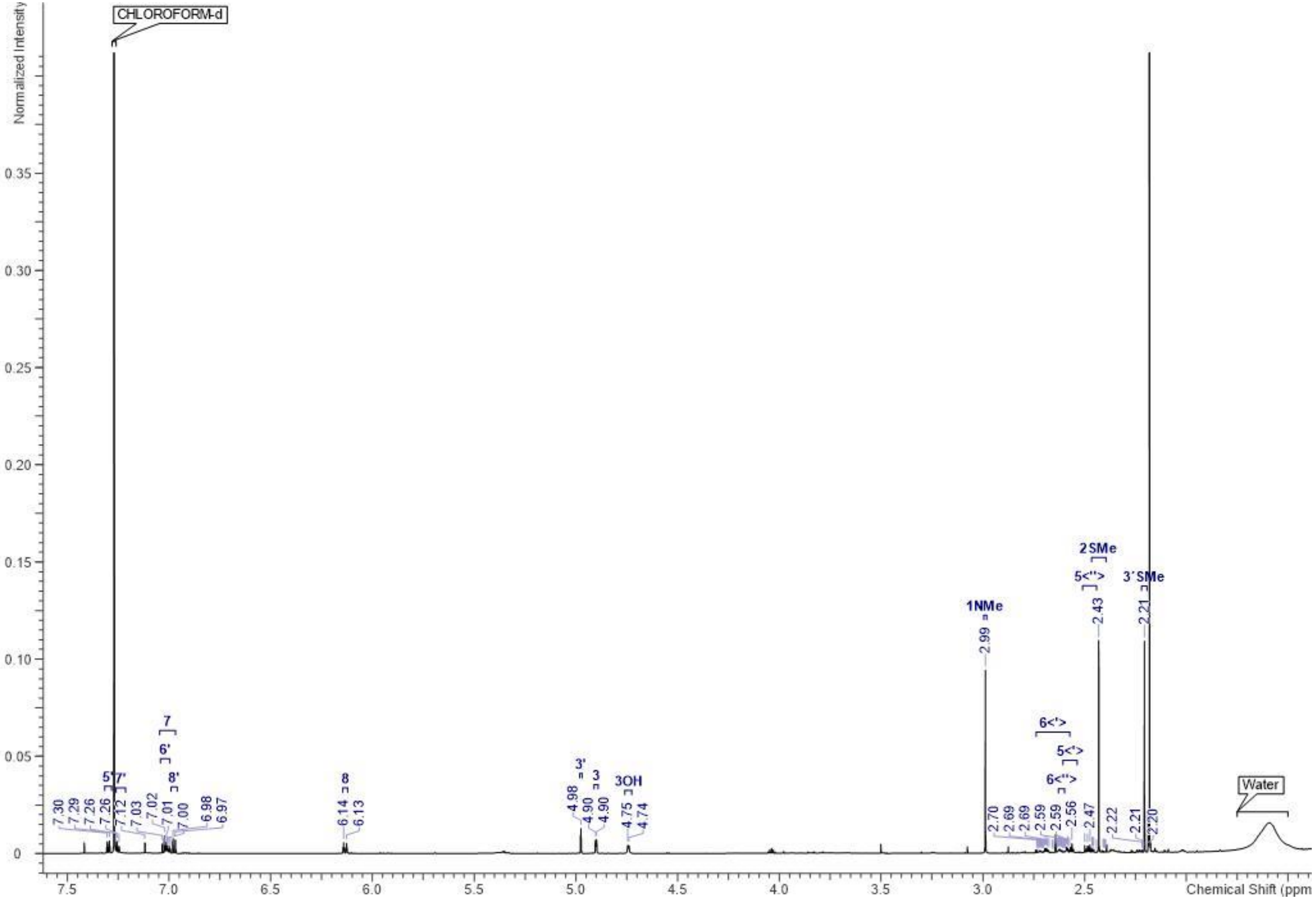

Figure S27. ^{1}H NMR spectrum of **5** in $CDCl_3$ (700 MHz).

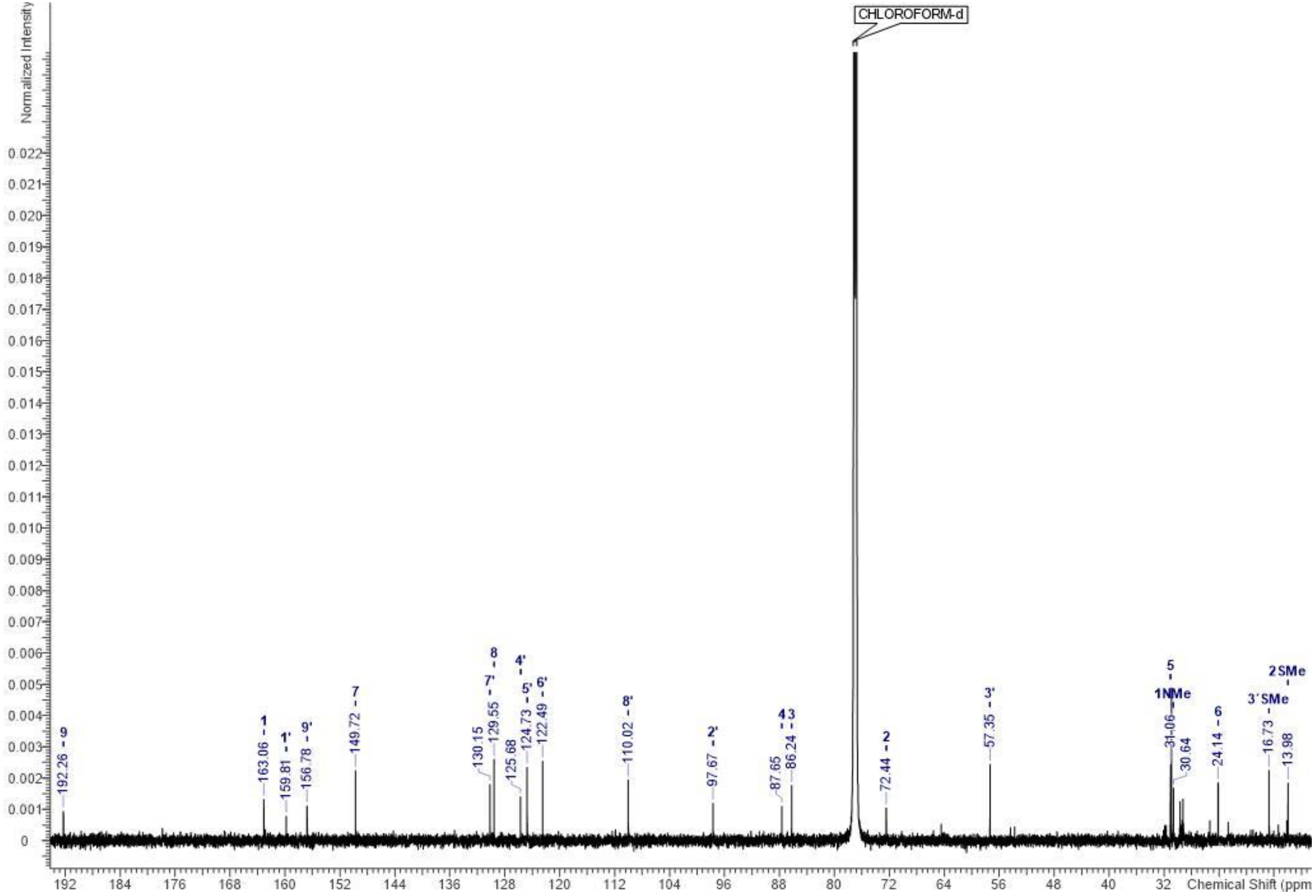

Figure S28. ^{13}C NMR spectrum of **5** in $CDCl_3$ (700 MHz).

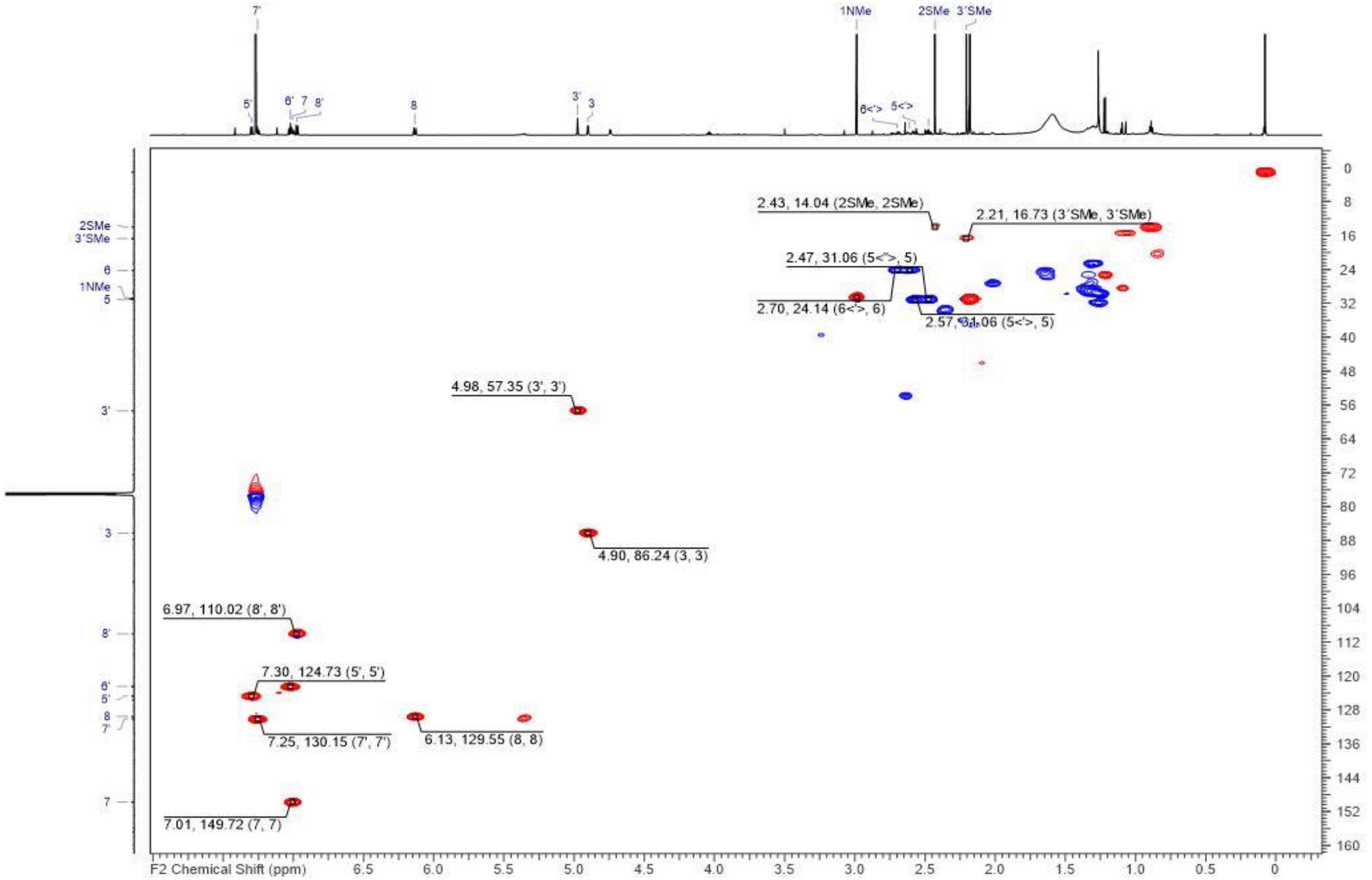

Figure S29. HSQC-DEPT NMR spectrum of **5** in $CDCl_3$ (700 MHz).

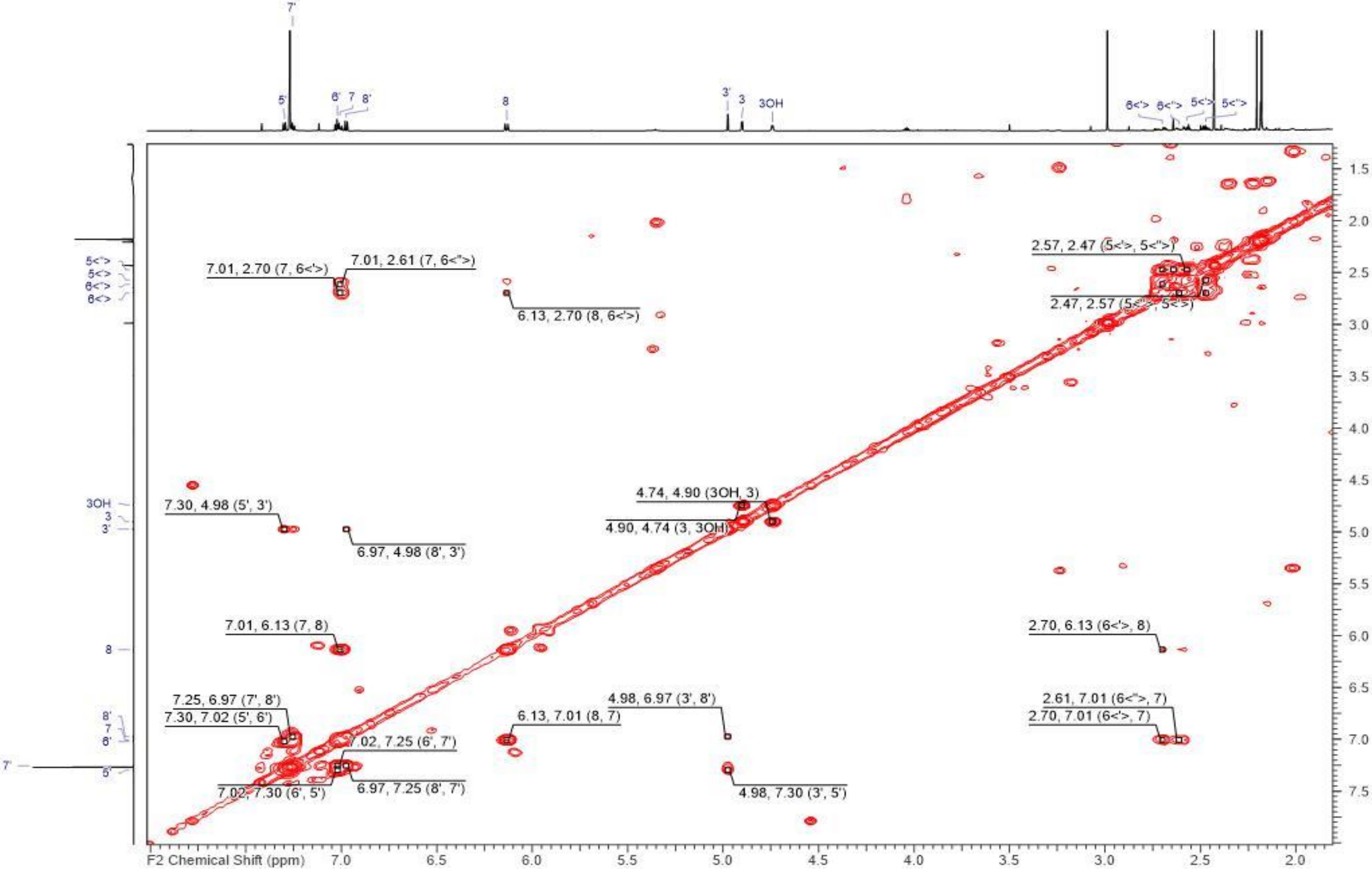

Figure S30. COSY NMR spectrum of **5** in $CDCl_3$ (700 MHz).

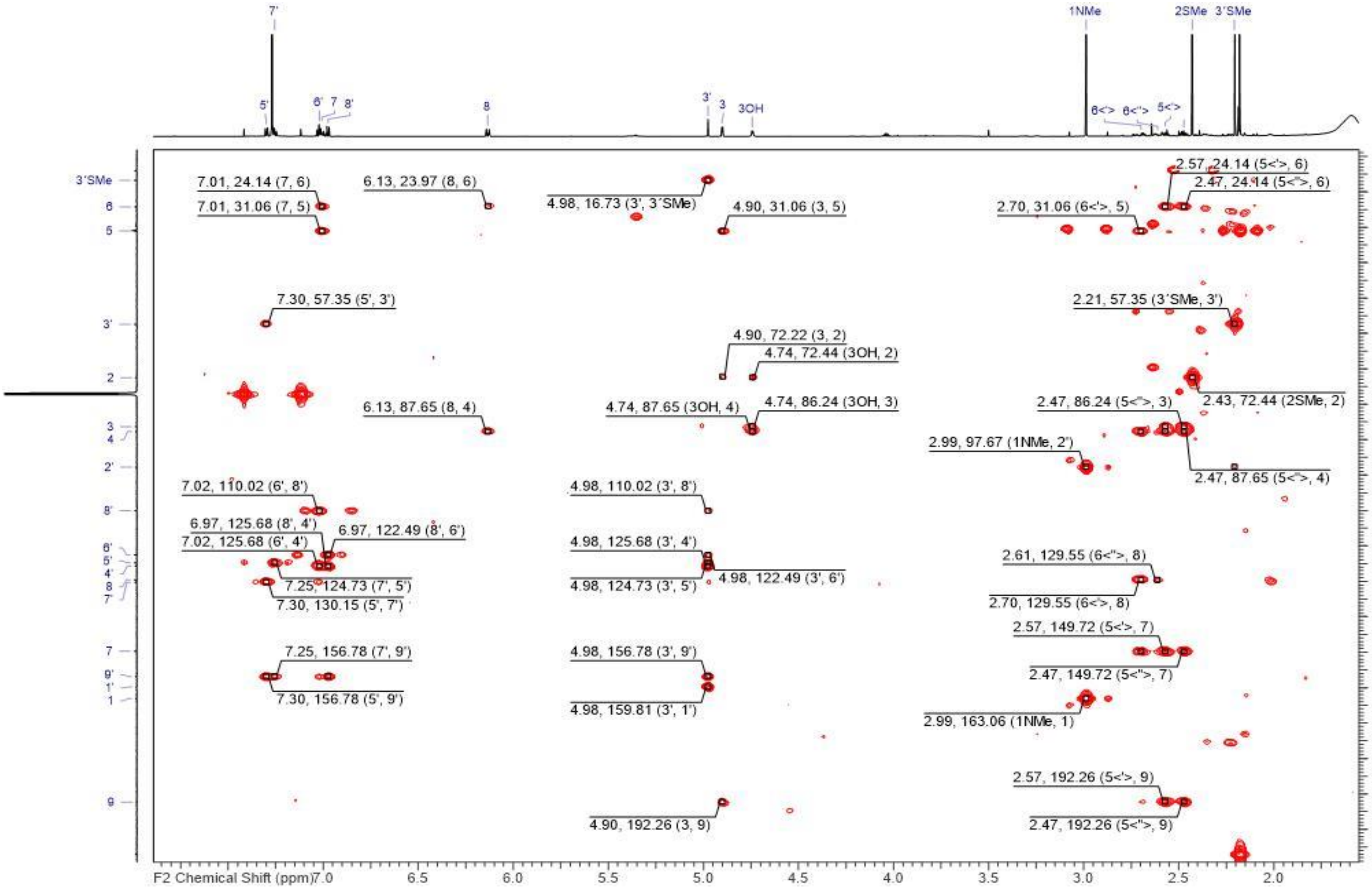

Figure S31. HMBC NMR spectrum of **5** in $CDCl_3$ (700 MHz).

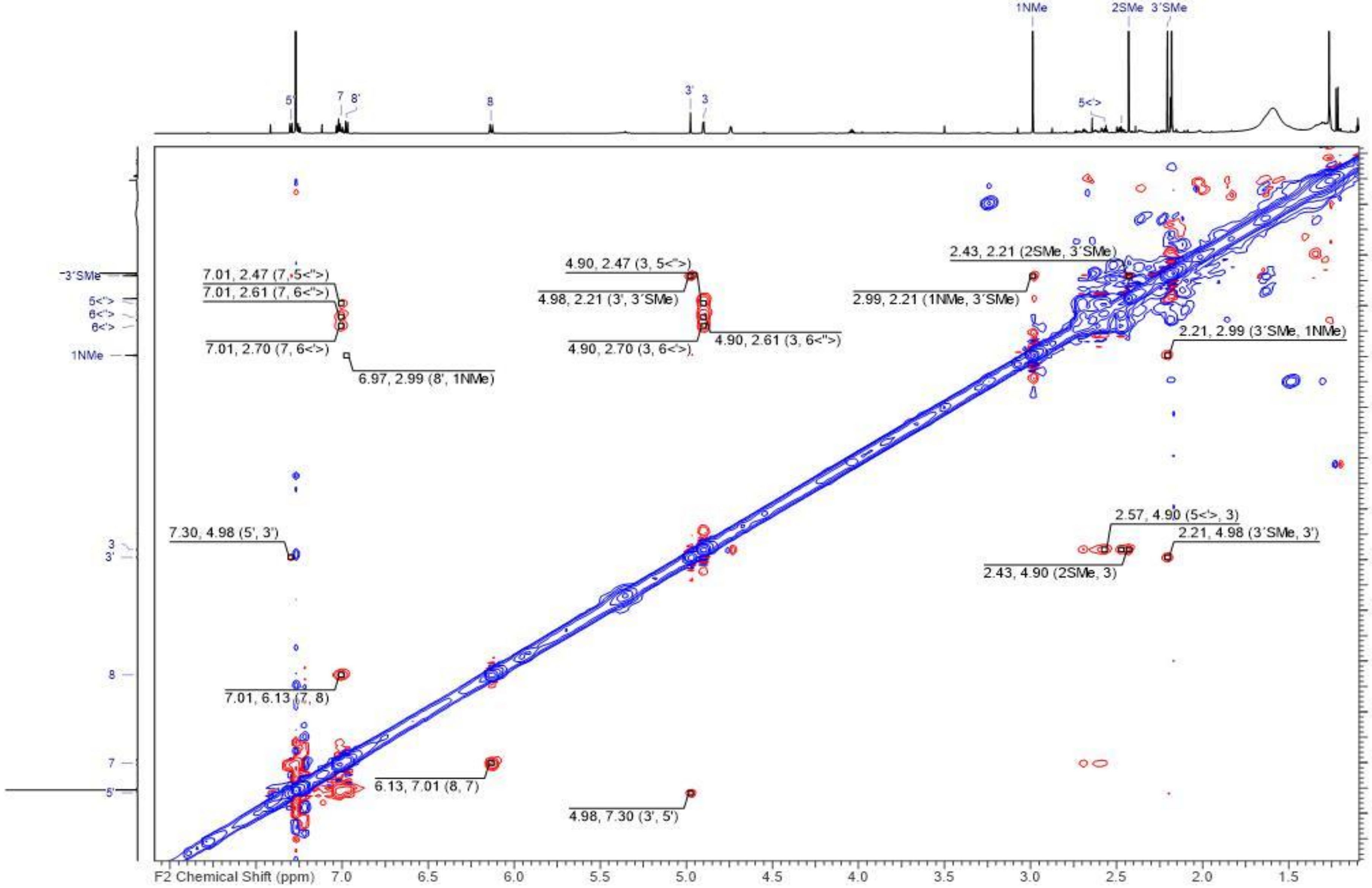

Figure S32. ROESY NMR spectrum of **5** in $CDCl_3$ (700 MHz).

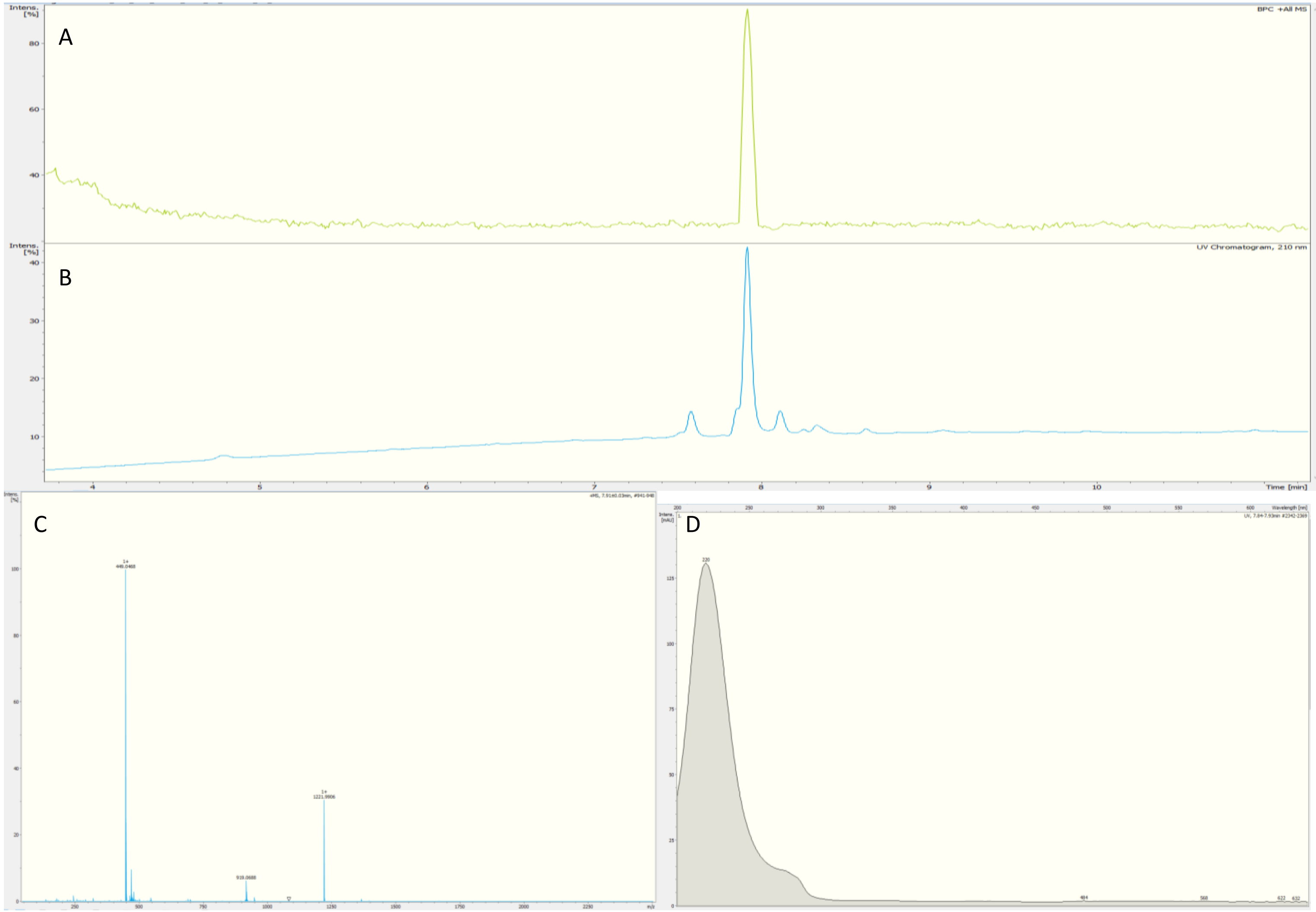

Figure S33. HRESIMS of compound **1** isolated from BRFT medium. A is the positive mass spectra. B is the chromatogram at 210 nm and C is the compound mass. D is the extracted UV spectrum of **1**.

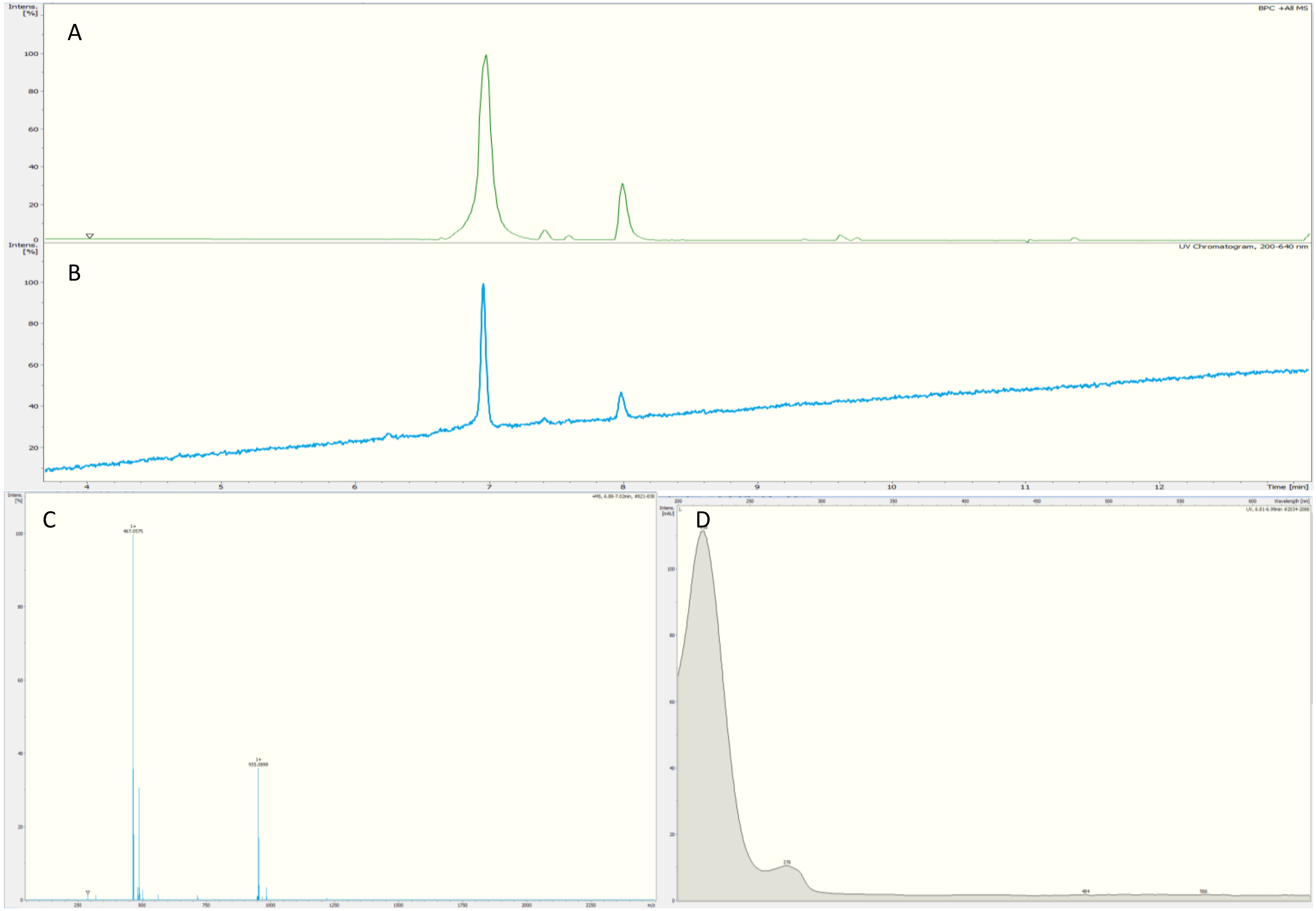

Figure S34. HRESIMS of compound **2** isolated from BRFT medium. A is the positive mass spectra. B is the chromatogram at 210 nm and C is the compound mass. D is the extracted UV spectrum of **2**.

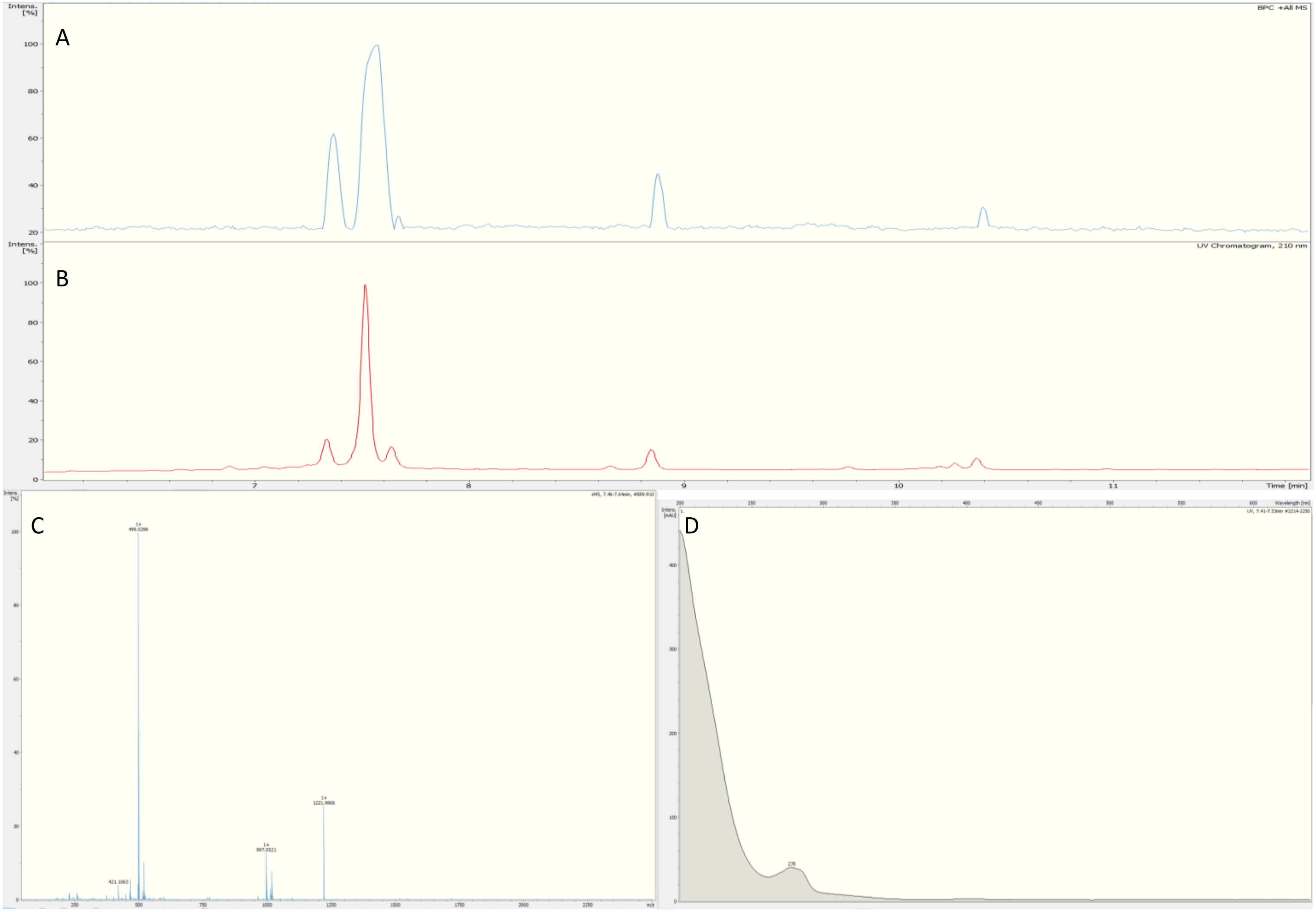

Figure S35. HRESIMS of compound **3** isolated from BRFT medium. A is the positive mass spectra. B is the chromatogram at 210 nm and C is the compound mass. D is the extracted UV spectrum of **3**.

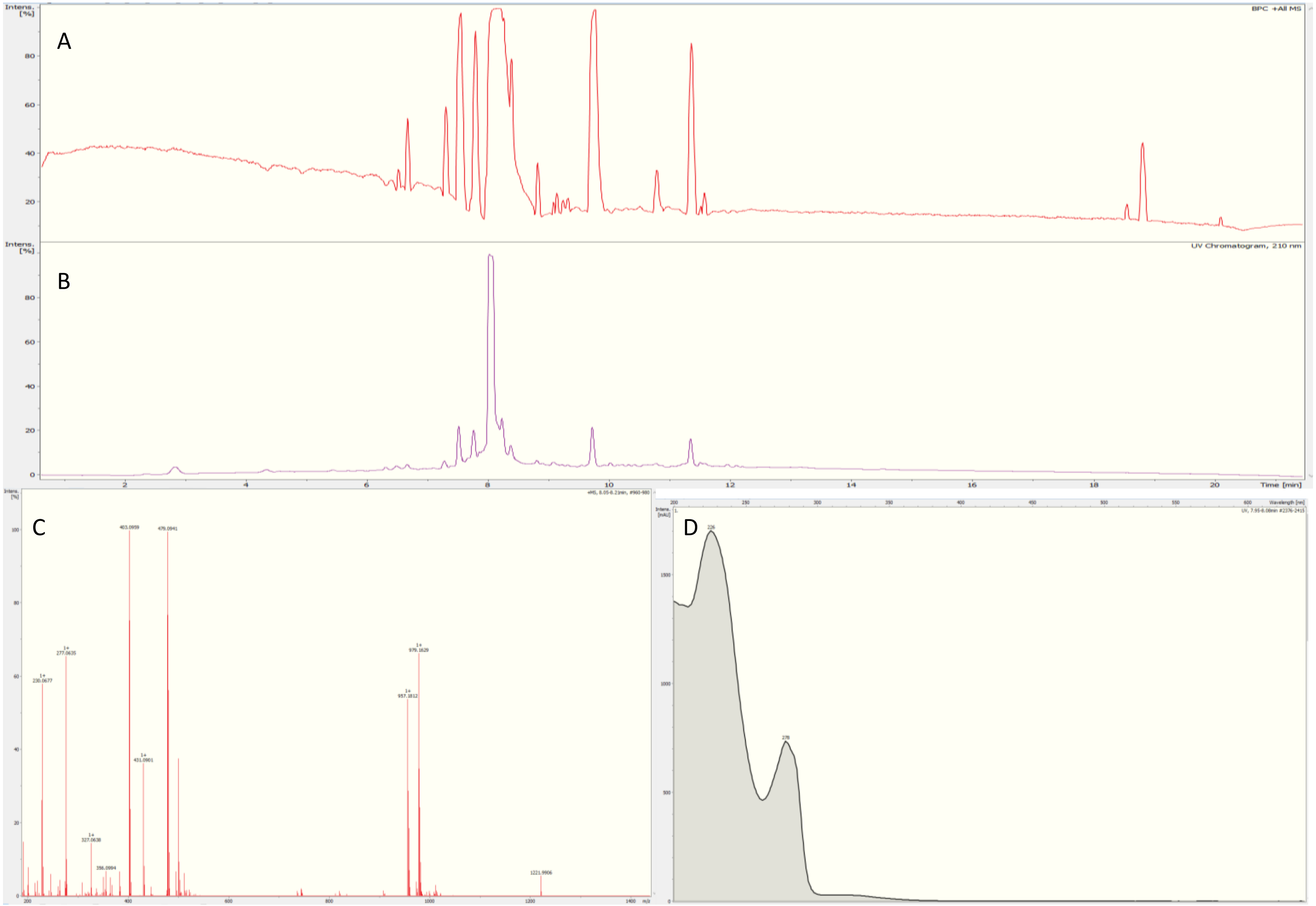

Figure S36. HRESIMS of compound **4** isolated from BRFT medium. A is the positive mass spectra. B is the chromatogram at 210 nm and C is the compound mass. D is the extracted UV spectrum of **4**.

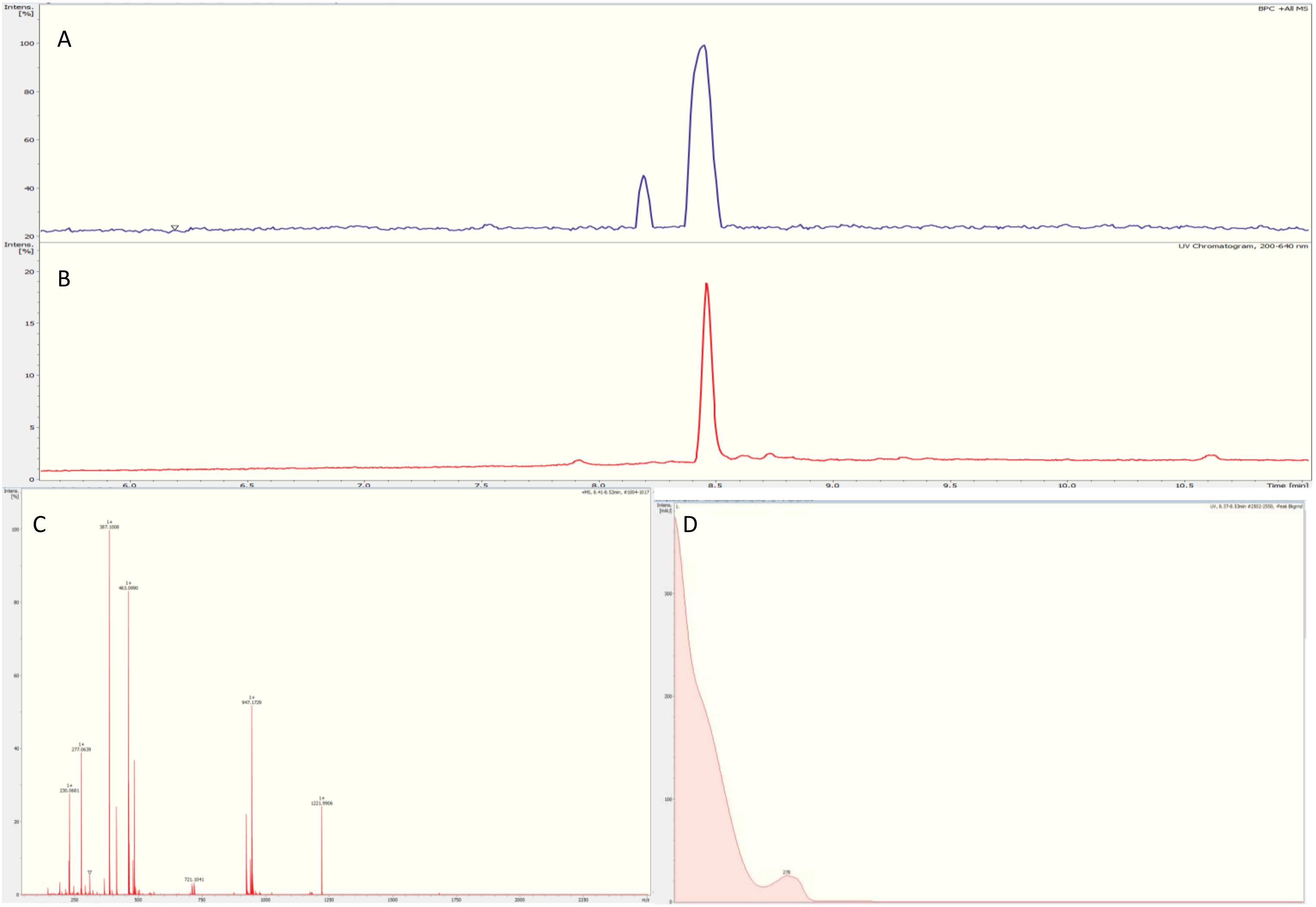

Figure S37. HRESIMS of compound **5** isolated from BRFT medium. A is the positive mass spectra. B is the chromatogram at 210 nm and C is the compound mass. D is the extracted UV spectrum of **5**.

Table S2. NMR data (^{1}H 700 MHz, ^{13}C 175 MHz) of compound **7** and **8**.

Atom	**7** (in DMSO-d_6) δ$_C$, type	δ$_H$, (mult, J, Hz)	**8** (in DMSO-d_6) δ$_C$, type	δ$_H$, (mult, J, Hz)
1	143.6, C	-	151.4, C	-
1 OCH_3	56.1, CH_3	3.78, s	56.3, CH_3	3.71, s
2	107.6, CH	6.83, d (9.2)	108.6, CH	6.75, d(8.8)
3	116.8, CH	7.19, d (9.0)	119.9, CH	7.00, d(8.8)
4	152.2, C	-	141.2, C	-
4a	148.0, C	-	146.5, C	-
4 OCH_3	56.2, CH_3	3.73, s	-	-
6	75.0, CH_2	5.16, s	75.3, CH_2	5.14, s
6a	140.1, C	-	140.5, C	-
7	118.3, CH	6.67, d (0.8)	118.3, CH	6.69, s
8	116.8, C	-	145.6, C	-
9	116.9, CH	6.78, s	116.9, CH	6.79, br s
10	160.9, C	-	161.5, C	-
10a	118.0, C	-	118.2, C	-
11	193.3, C	-	193.8, C	-
11a	122.3, C	-	122.3, C	-
12	20.8, CH_3	2.29, s	20.8, CH_3	2.29, s

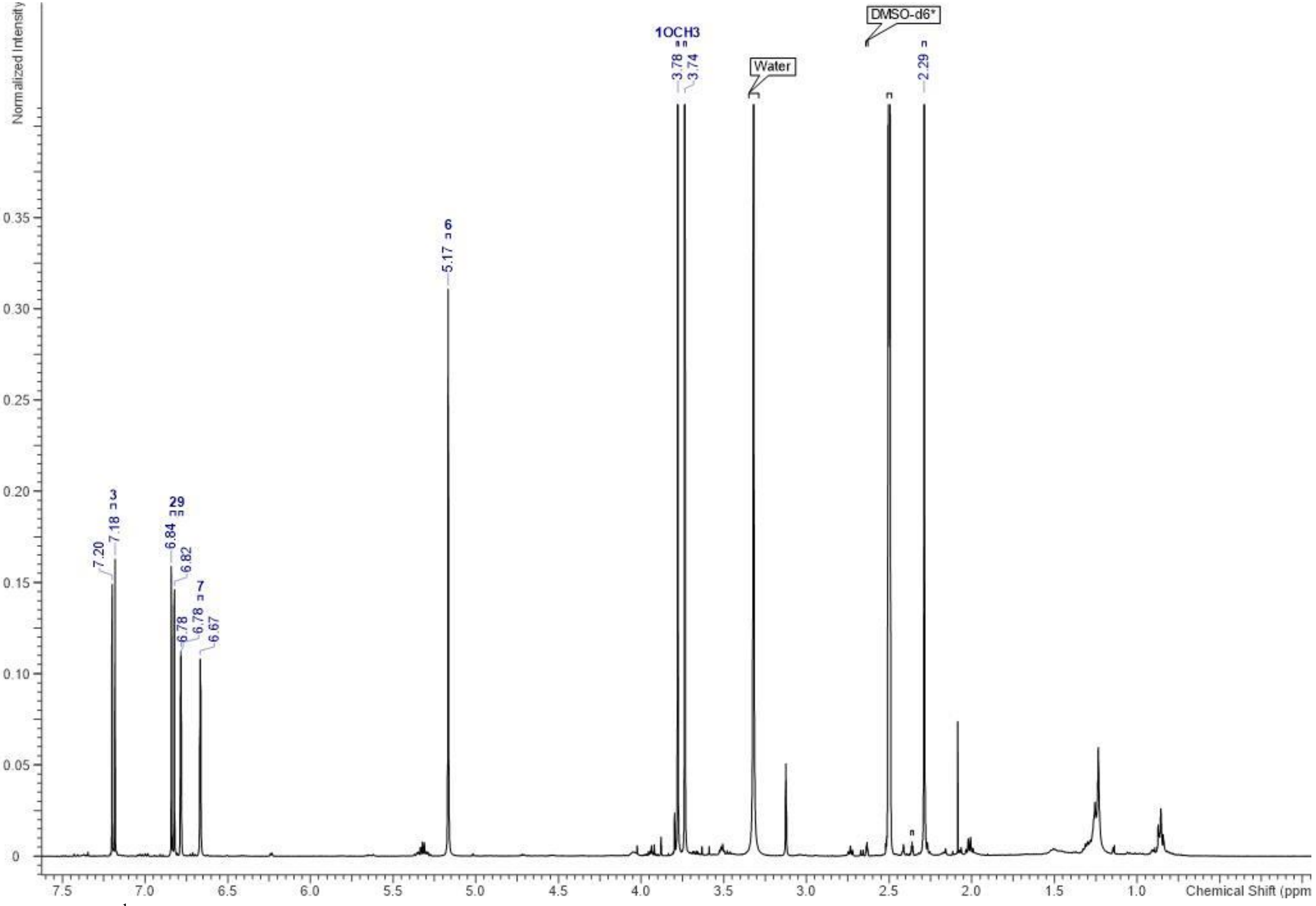

Figure S38. ^{1}H spectrum of **7** in DMSO-d_6 (500 MHz).

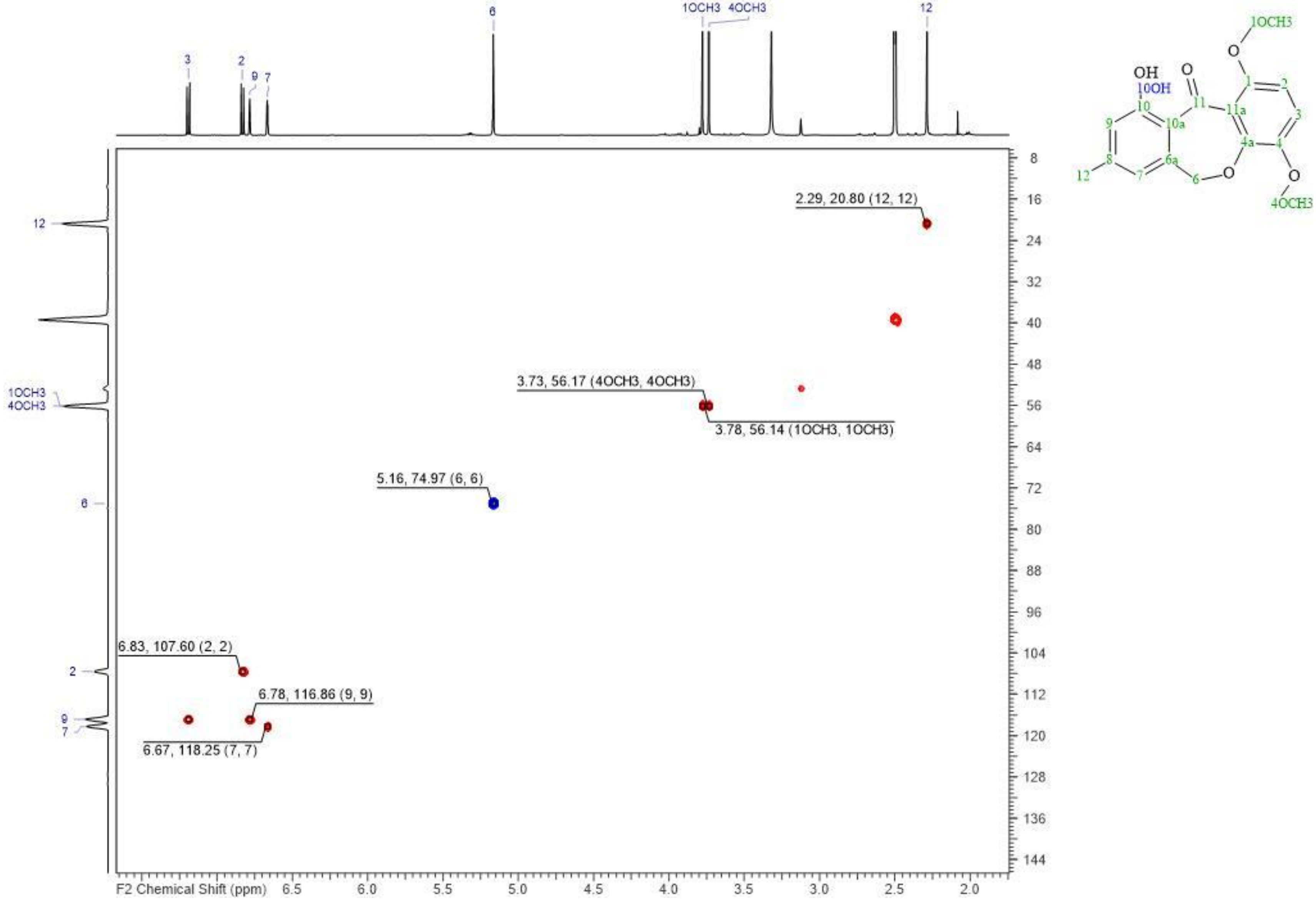

Figure S39. HSQC spectrum of **7** in DMSO-d_6 (500 MHz).

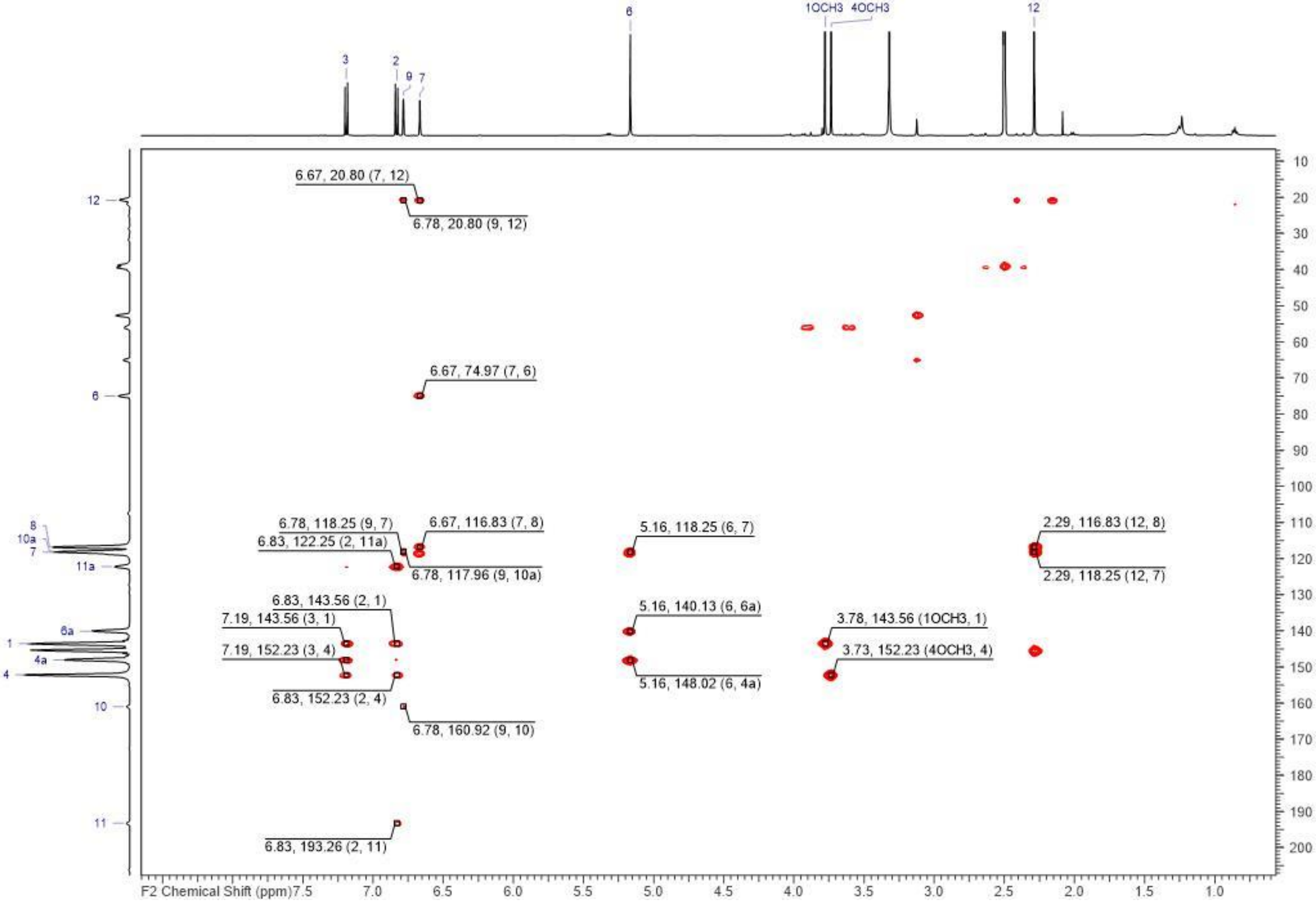

Figure S40. HMBC spectrum of **7** in DMSO-d_6 (500 MHz).

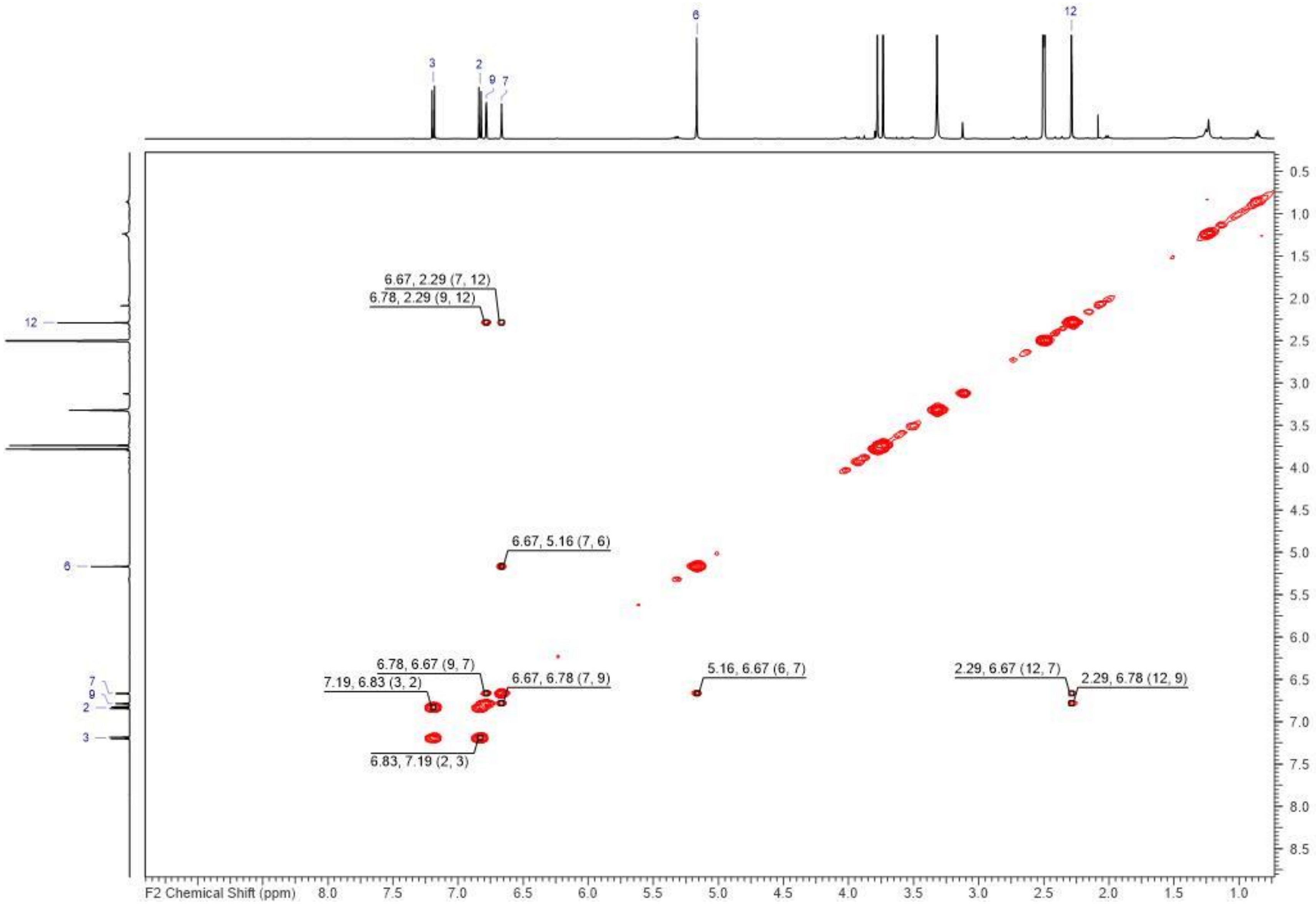

Figure S41. COSY spectrum of **7** in DMSO-d_6 (500 MHz).

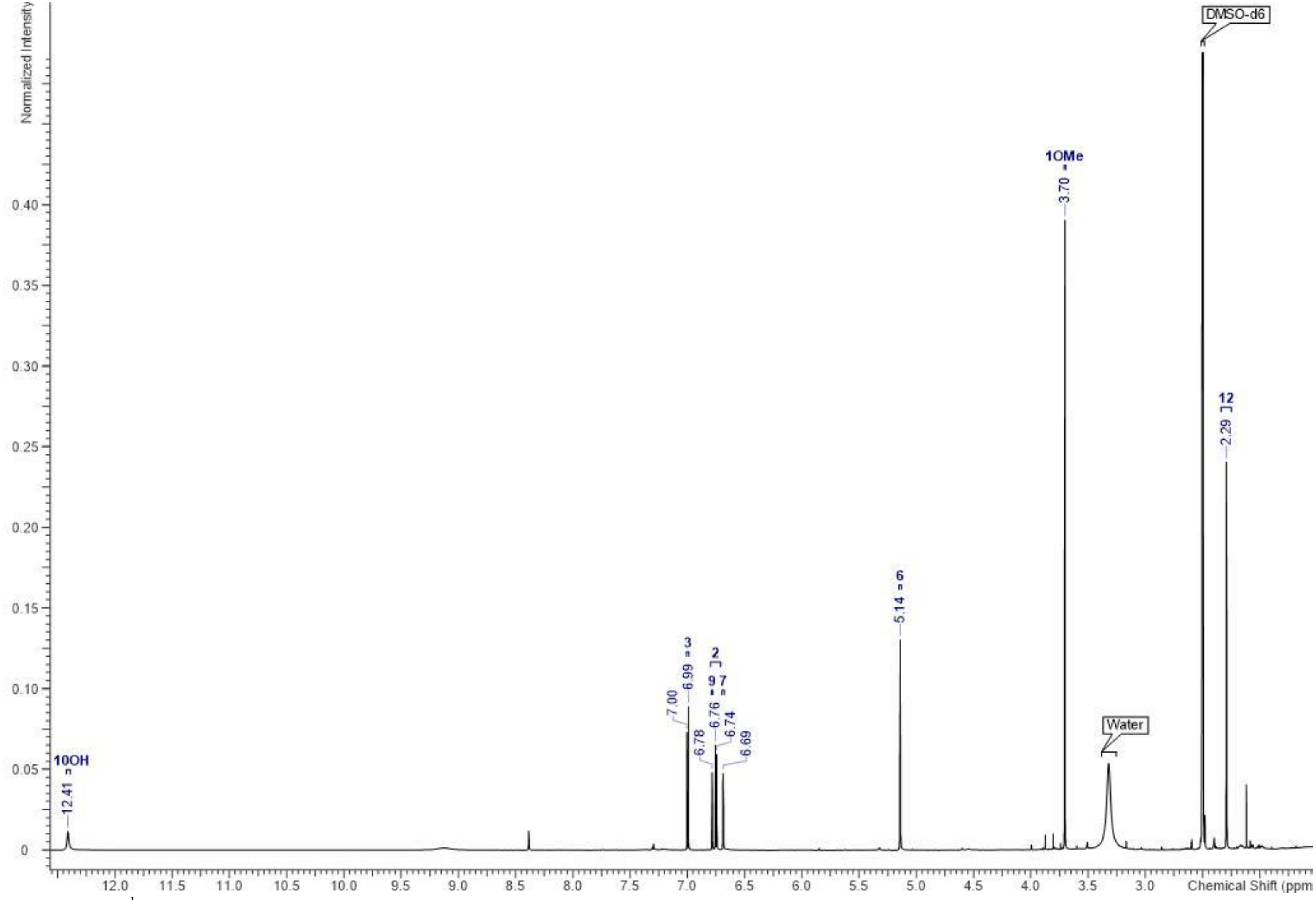

Figure S42. ^{1}H spectrum of **8** in DMSO-d_6 (700 MHz).

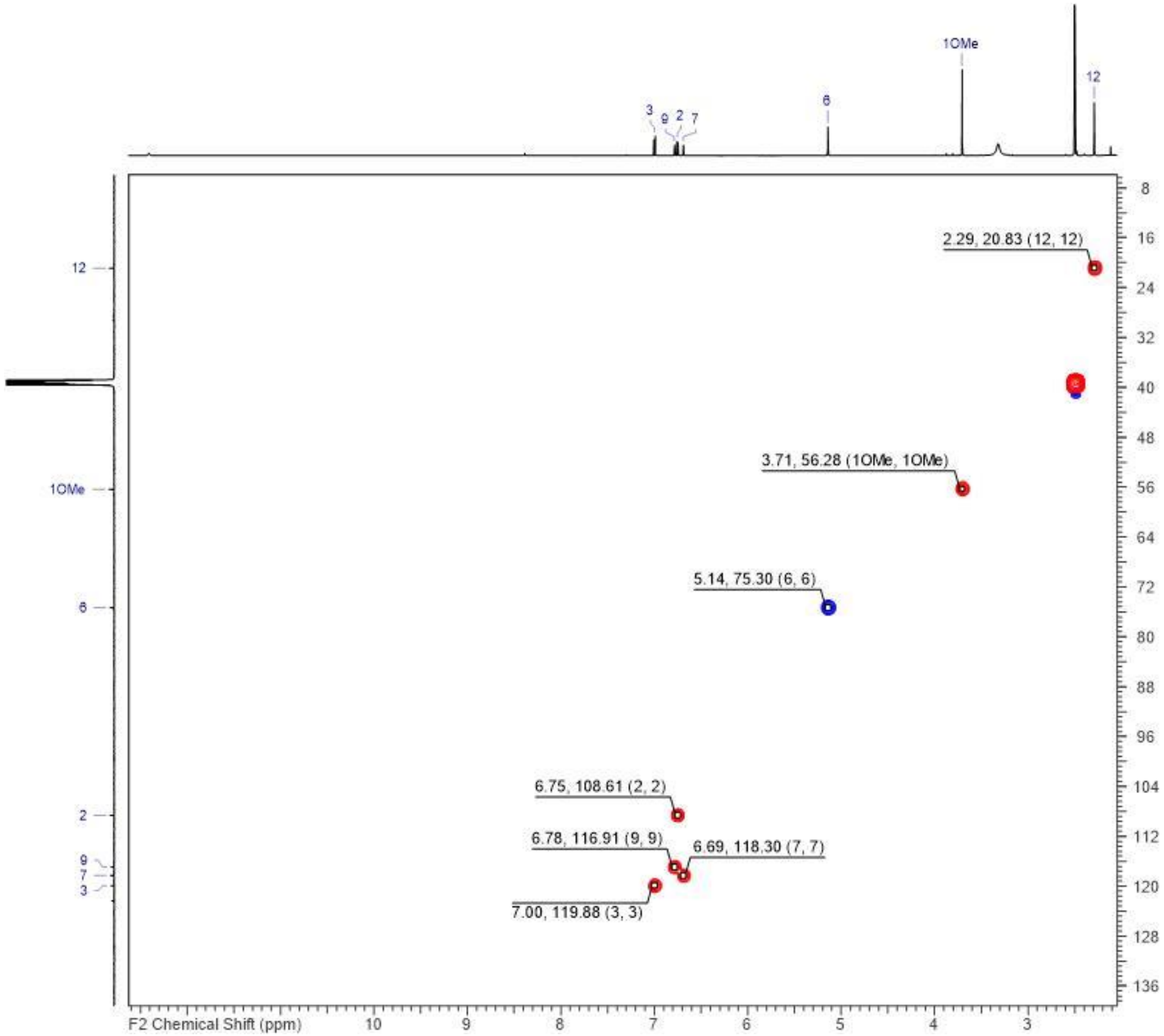

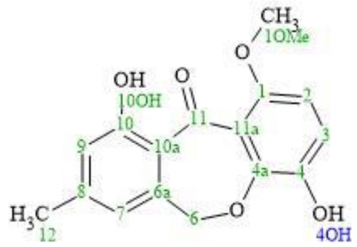

Figure S43. HSQC spectrum of **8** in DMSO-d_6 (700 MHz).

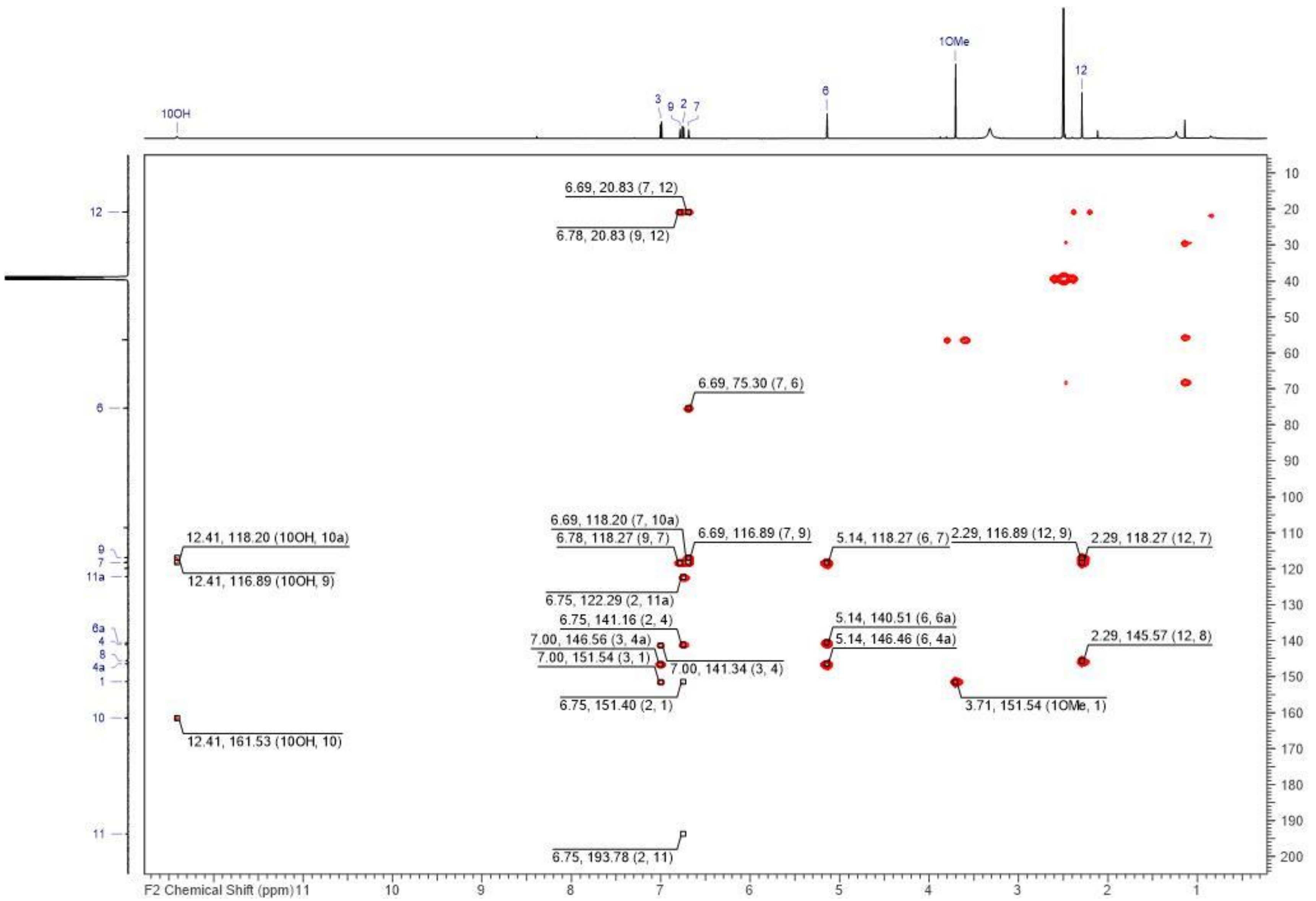

Figure S44. HMBC spectrum of **8** in DMSO-d_6 (700 MHz).

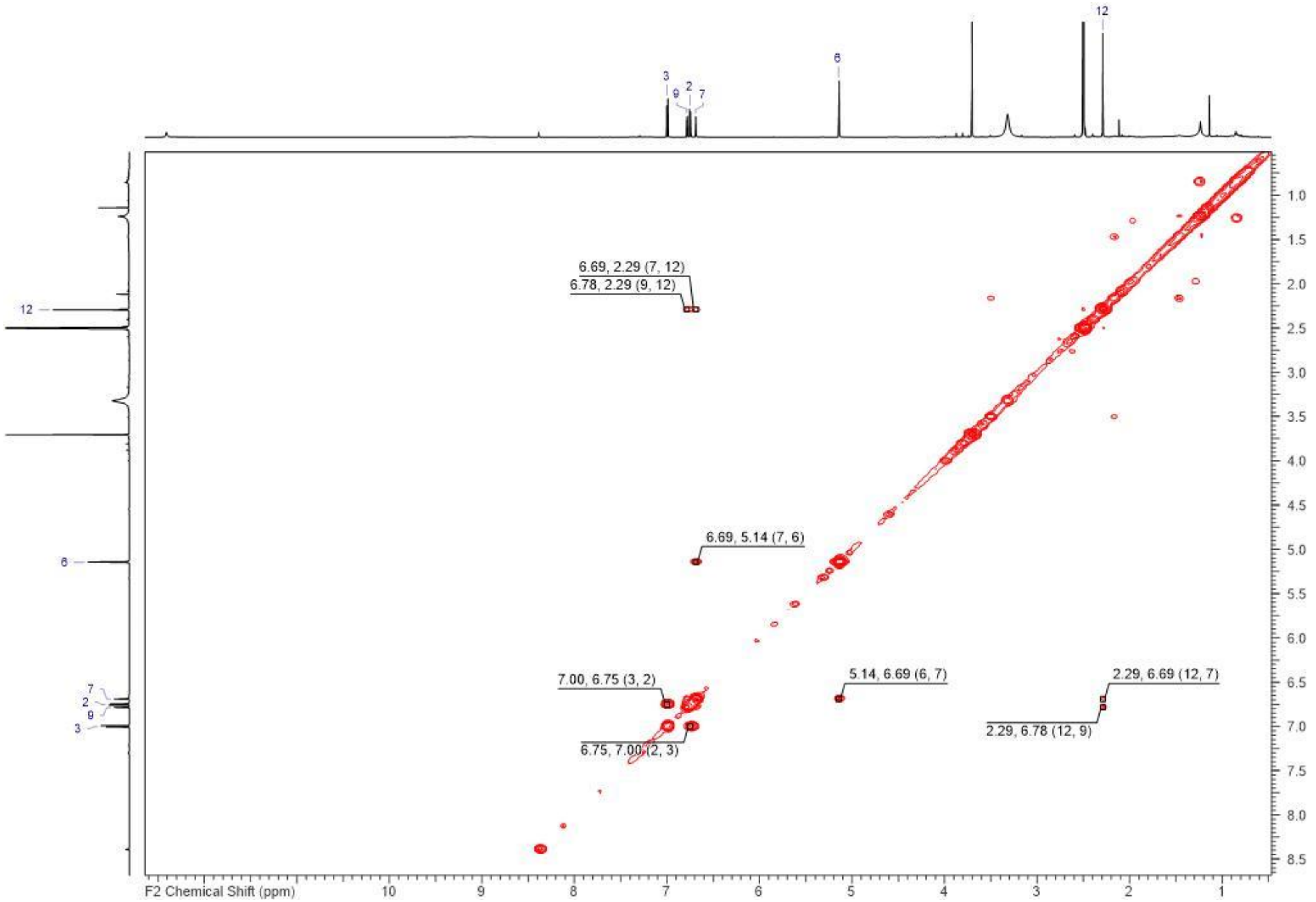

Figure S45. COSY spectrum of **8** in DMSO-d_6 (700 MHz).

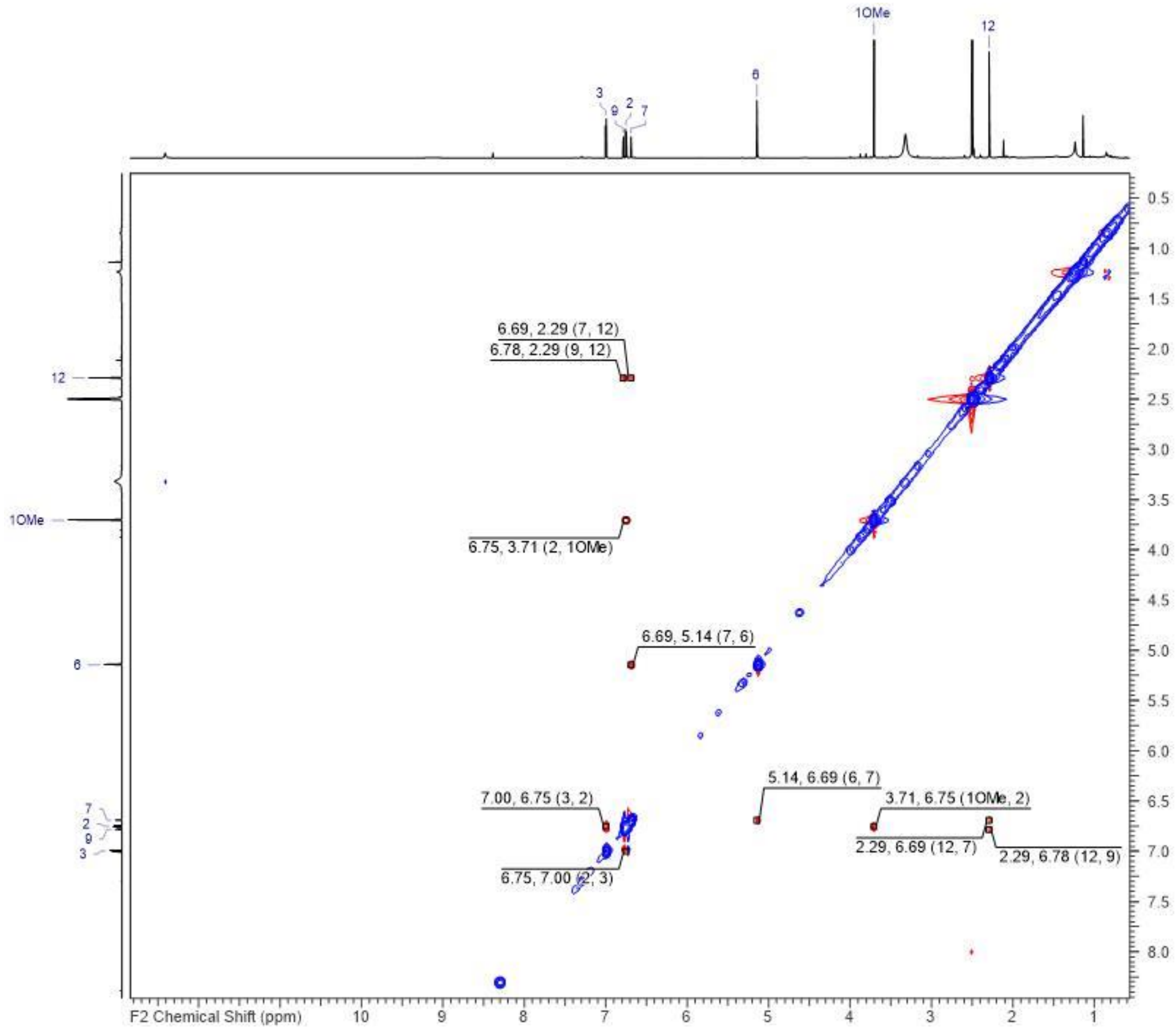

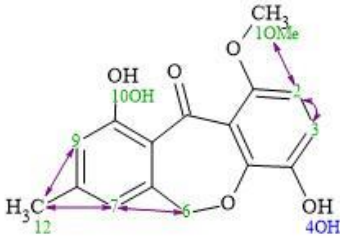

Figure S46. ROESY spectrum of **8** in DMSO-d_6 (700 MHz).

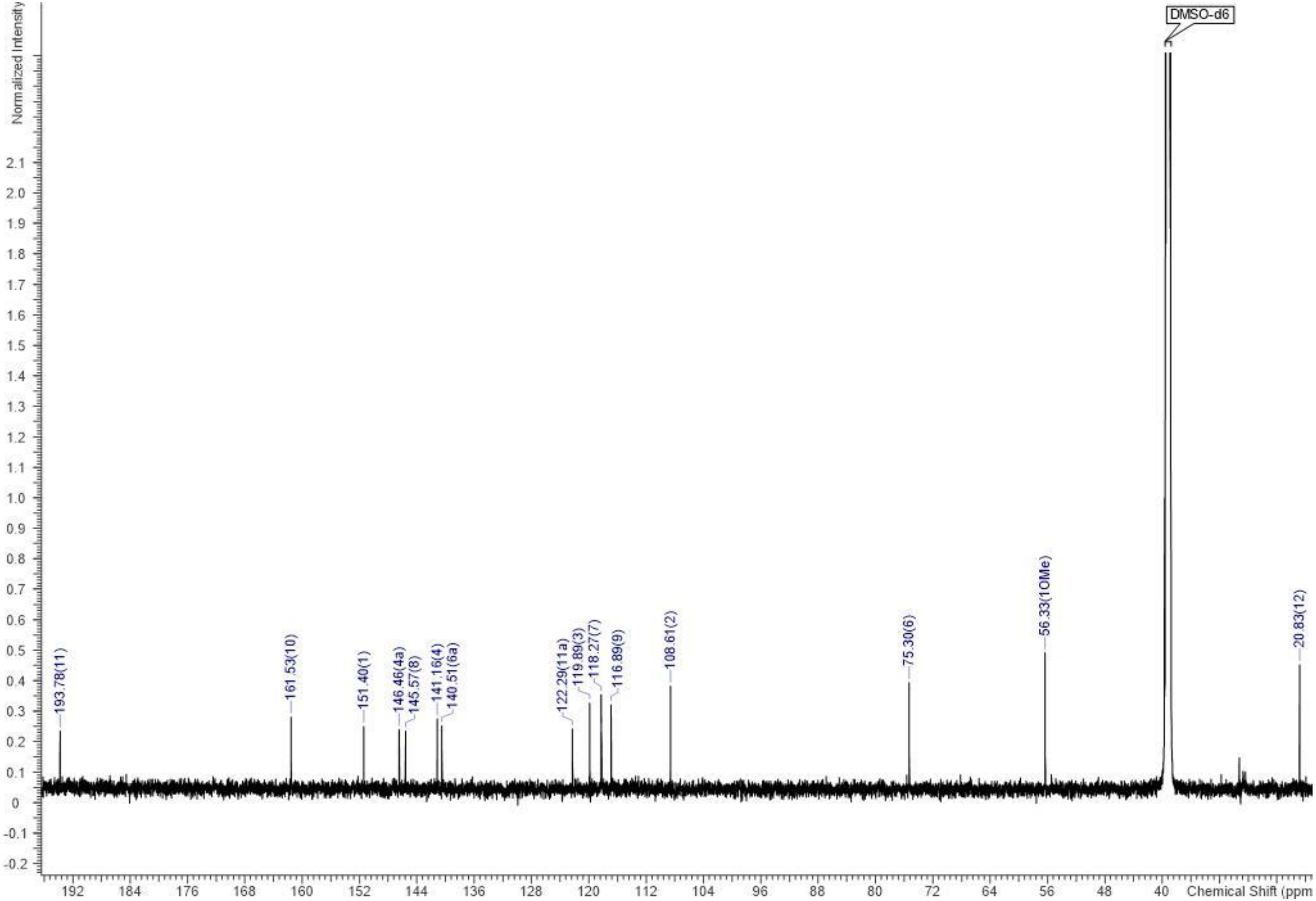

Figure S47. ^{13}C spectrum of **8** in DMSO-d_6 (700 MHz).

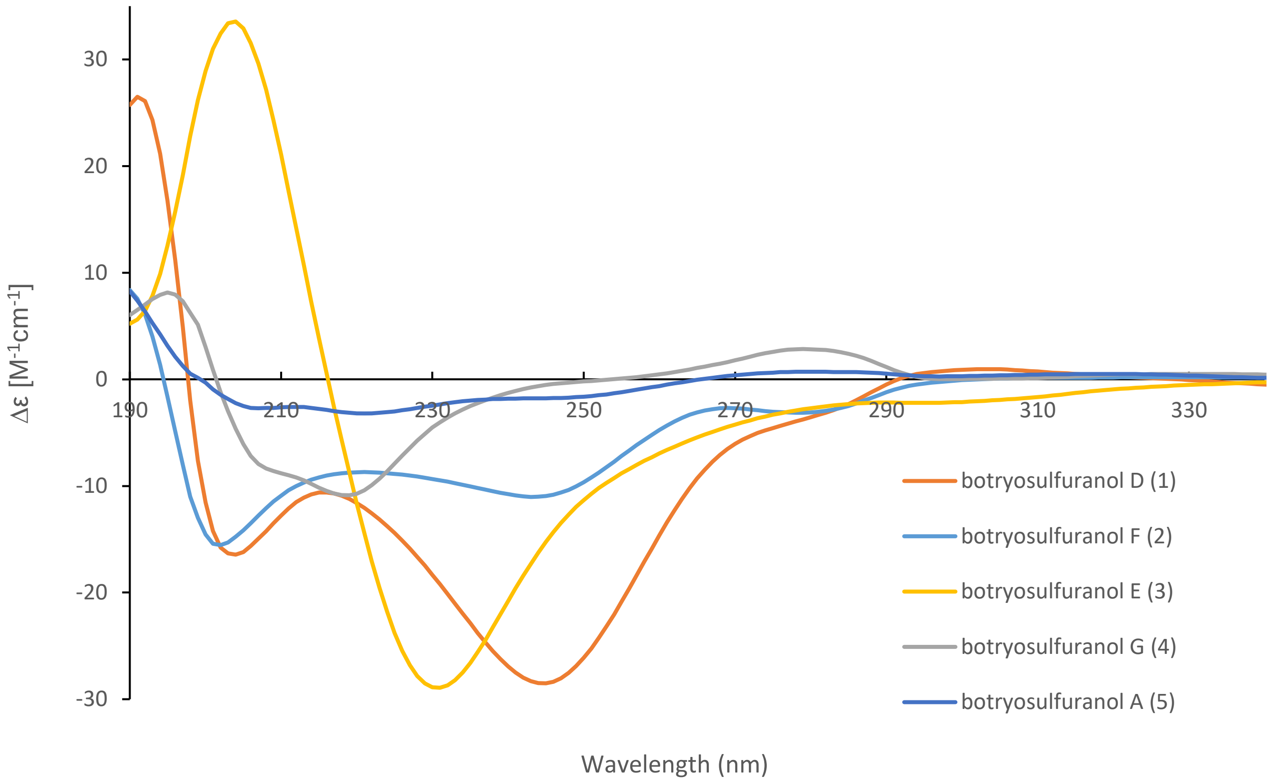

Figure S48. Experimental ECD spectra of compound **1**–**5**.
